# Early Functional Postoperative Therapy of Distal Radius Fracture with a Dynamic Orthosis: Results of a Prospective Randomized Cross-Over Comparative Study

**DOI:** 10.1371/journal.pone.0117720

**Published:** 2015-03-30

**Authors:** Fabian M. Stuby, Stefan Döbele, Susanne-Dorothea Schäffer, Simon Mueller, Atesch Ateschrang, Matthias Baumann, Derek Zieker

**Affiliations:** 1 BG Trauma Center, University of Tuebingen, Schnarrenbergstrasse 95, D-72076, Tuebingen, Germany; 2 Muon-Stat Statistical Services, Klugestrasse 28, D-70197, Stuttgart, Germany; 3 Paracelsus Hospital Ruit, Hedelfinger Strasse 166, D-73760, Ostfildern, Germany; 4 Department of General Surgery, University of Tübingen, Hoppe-Seyler-Strasse 3, D-72076 Tübingen, Germany; The University of Tokyo Hospital, JAPAN

## Abstract

**Introduction:**

This study was conducted according to GCP criteria as a prospective randomized cross-over study. The primary goal of the study was to determine clinical findings and patient satisfaction with postoperative treatment. 29 patients with a distal radius fracture that was surgically stabilized from volar and who met the inclusion criteria were enrolled over a 12-month period. Each patient randomly received either a dorsal plaster splint or a vacuum-fit flexible but blocked orthosis applied postoperatively in the operating theatre to achieve postoperative immobilization. After one week all patients were crossed over to the complementary device maintaining the immobilization until end of week 2. After week 2 both groups were allowed to exercise wrist mobility with a physiotherapist, in the orthosis group the device was deblocked, thus allowing limited wrist mobility. After week 4 the devices were removed in both groups. Follow-up exams were performed after postoperative weeks 1, 2, 4 and 12.

**Results and Discussion:**

Results were determined after week 1 and 2 using SF 36 and a personally compiled questionnaire; after weeks 4 and 12 with a clinical check-up, calculation of ROM and the DASH Score. Comparison of the two groups showed a significant difference in ROM for volar flexion after 4 weeks, but no significant differences in DASH Score, duration of disability or x-ray findings. With regard to satisfaction with comfort and hygiene, patients were significantly more satisfied with the dynamic orthosis, and 23 of the 29 patients would prefer the flexible vacuum orthosis in future.

**Trial Registration:**

German Clinical Trials Register (DRKS) DRKS00006097

## Introduction

The distal radius fracture is one of the most common injuries of the human skeleton. It has an incidence of 31 per 104 person-years and in some countries it is the most common fracture occurring in elderly, namely almost 500 per 100.000 [1]. The literature reports that this fracture accounts for 25% of all fractures in adolescent and for 18% of all fractures in the elderly. In the United States alone more than 640.000 radius fractures were documented in 2001 [2]. While the incidence of this fracture has not changed considerably since 1997, the frequency of surgical therapy and thus the number of in-hospital treatments for this injury has increased considerably [1,3]. For non-dislocated fractures conservative therapy with immobilization of the wrist in a plaster cast also called plaster of Paris for 6 weeks [4,5] is usually described. For displaced fractures the recent literature mostly recommends surgery, largely performed with plates [6], whose increasing availability also contributes to the increase in surgical treatment for this fracture [7,8]. But there are also tendencies to treat these fractures conservatively, as the results in elderly patients are described as comparable[9].

While surgical treatment has meanwhile become the gold standard [10,11] and depending on the type of fracture is usually performed from volar [12,13] under utilization of locking plates [14], to date hardly any studies have been conducted with regard to postoperative therapy and especially the effect of immobilization during the postoperative period. Some of the authors do not mention postoperative immobilization at all, some seem to completely dispense postoperative immobilization [15] and others perform postoperative immobilization in a circumferential cast or a splint for 2 weeks [16,17], three weeks [9] or even up to four weeks [14].

For treatment of injuries of the lower extremities various removable, sometimes vacuum-fit dynamic stabilizing systems are already available, some of which have also been evaluated [18]. In one study involving conventional therapy patients reported removable orthoses to provide clearly better wearing comfort [19].

Nevertheless, to date no data have been published on dynamic postoperative immobilization of the forearm and wrist following surgical stabilization of a distal radius fracture or the wearing comfort of such an orthosis and its effect on postoperative outcome in a comparison with customary plaster cast immobilization.

We thus decided to conduct a prospective, randomized cross-over study to examine these issues. The primary endpoint was patient satisfaction based on the 7 parameters of aesthetics, handling, sense of hygiene, activity limitations, physical resilience, accuracy of fitting and postoperative pain with the particular form of immobilization. The secondary endpoints were mobility of the wrist and finger function as determined by the range of motion (ROM) and the DASH Score, the period of time during which the patient was disabled and the radiological outcome.

## Materials and Methods

The protocol for this trial and supporting CONSORT checklist are available as supporting information; see S1 and S2 Protocol, S1 CONSORT Checklist and S1 Raw Data.

This study was approved by the ethics committee of Tuebingen University under the No. 196/2011 B02 and conducted in conformity with the principles of good clinical practice. The study was performed at the BG Trauma center Tuebingen.

Inclusion criteria were:

- age 18 to 80 years
- isolated, displaced, distal radius fracture type AO 23 A2, A3, B1, B2, B3, C1,C2
- surgical therapy with volar locking plate

Exclusion criteria were:

- open fracture
- non-displaced fracture
- blunt trauma of the soft tissues G2, G3
- associated fracture of the ulna (except Processus styloideus ulna fracture)
- pathological fracture
- patient unable to comply with postoperative therapy
- other concomitant fractures, polytrauma
- bilateral fractures

Between August 2011 and August 2012 all patients meeting the above-mentioned inclusion criteria were asked to participate in the study.

**Fig 1:**
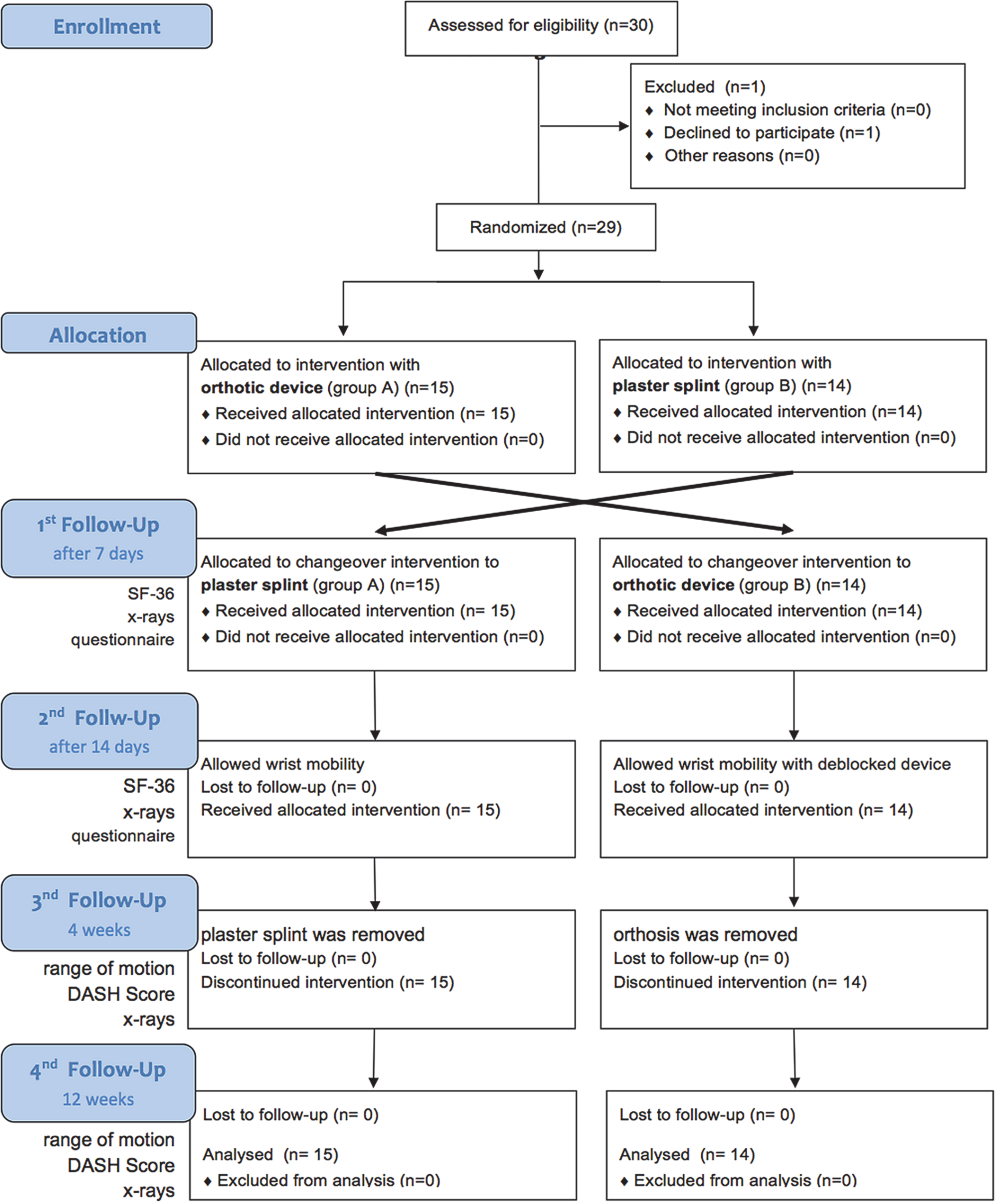
CONSORT 2010 Flow Diagram.

Thirty patients consented to participate (Fig. 1).

**Fig 2:**
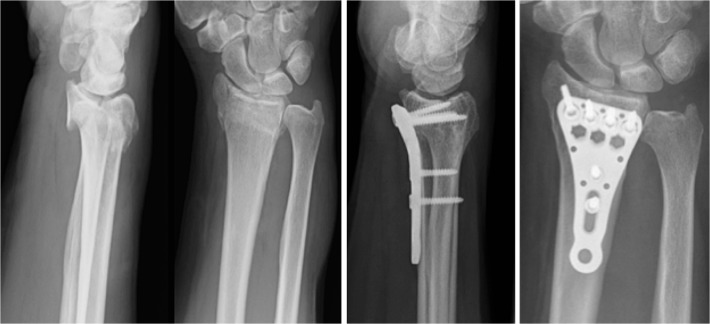
Example of AO 23 C2 fracture lateral and ap (anterior-posterior) view preoperative and 15 weeks postoperative x-rays stabilized with Königsee locking plate.

**Fig 3:**
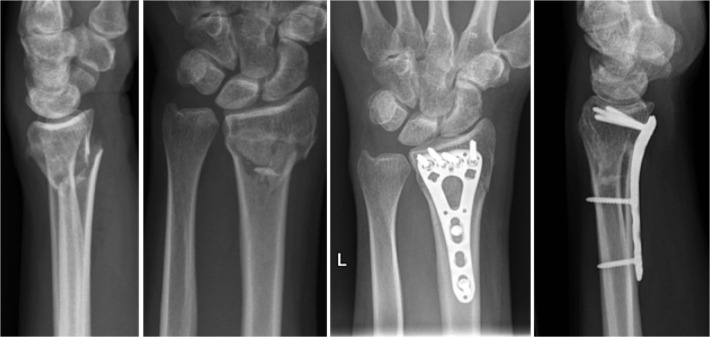
Example of AO 23 A3 fracture lateral and ap view preoperative and 14 weeks postoperative stabilized with Depuy/Synthes locking plate.

**Fig 4:**
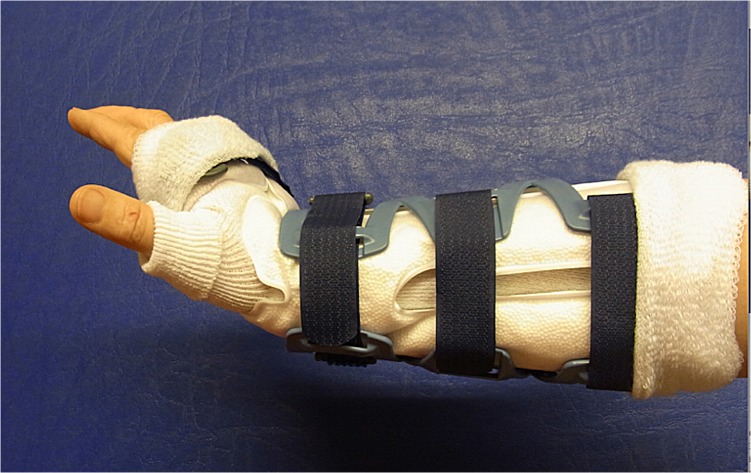
Vacuum fitted flexible orthosis (VacoHand OPED) deblocked in dorsal extension.

**Fig 5:**
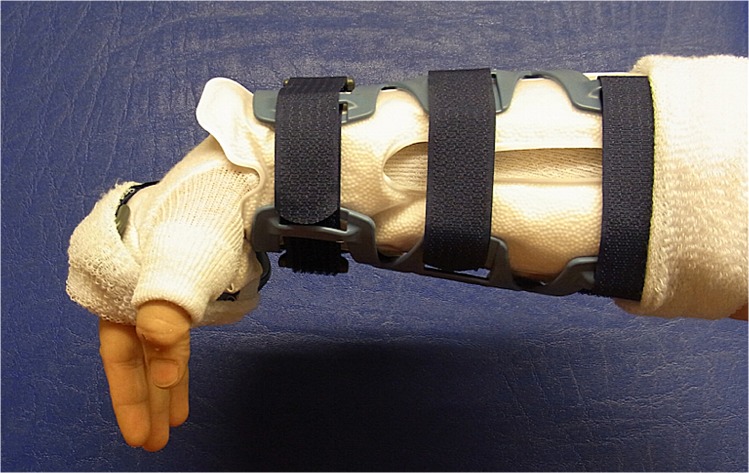
VacoHand Orthosis deblocked in palmar flexion.

**Fig 6:**
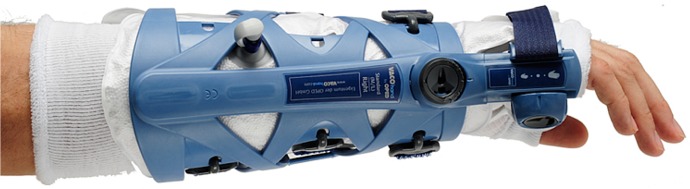
VacoHand Orthosis view from ulnar with visible hinge, blocked position 0°, during the study we used 20° dorsal extension as blocked position.

**Fig 7:**
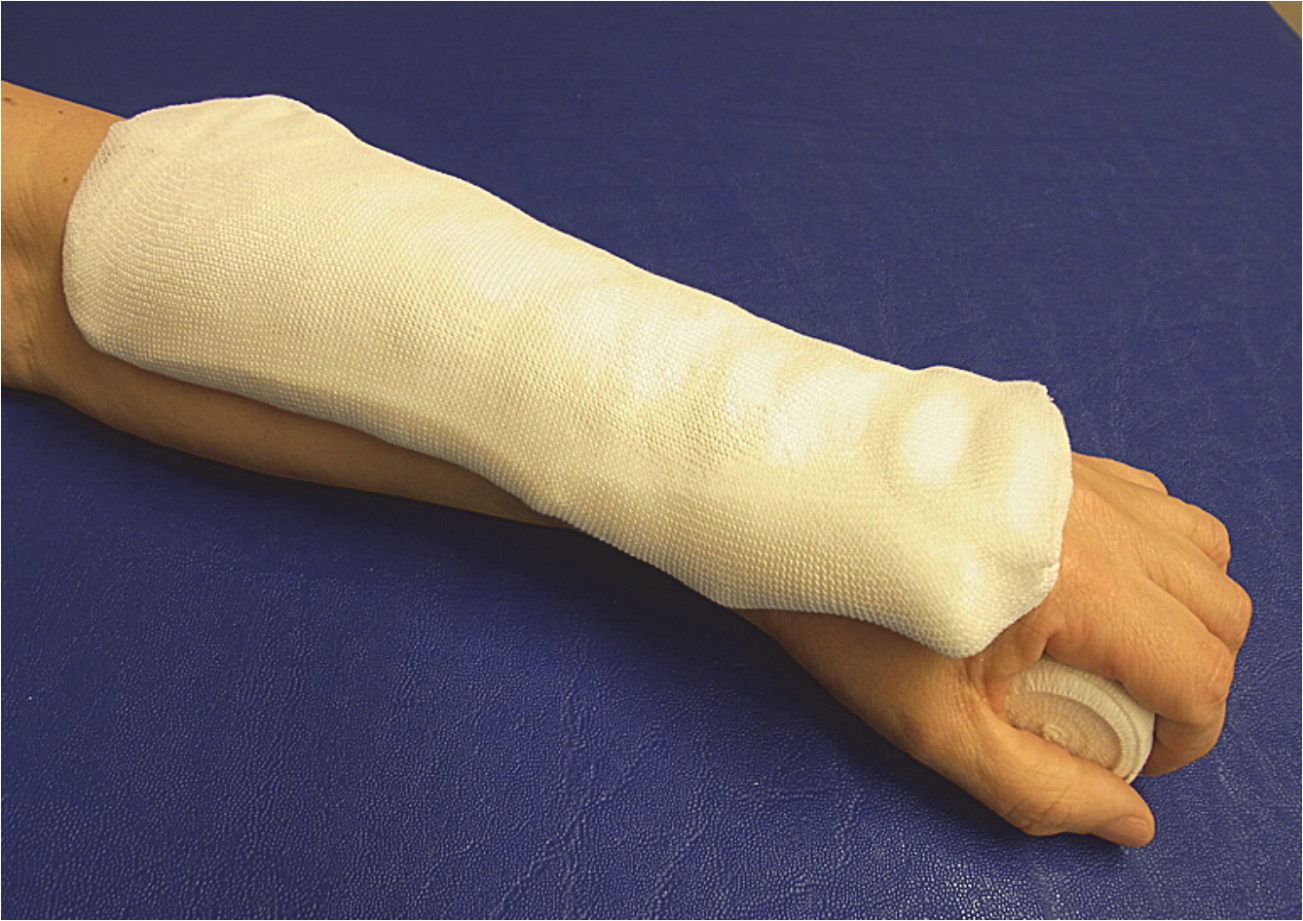
Forearm plaster splint (plaster of Paris) not attached.

All patients first underwent closed reduction with extension and application of semicircumferential splint. Following preoperative preparation and planning ostheosynthesis was performed with a volar, distal radius locking plate (either Königsee, 3.5mm (Fig. 2) or Synthes LCP 3.5mm (Fig. 3)). Postoperative immobilization was determined in advance with random assignment to a VacoHand orthosis (Figs. 4, 5, 6) or a dorsal forearm plaster splint (plaster of Paris) (Fig. 7).

The randomization was performed by labeling 30 participating certificates before the trial started. 15 were marked with „plaster splint“ and 15 were marked with „VacoHand“. The sheets were folded to conceal the writing and subsequently blended. Afterwards they were randomly inserted into an envelope which was sealed. The envelopes were blended once again and thereafter they were consecutively numbered with number 1–30. Each sealed envelope was filed into a folder together with the hand out of information material each patient received. The patients who agreed to participate received this folder and the envelope was opened. „Vacohand“ indicating the patient to be randomized into group A, „plaster splint“ allocated the patient to group B. A control for age or other factors except the described inclusion criteria was not performed beforehand.

The VacoHand, as from now on named orthosis, was designed by OPED and is a vacuum fitted orthosis with a wrist hinge which can be used either in a blocked position or in an unblocked, flexible mode allowing several degrees of range of motion.

Independently of the randomization, each of the devices was applied in the operating theatre by the surgical team. Irrespective of the method used, all patients initially underwent total postoperative immobilization of the wrist. After seven days at the first postoperative check-up (time-point 1) the immobilizing device was removed and the complementary orthotic device applied, as foreseen by the cross-over study design. The "new" device ensured immobilization for another seven days in both groups. The patients underwent a second check-up 14 days postoperative (time-point 2). From this time on, the patients were allowed to exercise wrist mobility out of the splint under the supervision of a physical therapist in case of the plaster of Paris group. The group treated with the orthosis which was deblocked at time-point 2 was allowed to exercise limited wrist movement in the orthosis at all times themselves in addition to the physiotherapy sessions. At the third follow-up examination four weeks postoperative (time-point 3) the plaster splint or orthosis was removed. The final examination was conducted at 12 weeks postoperative (time-point 4). The study design is also shown in the flow chart for a better understanding (Fig. 1).

At the first and second postoperative check-ups the patients were surveyed concerning their physical health using the SF-36 questionnaire and an additional individual questionnaire. At the third and fourth postoperative check-ups wrist mobility was determined according to range of motion and DASH Score.

The Short Form 36 (SF 36) is a validated health survey questionnaire to compare clinical outcomes of certain patient groups with normative data [20].

The DASH score (**D**isabilitys oft he **A**rm, **S**houlder and **H**and) produces values between 0 and 100 for each module, in which a high DASH score indicates severe disability [21].

The additional questionnaire used to assess postoperative pain, limitations of activity, physical resilience, handling of orthosis, sense of hygiene and accuracy of fitting was designed individually for this study. It contained 5 graduations for each oft he above mentioned items / questions reaching from 1 = absolutely correct, 2 = mainly correct, 3 = I do not know, 4 = mainly wrong, 5 = absolutely wrong. The results of these 7 parameters were subsumed as patient satisfaction.

At all four postoperative check-ups the patient underwent a clinical examination and x-ray examination of the wrist in anterior-posterior (ap) and lateral projections.

### Statistical Analysis

Demographical data, DASH-Scores and SF-36-Scores were presented as means with standard deviation, ROM as medians and interquartile range. Comparison between different demographical groups were made using the Fisher’s Exact Test for counting data and the (Welch) t-test for continuous variables [22]. The Wilcoxon Rank Sum Test was used for between group comparison of clinical outcome with SF36, DASH Score, and ROM. To match the subjective values of the questionnaire the Cochran–Armitage–Trend-Test was used for between group comparison and for within group time effects the Wilcoxon Signed Rank Test was used. A 2-sample test for equality of proportions was applied on the variables Right Handed and Fracture occurred on dominant side.

Statistical analysis was performed with R (R Core Team, 2013) (Version 3.0) in addition with R packages coin (Version 1.0.21) and HH (Version 2.3-37). Statistical results with a p-value < 0.05 were considered as statistically significant.

### Registry of the study

The study has been registered at the German Clinical Trials Register (DRKS) with the registration number: DRKS00006097.

Because of a change of the principal investigator there was a delay in registering this study (after enrolment of participants started). The authors confirm that all ongoing and related trials for this intervention are registered.

A written consent was obtained from all participants involved in this study.

## Results

In a 1-year period (August 15^th^ 2011 until August 30^th^ 2012) we recruited 30 patients with distal radius fractures, one of these was not available for follow up examination (Fig. 1). Therefore 29 patients (21 female, 8 male) took part in the study. Overall mean age was 48,28 (20–75 years) SD ±15,85, of these the female patients had a mean age of 50,14 SD ±15,3 years while the male patients were younger with a mean age of 43,38 SD ±17,5 years (Table 1).

**Table 1:** Demographic Data.

	Overall	Group A	Group B	P-Value
**N**	29 (100%)	15 (52%)	14 (48%)	-
Male	8 (28%)	5 (33%)	3 (21%)	0.682
Female	21 (72%)	10 (67%)	11 (79%)	
**Age (years)**	48.28±15,85	50.93±15.18	45.43±16.61	0.361
Male	43.38±17.50	48.00±16.78	35.67±19.14	0.499
Female	50.14±15.30	52.40±15.04	48.09±15.78	0.529
**Right Handed**	24 (83%)	11 (73%)	13 (93%)	0.369
**Fracture occurred on dominant side**	15 (52%)	8 (53%)	7 (50%)	1.000
**Fracture classification**				
A	14 (48%)	7 (47%)	7 (50%)	1.000
B	3 (10%)	2 (13%)	1 (7%)	
C	12 (42%)	6 (40%)	6 (43%)	
**Decision VacoHand**				
Yes	23 (79%)	9 (60%)	14 (100%)	
No	6 (21%)	6 (40%)	0 (0%)	0.017

15 patients were randomly allocated to group A (first week with orthosis, Fig. 4) 10 of these being female and 5 male with a mean age 50,93 SD ±15,18 years.

14 patients were randomly allocated to group B (first week with dorsal forearm plaster splint, Fig. 7), 11 of these were females and 3 male with a mean age 45,43 SD ±16,61 years.

In group A 11 of the 15 patients and in group B 13 of 14 patients were right handed (Table 1).

In group A the fracture occurred in 8 of 14 patients to the dominant side, one patient did not prefer one side as dominant. In group B the dominant side was affected in 7 of the 14 patients.

The fractures of group A were classified as 7 x AO type A, 2 x AO type B and 6 x AO type C, in group B we found 7 x AO type A, 1 x AO type B and 6 x AO Typ C.

There were no statistical differences in the distribution of the baseline characteristics.

The operative treatment in all cases was performed with open reduction and volar plating with anatomical locking plates (23 x Königsee, 6 x Depuy/Synthes), intraoperative or directly postoperative complications did not occur.

The postoperative pain was assessed with a questionnaire, concerning this we could not detect any significant differences between the groups at time-point 1 (p = 0,127) or time-point 2 (p = 0,820).

The subjective limitations of activity were higher in group B at time-point 1 though the difference was not significant (p = 0,075), at time-point 2 the differences were balanced (p = 0,251). Interestingly the change of activity limitations were significantly better (p = 0,003) in group B when the change of treatment from the plaster splint to the orthosis was performed compared to group A were the treatment was changed vice versa. The patients did not indicate any differences in physical resilience in either group (p = 0,360).

The handling of the immobilizing device was judged as significantly superior for the orthosis especially after the switch at time-point 2 with p = 0,009 in group A and p = 0,001 in group B.

The sense of hygiene was becoming significantly better in both groups when changing the treatment at time-point 1. In group B there was a more radical change concerning sense of hygiene when changing from plaster splint to orthosis (p = 0,001) which was not as distinctive in group A (p = 0,004)

Concerning accuracy of fitting (p = 0,025) and aesthetics (p = 0,001) the orthosis was estimated as significantly superior by the patients compared to the plaster splint. In addition there was a significant change in estimation of aesthetics when group B changed the orthotic device at time-point 1 (p = 0,015) (Table 2).

**Table 2:** Questionaire on subjective experience.

Item	Day Postoperative	Group	Absolutly Correct	Mainly Correct	I don’t know	Mainly Wrong	Absolutly Wrong	p-Value
aesthetic	Time-point 1	A	9 (60%)	4 (27%)	2 (13%)	0 (0%)	0 (0%)	< 0.001
		B	1 (7%)	2 (13%)	4 (27%)	6 (40%)	2 (13%)	
	Time-point 2	A	6 (40%)	6 (40%)	2 (13%)	1 (7%)	0 (0%)	0.719
		B	7 (47%)	3 (20%)	3 (20%)	2 (13%)	0 (0%)	
handling	Time-point 1	A	14 (93%)	0 (0%)	1 (7%)	0 (0%)	0 (0%)	< 0.001
		B	3 (20%)	1 (7%)	1 (7%)	5 (33%)	5 (33%)	
	14th	A	5 (33%)	6 (40%)	0 (0%)	3 (20%)	1 (7%)	0.001
		B	15 (100%)	0 (0%)	0 (0%)	0 (0%)	0 (0%)	
sense of hygiene	Time-point 1	A	11 (73%)	4 (27%)	0 (0%)	0 (0%)	0 (0%)	< 0.001
		B	2 (13%)	0 (0%)	0 (0%)	9 (60%)	4 (27%)	
	Time-point 2	A	2 (13%)	12 (80%)	0 (0%)	1 (7%)	0 (0%)	0.001
		B	11 (73%)	4 (27%)	0 (0%)	0 (0%)	0 (0%)	
activity limitations	Time-point 1	A	3 (20%)	6 (40%)	1 (7%)	3 (20%)	2 (13%)	0.363
		B	7 (47%)	3 (20%)	1 (7%)	3 (20%)	1 (7%)	
	Time-point 2	A	5 (33%)	3 (20%)	1 (7%)	3 (20%)	3 (20%)	0.206
		B	9 (60%)	2 (13%)	1 (7%)	1 (7%)	2 (13%)	
Physical resilience	Time-point 1	A	8 (53%)	4 (27%)	0 (0%)	1 (7%)	2 (13%)	0.075
		B	11 (73%)	4 (27%)	0 (0%)	0 (0%)	0 (0%)	
	Time-point 2	A	7 (47%)	4 (27%)	1 (7%)	0 (0%)	3 (20%)	0.251
		B	3 (20%)	6 (40%)	0 (0%)	2 (13%)	4 (27%)	
accuracy of fitting	Time-point 1	A	6 (40%)	8 (53%)	1 (7%)	0 (0%)	0 (0%)	0.642
		B	7 (47%)	5 (33%)	2 (13%)	1 (7%)	0 (0%)	
	Time-point 2	A	5 (33%)	5 (33%)	0 (0%)	5 (33%)	0 (0%)	0.254
		B	8 (53%)	7 (47%)	0 (0%)	0 (0%)	0 (0%)	
postoperative pain	Time-point 1	A	2 (13%)	4 (27%)	0 (0%)	4 (27%)	5 (33%)	0.127
		B	1 (7%)	10 (67%)	0 (0%)	2 (13%)	2 (13%)	
	Time-point 2	A	2 (13%)	3 (20%)	0 (0%)	4 (27%)	6 (40%)	0.820
		B	3 (20%)	3 (20%)	0 (0%)	2 (13%)	7 (47%)	

In the analysis of the subgroups we could not detect any influences of side dominance, age of patient or fracture type onto the rating concerning the handling, the sense of hygiene, the accuracy of fitting and aesthetical decision.

We could not detect any differences concerning activity limitations, physical resilience or postoperative pain between the two groups.

In the final evaluation performed with Fishers Exact Test for Count Data 23 of the 29 patients would prefer the orthosis in case they would suffer another fracture (p = 0,017) All 14 patients of group B and 9 patients of group A decided in favour of the orthosis.

**Fig 8:**
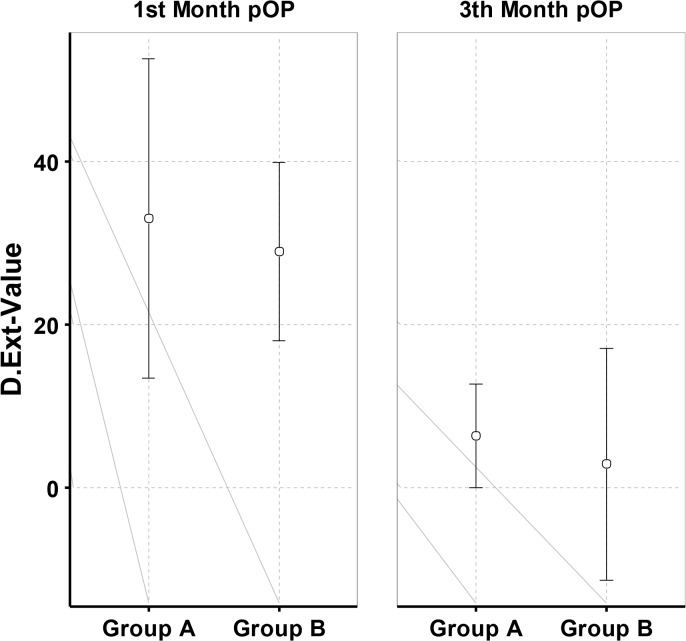
Mean and standard deviation of dorsal extension at time-point 3 and time-point 4 postoperative.

**Fig 9:**
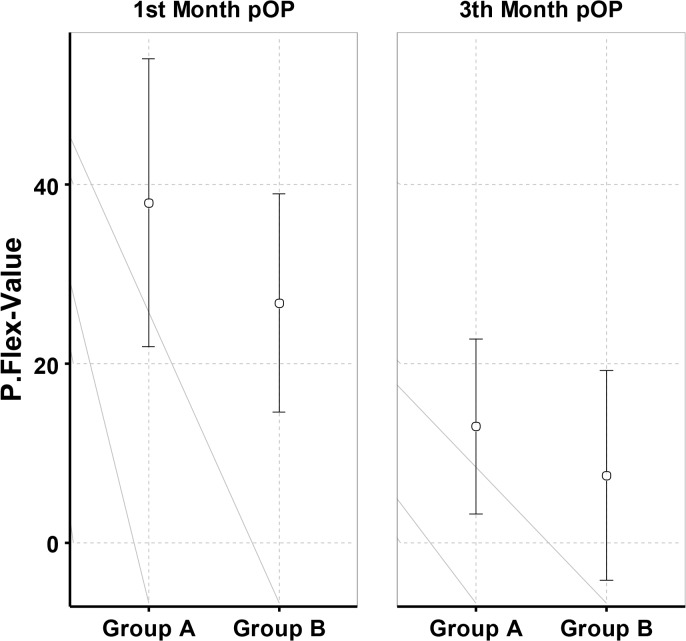
Mean and standard deviation of palmar flexion at time-point 3 and time-point 4 postoperative.

The clinical evaluation at time-point 3 (4 weeks) and 4 (12 weeks) comprised the investigation of ROM and radiological findings (Figs. 8 and 9).

We detected a significant difference between the two groups with a significantly (p = 0,044) reduced ability of palmar flexion at time-point 3 and in addition a reduced, yet not significant dorsal extension in the group which was treated with the plaster splint after week one and thus was only treated by physiotherapy out of the splint in the weeks 3 and 4. The patients treated with the orthosis which was used in a blocked, neutral position for the first two weeks and subsequently used unblocked in weeks 3 and 4 showed a significant advantage in functionality measured as ROM related to palmar flexion at time-point 3 and non significant palmar flexion and dorsal extension at time-point 4.

At time-point 4 one patient out of group A could not clench his fist completely, but we did not find a significant difference concerning the ROM of the wrist joint.

The recovery of ROM seemed to be faster in group B compared with group A (Table 1 and 2) though we could not find significant differences concerning this matter.

The radiological examinations at time-point 2, 3, and 4 did not show any secondary displacement or implant loosening. At time-point 4 (12 weeks) all fractures displayed signs of radiological consolidation. (Figs. 2 and 3)

The ROM of the forearm concerning pronation and supination and the ROM of the elbow joint was comparable in both groups.

We also tried to find out about time of disability to work which was 45.8 days in group A and 39.6 days in group B, but due to different types of professions (pensioners, students, dependent employee, and freelancer) and missing values it made no sense to perform a statistical calculation.

**Fig 10:**
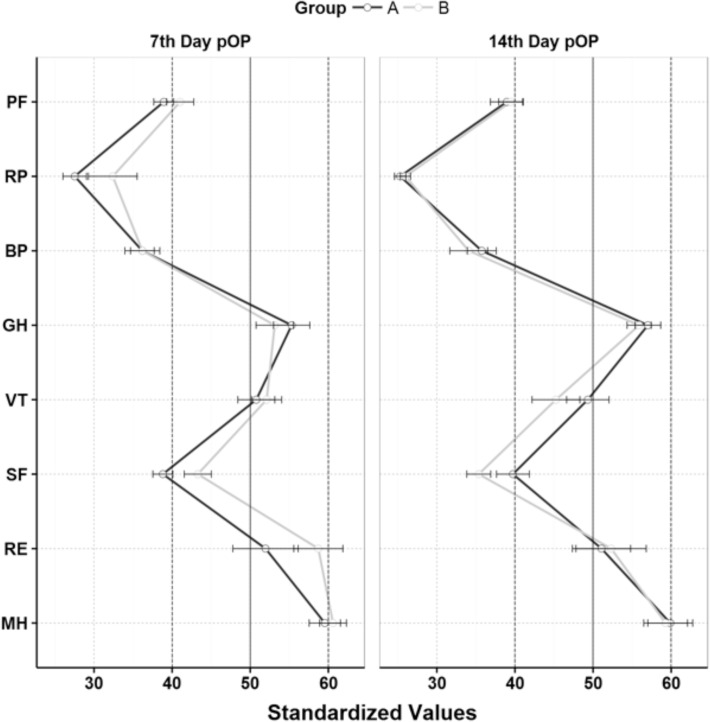
Mean and standarddeviation of the 8 SC-36 Scales. SF-36 Measurement Model: Physical Health: Physical Functioning (PF), Role-Physical (RP), Bodily Pain (BP), and General Health (GH); Mental Health: Vitality (VT), Social Functioning (SF), Role-Emotional (RE), and Mental Health (MH). Scales of our sample were standardized on German normative postoperativ evaluation [21]. The normative German postoperative evaluation has mean 50 and standarddeviation 10.

The SF36 did not show any significant differences between the two groups (Fig. 10).

**Fig 11:**
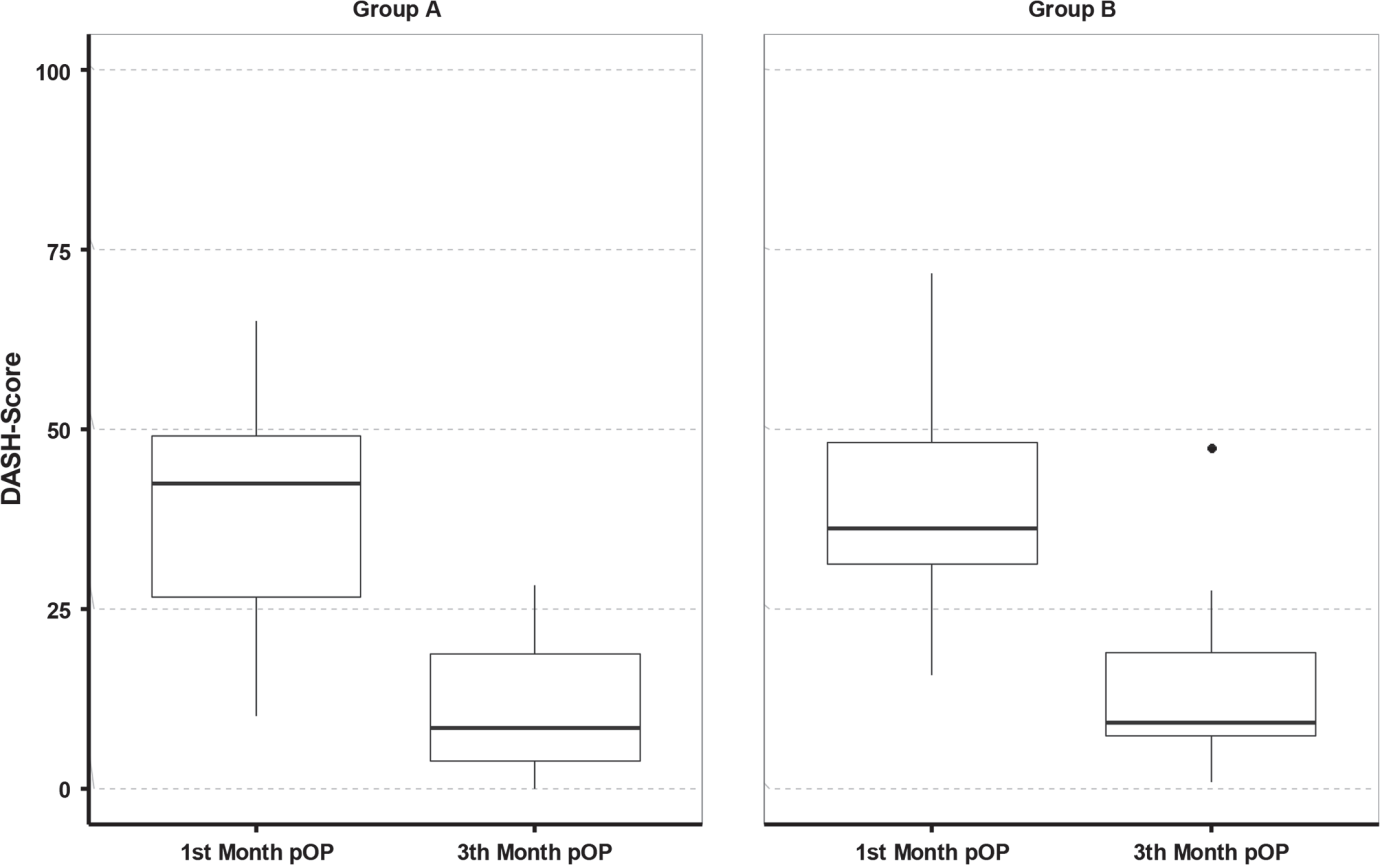
DASH Score at time-point 3 and time-point 4.

The mean DASH score of all patients at time-point 3 was 38,53 ±15,69 and at time-point 4 it was 12,82 ±10,86. Significant differences between the groups could not be detected (Fig. 11).

As complications of our treatment we identified one patient with a CRPS out of group B and one patient with slight paraesthesia in group A.

## Discussion

To date the scientific literature contains no prospective comparative study of patient satisfaction and functional outcome with various orthosis for postoperative treatment. We only found a meta-analysis of 8 studies comparing dynamic versus static fixation of distal radius fractures with an external fixateur device with respect to complications and malunion [23], reporting of superior but non-significant range of motion and better radiological outcome in the dynamic group [24–31]. Some investigators retrospectively compared dynamic splinting after surgically treated distal radius fractures versus nonsurgical treatment coming to the conclusion, that dynamic splinting is directly responsible for gains in ROM [32]. With regard to treatment of contractures after distal radius fractures one study did not find a reduction in the rate of contracture by using a dynamic orthosis and thus concluded them to be difficult to justify for this diagnosis [33].

For therapy of fractures of the upper extremity, the plaster cast or synthetic casts are still assumed to be the therapy most frequently used. Although there seem to be advantages of the synthetic materials [34] the plaster cast is still preferred in a lot of hospitals.

Despite the availability of a flexible vacuum-fitted orthosis for the upper extremity it is so far rarely used in hospitals. The reason for this might be the absence of evidence of effects and results of a flexible orthosis in case of postoperative treatment of forearm fractures.

The main focus of this study was patient satisfaction with regard to the two different devices used for immobilization, which was investigated using the described questionnaires. These questionnaires only comprised of 5 steps and were self designed. Retrospectively we should have used a scale from 1 to 10 for each item to receive a more detailed graduation and a better reproducibility.

The study design does not include a wash-out period and therefore does not totally correlate to the design of a standard cross-over trial. Thus we could not completely avoid carry–over effects, but due to the injury related and therefore essential therapy we were not able to fulfill all the requirements.

The study design did not include a one year follow up, because we did not expect a significant difference concerning the function postoperatively and because the main target point of the study was patient satisfaction and comfort. Therefore we are not able to judge how the differences in range of motion which already seemed to have leveled after 12 weeks would perform after a one year period.

For the answers concerning patient satisfaction with the device this design was sufficient. For the evaluation of the questions concerning final ROM and radiological results another examination after 12 month would have been reasonable, but as mentioned before these were only secondary target points in this study.

The therapy regimen for postsurgical treatment of a distal radius fracture employed in our Level I Trauma Center and University Clinic calls for two weeks of immobilization after operative stabilization followed by two weeks of mobilization in the splint under attendance of a physiotherapist. The splint is removed after four weeks and light mobility is prescribed for the subsequent two weeks without strain.

The study was conducted according to this therapy regimen and the study design adapted to ensure fair comparison of the two treatment methods, because the deblocked and thus flexible orthosis would permit mobilization after two weeks.

We purposely did not add a third group without any postoperative immobilization as this would have contradicted our well-established treatment regimen and we believe that this might lead to secondary displacement or implant loosening especially in patients with reduced bone quality.

Of course in other hospitals the postoperative treatment of distal radius fractures is handled differently [9,14,16], sometimes even with no immobilization at all [15]. With increasing age of our future patients and thus reduced bone quality the confidence into postoperative stability might decrease and therefore postoperative immobilization to safeguard the operative result might be used more often in future even by those not doing it presently.

Using the SF 36 questionnaire and the DASH Score and several individually developed questionnaires we detected no significant differences concerning pain, overall functional ability and resilience. In relation to change of activity limitations when changing the treatment after week 1 the group changing from orthosis to plaster splint reported a significantly worse result compared to the other group. Also we found a significantly superior evaluation of accuracy of fitting, aesthetics, and hygienic feeling in favour of the orthosis. The main question and target point of the study, namely which treatment (orthosis or plaster splint) would be preferred in future after this cross-over study, was clearly and thus significantly (p = 0,017) answered in favour of the orthosis. Of the 29 study participants, 23 would request the vacuum fitted orthosis as postoperative fracture immobilization in case of another distal radius fracture.

As the orthosis was deblocked after the second week the patients were able to perform some limited movements themselves at all times in addition to the physiotherapy treatment both groups received. We assume that this additional exercises accounts for the superior functional outcome compared to the plaster splint group after 4 weeks and the better, yet not significant ROM after 12 weeks.

From a financial point of view, the flexible orthosis is more economical for hospitals in Germany at this time, because it calls for neither material nor personnel to apply or remove it as the plaster splint does. Currently, the social health insurance carriers and not the hospital itself pay for the flexible orthosis. One negative point is certainly the fact that this is thus an added expense for German social health insurance carriers, whereby a comparison of costs for the provision of a plaster room, personnel and material will not be entirely transparent and thus not comparable. The costs of the plaster splint for example are taken over by the hospital itself.

The study demonstrates that there are some advantages in functional outcome after 4 weeks and in postoperative management when using a functional orthosis, and that patient satisfaction clearly is superior with the vacuum-fitted flexible orthosis we used in this study. After twelve weeks the functional advantages seem to have almost leveled. Consequently, the study data suggests the flexible orthosis to be an alternative to the plaster splint if postoperative immobilization is wanted.

All data underlying the findings described in this manuscript are fully available without restriction. They were submitted with the manuscript and are available via PLOSONE.

## Supporting Information

S1 CONSORT Checklist (DOC)

S1 Protocol Trial study protocol (German). (DOC)

S2 Protocol Trial study protocol (English). (DOCX)

S1 Raw Data Raw data and statistical analysis. (PDF)
