# Supplementary material for: Early Functional Postoperative Therapy of Distal Radius Fracture with a Dynamic Orthosis: Results of a Prospective Randomized Cross-Over Comparative Study
Source: PLoS One. 2015 Mar 30;10(3):e0117720. doi: 10.1371/journal.pone.0117720 (PMC4378993; doi:10.1371/journal.pone.0117720)
Supplement: S1 Protocol — (DOC) [file pone.0117720.s002.doc]

**Version 1.0**

**Prüfplan**

**zur Studie**

**Outcomeanalyse bei der Therapie einer Radiusfraktur mit**

**VacoHand**®**/ Unterarmgipsschiene**

**Prospektive klinische cross-over Studie**

| **Kurztitel der klinischen Studie:** | **VACUM** |
| --- | --- |
|  |  |
| **Version:** | **1** |
|  |  |
| **Datum des Prüfplans:** | **01.03.2011** |
|  |  |
| **Leiter der Studie:** | **Dr.med. Fabian Stuby** |

Inhaltsverzeichnis

*1* *Allgemeine Information* [*4*](#__RefHeading___Toc293583694)

1.1 Verantwortliche und Unterschriften: [4](#__RefHeading___Toc293583695)

1.2 Adressen [5](#__RefHeading___Toc293583696)

1.3 Prüfplanzusammenfassung (Synopsis) [6](#__RefHeading___Toc293583697)

2 Einleitung [7](#__RefHeading___Toc293583698)

2.1 Zusammenfassung [7](#__RefHeading___Toc293583699)

3 Ziele der Studie [8](#__RefHeading___Toc293583700)

4 Studiendesign [8](#__RefHeading___Toc293583701)

4.1 Design [8](#__RefHeading___Toc293583702)

4.2 Studiendauer [8](#__RefHeading___Toc293583703)

5 Studienpopulation [8](#__RefHeading___Toc293583704)

5.1 Rekrutierung [8](#__RefHeading___Toc293583705)

5.2 Einschlusskriterien [8](#__RefHeading___Toc293583706)

5.3 Ausschlusskriterien [10](#__RefHeading___Toc293583707)

5.4 Aufnahme in die Studie [10](#__RefHeading___Toc293583708)

6 Studienablauf und Untersuchungsmethoden [10](#__RefHeading___Toc293583709)

6.1 Klinische Untersuchungen [10](#__RefHeading___Toc293583710)

6.2 Studienbedingte Probeentnahmen [10](#__RefHeading___Toc293583711)

6.3 Geplante Untersuchungen [10](#__RefHeading___Toc293583712)

6.4 Durchführung und Beendigung [10](#__RefHeading___Toc293583713)

6.5 Datenschutz [11](#__RefHeading___Toc293583714)

6.6 Ethische Belange [12](#__RefHeading___Toc293583715)

6.7 Aufklärung der Studienteilnehmer [12](#__RefHeading___Toc293583716)

7 Methoden zur Erfassung der Wirksamkeit und Sicherheit [12](#__RefHeading___Toc293583717)

7.1 Risiken und Nebenwirkungen [12](#__RefHeading___Toc293583718)

8 Angaben zur Statistik, Auswertung [12](#__RefHeading___Toc293583719)

9 Datenmanagement [12](#__RefHeading___Toc293583720)

10 Ethische Belange, Versicherung [12](#__RefHeading___Toc293583721)

10.1 Deklaration von Helsinki [12](#__RefHeading___Toc293583722)

10.2 Versicherung [13](#__RefHeading___Toc293583723)

11 Allgemeine Bestimmungen, Vereinbarungen, organisatorische Abläufe [13](#__RefHeading___Toc293583724)

11.1 Angaben zur Finanzierung [13](#__RefHeading___Toc293583725)

11.2 Publikationen [13](#__RefHeading___Toc293583726)

Anhang: [13](#__RefHeading___Toc293583727)

Anlage 1: Perimed-Bogen [13](#__RefHeading___Toc293583728)

Anlage 2: SF-36 Fragebogen [13](#__RefHeading___Toc293583729)

Anlage 3: Einverständniserklärung [13](#__RefHeading___Toc293583730)

# Allgemeine Information

## 1.1 Verantwortliche und Unterschriften:

Dr.med. Fabian Stuby

Datum Name Unterschrift

Dr. med. Atesch Ateschrang

Datum Name Unterschrift

Dr. med. D. Zieker

Datum Name Unterschrift

## 1.2 Adressen

**Leiter und Autoren der prospektiven klinischen cross-over Studie**

| 1. Dr. med. Fabian Stuby  Unfall- und Wiederherstellungschirurgie, Berufsgenossenschaftliche Unfallklinik Tübingen  Schnarrenbergstr. 95, 72076 Tübingen  Tel.: ++49 7071 – 60 60  Email: fstuby@bgu-tuebingen.de  2. Dr. med. Atesch Ateschrang  Unfall- und Wiederherstellungschirurgie, Berufsgenossenschaftliche Unfallklinik Tübingen  Schnarrenbergstr. 95, 72076 Tübingen  Tel.: ++49 7071 – 60 60  Email: aateschrang@bgu-tuebingen.de  3. Dr. med. Derek Zieker  Universitätsklinik für Allgemeine, Viszeral- und Transplantationschirurgie  Hoppe-Seyler-Str. 3, 72076 Tübingen  Tel.: ++49 7071 – 29 85073  Email: derek.zieker@med.uni-tuebingen.de |
| --- |

Zuständige Ethik-Kommission für den LKP

| Vorsitzender: Prof. Dr. Dieter Luft  Geschäftsführende wiss. Angestellte: Dr. Olga Scheck  Sekretariat: Karin Zähres, Anett Rönnfeld  Universitätsklinikum Tübingen, Schleichstr. 8, 72076 Tübingen  Tel.: 07071/297 7661  Fax: 07071/29 5965  Email: [ethik.kommission@med.uni-tuebingen.de](mailto:ethik.kommission@med.uni-tuebingen.de) |
| --- |

## 1.3 Prüfplanzusammenfassung (Synopsis)

| Studiencode | VACUM | |
| --- | --- | --- |
| Titel der Studie | Outcomeanalyse bei der Therapie einer Radiusfraktur mit  VacoHand®/ Unterarmgipsschiene | |
| Studiendesign | Prospektive klinische cross-over Studie | |
| Geplante Patientenzahl | 50 Patienten | |
| Studiendauer | Geplanter Studienbeginn:  Geplante Studiengesamtdauer: | 5/2011  18 Monate |
| Primäres Zielkriterium | Das primäre Studienziel ist Patientenzufriedenheit gemessen am SF-36 Fragebogen. Sekundäre Studienziele sind die Ermittlung des DASH-Scores und der Neutral-0-Methode für die Hand, sowie die Dauer der Arbeitsunfähigkeit. | |
| Einschlußkriterien | 18 Jahre und operationspflichtige AO2.3 A2, A3, B1, B2, B3, C1, C2 Frakturen, mit einer volaren winkelstabilen Platte (2,4mm, 3,5mm, alle Typen) | |
| Ausschlusskriterien | - Unter 18 Jahre - Alle anderen AO 2.3 Frakturen - Offene Frakturen und Begleitverletzungen - Unterarmfrakturen (bis auf Proc. Styloideus ulnae) - Pathologische Frakturen - Patienten, die nicht in der Lage sind die postoperative Behandlung zu unterstützen | |

# 2 Einleitung

## 2.1 Zusammenfassung

Die distale Radiusfraktur gehört zu den häufigsten unfallchirurgischen Krankheitsbildern und tritt mit einer Inzidenz von zwei bis drei auf 1000 Einwohner im Jahr auf. Es handelt sich um einen handgelenksnahen (distalen) Bruch der Speiche (Radius) und wird daher im Volksmund als Handgelenksbruch bezeichnet. Er tritt in allen Altersgruppen auf, gehäuft aber zwischen dem 6. und 18. Lebensjahr sowie zwischen dem 60. und 70. Lebensjahr. Der Entstehungsmechanismus geht in der Regel von einem traumatischen Sturz auf das überstreckte (Colles-Fraktur), seltener auf das gebeugte Handgelenk (Smith-Fraktur) aus. Während die distale Radiusfraktur bei Kindern und Jugendlichen vor allem beim Sport auftritt, kommt es beim älteren Menschen meistens aufgrund eines Stolpersturzes auf unebenen oder glatten Untergrund zur Fraktur. Ziel jeglicher Therapie sei es konservativ oder operativ ist die anatomische Wiederherstellung der Gelenkfläche. Instabile extraartikuläre und intraartikuläre Frakturen werden meist mittels Plattenosteosynthesen stabilisiert. Diese werden meist beugeseitig, selten auch streckseitig auf der handgelenksnahen Speiche angebracht. Der Vorteil der Verplattung liegt in der sofortigen Übungsstabilität, so dass nach der postoperativen Gipsanlage mit der frühzeitigen Physiotherapie begonnen werden kann. Eine frühzeitige Mobilisierung kann den negativen Folgen einer Gelenkimmobilisation, wie Muskelatrophien, Sehnenverklebung und Kapselschrumpfung vorbeugen. Mehrere Studien haben gezeigt, dass die Ruhigstellung im Gips zu einer deutlichen Atrophie der Muskulatur führt, vor allem in den ersten zwei Wochen. Zusätzlich kommt es zu metabolischen, neurologischen und funktionellen Anpassungserscheinungen, so dass sich die Muskelkraft nach 4–6 wöchiger Gipsruhigstellung um fast die Hälfte verringern kann. Eine mögliche Lösung des Problems könnte in der Anwendung einer dynamischen Vakuumorthese liegen, die sowohl die Stabilität eines zirkulären Unterarmgipses bietet und somit eine frühzeitige risikoarme Belastungssteigerung erlaubt, als auch eine limitierte Beweglichkeit im Handgelenk ermöglicht. Außerdem erhöht sich der Patientenkomfort dadurch, dass eine Orthese weniger wiegt als ein Gips und nach entsprechender Einweisung auch vom Patienten einfach anzulegen ist und eine regelmäßige Körperpflege ermöglicht wird. Daneben kann durch die limitierte Freigabe der Beweglichkeit ein physiologischeres Funktionsbild des Handgelenks erzielt werden. Des Weiteren könnte eine günstig verlaufende postoperative Phase bei der berufstätigen Bevölkerung zu einer früheren Wiederaufnahme der Tätigkeit führen.

# 3 Ziele der Studie

Das primäre Ziel des Vorhabens ist die Patientenzufriedenheit gemessen am SF-36 Fragebogen nach operationspflichtigen AO2.3 A2, A3, B1, B2, B3, C1, C2 Frakturen, mit einer volaren winkelstabilen Platte (2,4mm, 3,5mm, alle Typen), die postoperativ mit einer VacoHand®/ Unterarmgipsschiene versorgt wurden. Es werden gezielte Befragungen bezüglich des Schmerzes und der Lebensqualität der Patienten erfolgen. Anhand dieser Daten werden die beiden postoperativen Therpaiemethoden in Vergleich gesetzt. Sekundäre Studienziele sind die Ermittlung des DASH-Scores und der Neutral-0-Methode für die Hand, sowie die Dauer der Arbeitsunfähigkeit. Dazu sollen 1, 2, 6 Wochen und 6 Monate nach der postoperativen Versorgung Nachuntersuchungen erfolgen.

# Studiendesign

## 4.1 Design

Es handelt sich um eine prospektiv klinische cross-over Studie

.

## 4.2 Studiendauer

Die Studiendauer inklusive Datenerhebung sollte 18 Monate nicht überschreiten.

# Studienpopulation

## 5.1 Rekrutierung

Die Rekrutierung der Patienten erfolgt in der Unfall- und Wiederherstellungschirurgie, Berufsgenossenschaftliche Unfallklinik Tübingen. Die Patienten werden über die Studie informiert, erhalten das Informationsblatt und ein Beratungsgespräch, in dem alle offenen Fragen geklärt werden.

## 5.2 Einschlusskriterien

- >18 Jahre
- Operationspflichtige AO2.3 A2, A3, B1, B2, B3, C1, C2 Frakturen, die mit einer volaren winkelstabilen Platte (2,4mm, 3,5mm, alle Typen) versorgt werden
- Weichteile G0, G1
- Patienten müssen in der Lage sein die postoperative Behandlung aktiv zu unterstützen.

**AO-Einteilung der distalen Radiusfraktur:**


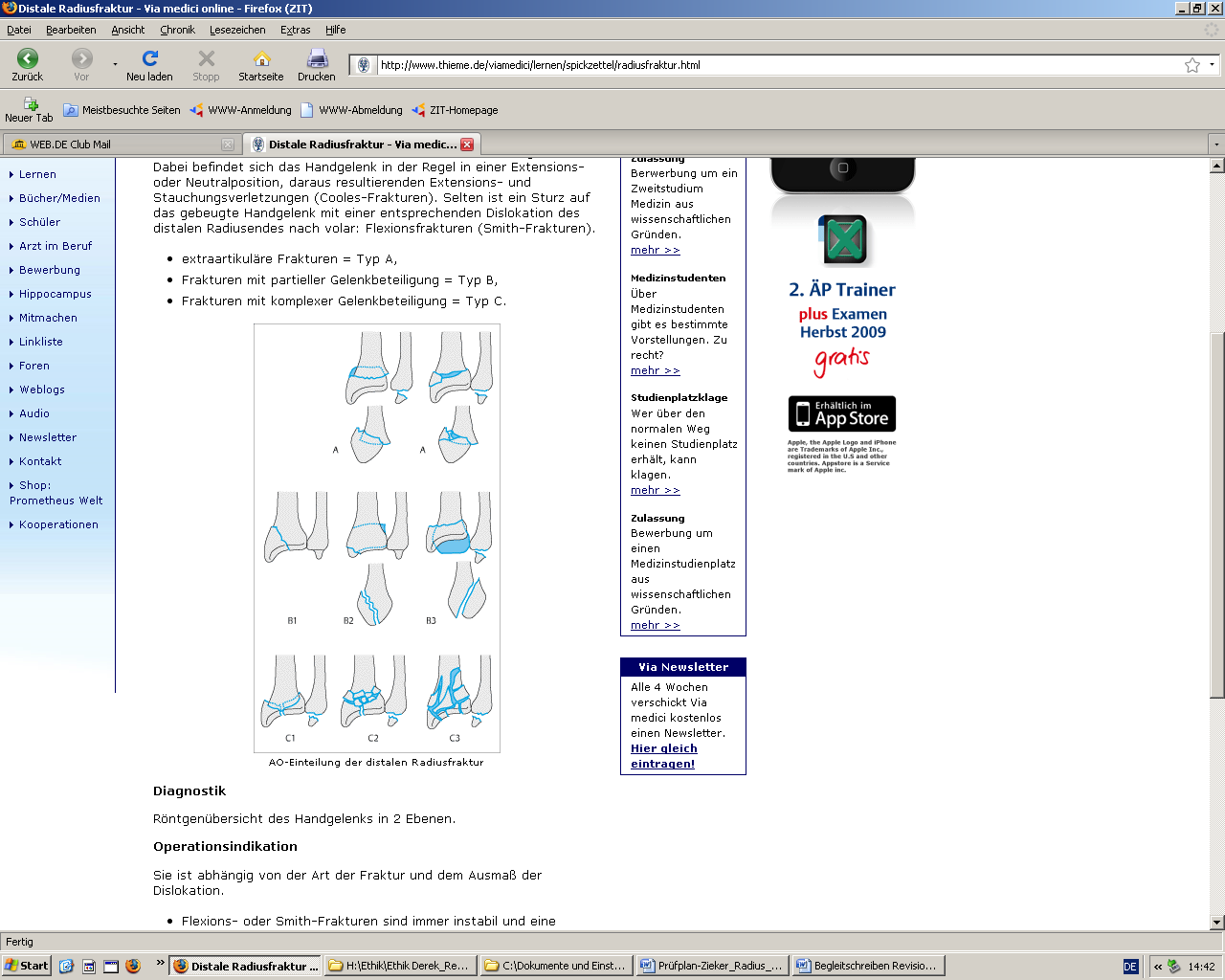


Kopiert aus Thieme „via medici“

#### **Geschlossene Frakturen (Weichteilklassifikation):**

- Grad 0: Keine oder unbedeutende Weichteilverletzung, indirekte Gewalteinwirkung, einfache Frakturform
- Grad I: Oberflächliche Hautabschürfung oder Quetschung (Kontusion) durch Fragmentdruck von innen, einfache bis mittelschwere Frakturform
- Grad II: Tiefe, verschmutzte Hautabschürfung, Kontusion durch direkte Gewalteinwirkung, drohendes Kompartmentsyndrom, mittelschwere bis schwere Frakturform
- Grad III: Ausgedehnte Hautkontusion oder Zerstörung der Muskulatur, subkutanes Decollement, manifestes Kompartmentsyndrom, Verletzung eines Hauptgefäßes

## 5.3 Ausschlusskriterien

- <18 Jahre
- Alle anderen AO 2.3 Frakturen
- Weichteile G2, G3
- Offene Frakturen und Begleitverletzungen
- Unterarmfrakturen (bis auf Proc. Styloideus ulnae)
- Pathologische Frakturen
- Patienten, die nicht in der Lage sind die postoperative Behandlung zu unterstützen

## 5.4 Aufnahme in die Studie

Alle Patienten, die die Einschlußkriterien erfüllen, und keine Ausschlusskriterien vorweisen, werden in die Studie aufgenommen (Vgl. Rekrutierung, 5.1).

# Studienablauf und Untersuchungsmethoden

## 6.1 Klinische Untersuchungen

Klinische Untersuchungen werden zur Diagnosesicherung, Kontrolle und zur Evaluation der Patientenzufriedenheit und Schmerzentwicklung durchgeführt.

## 6.2 Studienbedingte Probeentnahmen

entfällt.

## 6.3 Geplante Untersuchungen

Es sind regelmäßige klinische Nachuntersuchungen und Kontrollen, sowie gezielte Befragungen bezüglich Schmerz und Patientenzufriedenheit der Patienten vorgesehen. Anhand dieser Daten werden dann die beiden Verfahren untereinander verglichen.

## 6.4 Durchführung und Beendigung

Die Teilnahme an der Studie ist vollständig freiwillig. Ein Patient kann jederzeit eine bereits gegebene Einwilligung in die Studienteilnahme, ohne Angabe von Gründen, widerrufen. Präoperativ wird mit dem Patienten ein Reevaluationstermin in 1, 2 und 6 Wochen postooperativ besprochen. Zudem sollte der Patient wissen, dass er nach 1, 2, 6 Wochen und in einem halben Jahr einen Fragebogen ausfüllen muss und eine klinische Kontrolle erfolgt. Nur wenn er dem zustimmt, kann er in die Studie eingeschlossen werden. Für Patienten die in die Studie engewilligt haben, finden sich verschlossene Briefumschläge im Operationssaal. Jeder Briefumschlag enthält ein Blatt mit der Aufschrift VacoHand oder Gipsschiene. Je nach Umschlag soll der Patient primär die VacoHand®oder die Unterarmgipsschiene insgesamt für 1 Woche tragen (soweit vom Operateur nichts anderes vorgegeben). Dann erfolgt 1 Woche postoperativ der Wechsel der Schienung der Unterarmgipsschiene auf eine VacoHand® Versorgung und umgekehrt bei Patienten die primär eine VacoHand®Versorgung hatten, erfolgt der Wecchsel auf die Unterarmgipsschiene.


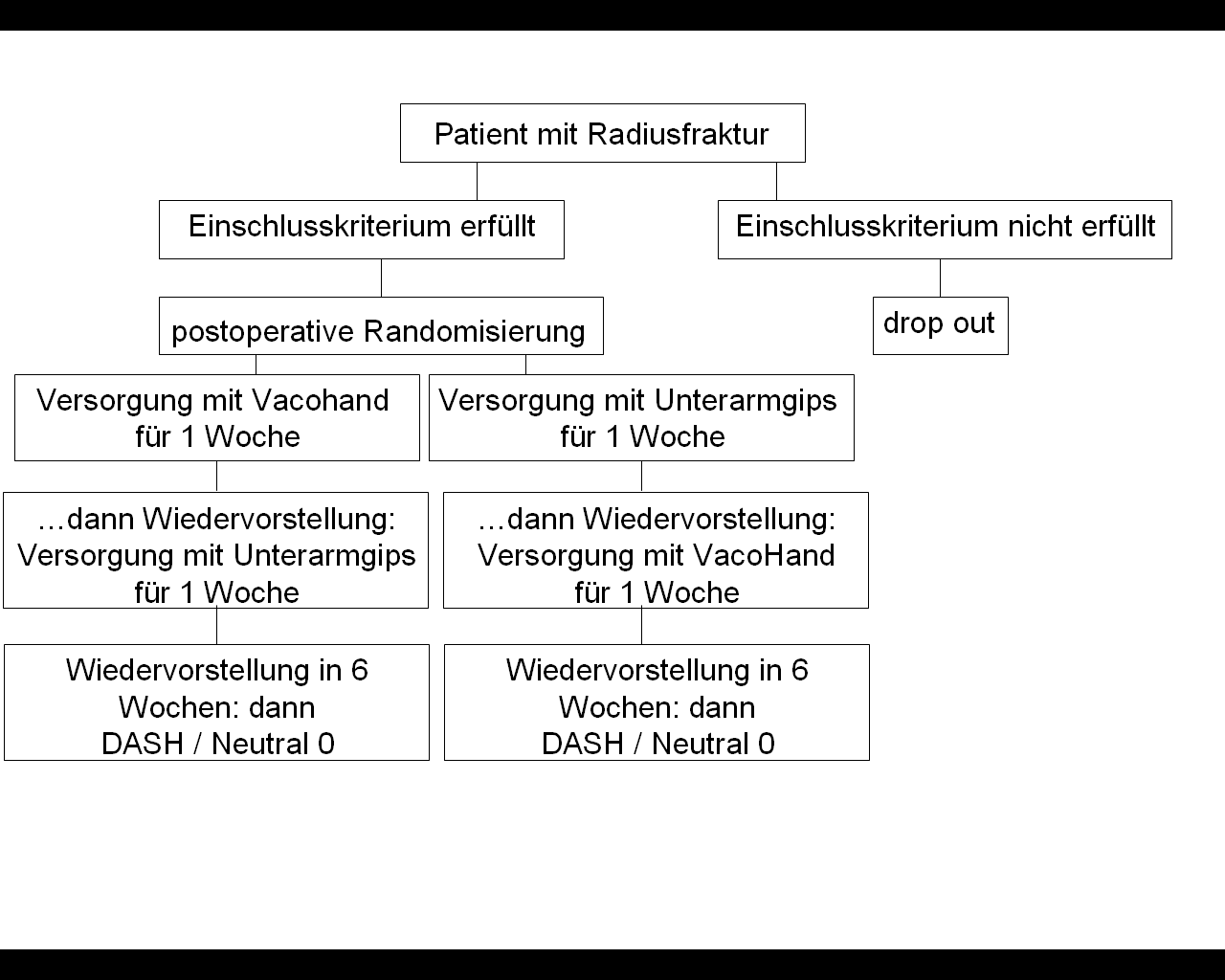


## 6.5 Datenschutz

Die Patientendaten werden in einer Datenbank in der Unfall- und Wiederherstellungschirurgie, Berufsgenossenschaftliche Unfallklinik Tübingen erfasst und gespeichert, eine Löschung der studienbedingten Daten ist nach 10 Jahren vorgesehen. Zugangsberechtigt für die Daten, sind der Leiter und die Autoren dieser Studie, sowie von dem Leiter und den Autoren der Studie ausgewählte wissenschaftliche Mitarbeiter. Die Daten werden, wenn notwendig, nur in pseudonymisierter Form weitergegeben. Bei Studienabbruch oder bei Widerruf der Genehmigung des Patienten, werden die Daten komplett gelöscht. Für die aus dem Projekt resultierenden Publikationen werden nur pseudonymisierte Daten verwendet.

## 6.6 Ethische Belange

Eine spezielle Personenversicherung sowie Wegeunfallversicherung wird nicht abgeschlossen. Der Prüfplan wird durch die zuständige Ethik-Kommission begutachtet.

## 6.7 Aufklärung der Studienteilnehmer

Das Informationsblatt für die Studienteilnehmer, der Fragebogen und die Einwilligungserklärung liegen den Anlagen bei.

# Methoden zur Erfassung der Wirksamkeit und Sicherheit

## 7.1 Risiken und Nebenwirkungen

Die Risiken entsprechen den üblichen Risiken einer Radiusfrakturoperation.

# Angaben zur Statistik, Auswertung

Die Auswertung erfolgt in der Medizinische Biometrie in Tübingen.

# Datenmanagement

Die Patientendaten werden in einer Datenbank in der Unfall- und Wiederherstellungschirurgie, Berufsgenossenschaftliche Unfallklinik Tübingen erfasst und gespeichert, eine Löschung der studienbedingten Daten ist nach 10 Jahren vorgesehen. Zugangsberechtigt für die Daten, sind der Leiter und die Autoren dieser Studie, sowie von dem Leiter und den Autoren der Studie ausgewählte wissenschaftliche Mitarbeiter. Die Daten werden, wenn notwendig, nur in pseudonymisierter Form weitergegeben Bei Studienabbruch oder bei Widerruf der Genehmigung des Patienten, werden die Daten komplett gelöscht. Für die aus dem Projekt resultierenden Publikationen werden nur pseudonymisierte Daten verwendet.

# Ethische Belange, Versicherung

## 10.1 Deklaration von Helsinki

Die Deklaration von Helsinki wird auf die klinische Prüfung angewandt.

## 10.2 Versicherung

Eine spezielle Personenversicherung sowie Wegeunfallversicherung wird nicht abgeschlossen. Der Prüfplan wird durch die zuständige Ethik-Kommission begutachtet.

# Allgemeine Bestimmungen, Vereinbarungen, organisatorische Abläufe

## 11.1 Angaben zur Finanzierung

Finanzierung über abteilungseigene Hausmittel.

## 11.2 Publikationen

Publikationen werden vom Leiter der Studie (Autoren der Studie) erstellt.

## Anhang:

## Anlage 1: Perimed-Bogen

## Anlage 2: SF-36 Fragebogen

## Anlage 3: Einverständniserklärung
