# Supplementary material for: Early Functional Postoperative Therapy of Distal Radius Fracture with a Dynamic Orthosis: Results of a Prospective Randomized Cross-Over Comparative Study
Source: PLoS One. 2015 Mar 30;10(3):e0117720. doi: 10.1371/journal.pone.0117720 (PMC4378993; doi:10.1371/journal.pone.0117720)
Supplement: S2 Protocol — (DOCX) [file pone.0117720.s003.docx]

**Version 1.0**

**Protocol**

**of the study**

**Outcome analysis of the therapy of a radius fracture with**

**VacoHand**^®^ **/ forearm splint**

**Prospective clinical cross-over study**

| **Short title of the clinical study:** | **VACUM** |
| --- | --- |
|  |  |
| **Version:** | **1** |
|  |  |
| **Date of the protocol:** | **01.03.2011** |
|  |  |
| **Principal investigator:** | **Dr.med. Fabian Stuby** |

Table of contents

1 General information 4

1.1 People in charge and signatures: 4

1.2 Addresses 5

1.3 Summary of the study protocol (synopsis) 6

2 Introduction 7

2.1 Summary 7

3 Goals of the study 8

4 Study design 8

4.1 Design 8

4.2 Duration of the study 8

5 Study population 8

5.1 Recruiting 8

5.2 Inclusion criteria 8

5.3 Exclusion criteria 10

5.4 Enrollment into the study 10

6 Course of the study and methods of investigation 10

6.1 Clinical examinations 10

6.2 Study-related sample taking 10

6.3 Planned testing 10

6.4 Implementation and finalization 10

6.5 Data protection 11

6.6 Ethical considerations 12

6.7 Information of the subjects 12

7 Methods for the assessment of effectivity and safety 12

7.1 Risks and side effects 12

8 Information on statistics, data analysis 12

9 Data management 12

10 Ethical considerations, insurance 12

10.1 Declaration of Helsinki 12

10.2 Insurance 13

11 General information, agreements, organisational procedures 13

11.1 Information on funding 13

11.2 Publications 13

Annex: 13

Appendix 1: Perimed patient information form 13

Appendix 2: SF-36 questionnaire 13

Appendix 3: Consent form 13

# General information

## 1.1 People in charge and signatures:

Dr.med. Fabian Stuby

Date Name Signature

Dr. med. Atesch Ateschrang

Date Name Signature

Dr. med. D. Zieker

Date Name Signature

## 1.2 Addresses

**Principal investigators and authors of the prospective clinical cross-over study**

| 1. Dr. med. Fabian Stuby  Unfall- und Wiederherstellungschirurgie, Berufsgenossenschaftliche Unfallklinik Tübingen  Schnarrenbergstr. 95, 72076 Tübingen  Tel.: ++49 7071 – 60 60  Email: fstuby@bgu-tuebingen.de  2. Dr. med. Atesch Ateschrang  Unfall- und Wiederherstellungschirurgie, Berufsgenossenschaftliche Unfallklinik Tübingen  Schnarrenbergstr. 95, 72076 Tübingen  Tel.: ++49 7071 – 60 60  Email: aateschrang@bgu-tuebingen.de  3. Dr. med. Derek Zieker  Universitätsklinik für Allgemeine, Viszeral- und Transplantationschirurgie  Hoppe-Seyler-Str. 3, 72076 Tübingen  Tel.: ++49 7071 – 29 85073  Email: [derek.zieker@med.uni-tuebingen.de](mailto:derek.zieker@med.uni-tuebingen.de) |
| --- |

Competent ethics committee for the LKP

| Chairman: Prof. Dr. Dieter Luft  Executive Research Associate: Dr. Olga Scheck  Secretariat: Karin Zähres, Anett Rönnfeld  Universitätsklinikum Tübingen, Schleichstr. 8, 72076 Tübingen  Tel.: 07071/297 7661  Fax: 07071/29 5965  E-mail: [ethik.kommission@med.uni-tuebingen.de](mailto:ethik.kommission@med.uni-tuebingen.de) |
| --- |

## 1.3 Summary of the study protocol (Synopsis)

| Study code | VACUM | |
| --- | --- | --- |
| Title of the study | Outcome analysis of the therapy of a radius fracture with  VacoHand®/ forearm splint | |
| Study design | Prospective clinical cross-over study | |
| Planned number of patients: | 50 patients | |
| Duration of the study | Planned beginning of the study:  Planned total duration of the study: | 5/2011  18 months |
| Primary endpoint | The primary endpoint of this study is the patient satisfaction, which is evaluated according to the SF-36 questionnaire. Secondary endpoints are the determination of the DASH Score and of the Neutral-0 Method for the hand as well as for the length of the period of disability. | |
| Inclusion criteria | 18 years of age and AO2.3 A2, A3, B1, B2, B3, C1, C2 fractures which need to be operated with a volar stable plate (2.4mm, 3.5mm, all types) | |
| Exclusion criteria | - Less than 18 years of age - All other AO 2.3 fractures - Open fractures and concomitant injuries - Forearm fractures (except Proc. Styloideus ulnae) - Pathological fractures - Patients, who are unable to support the postoperative treatment by themselves | |

# 2 Introduction

## 2.1 Summary

The distal radius fracture is one of the most common disease patterns in trauma surgery with an incidence of two to three per 1,000 inhabitants. It is a distal fracture of the radius. As it is located close to the wrist, it is commonly referred to as a broken wrist. It occurs in all age groups, but mostly between 6 and 18 years and between 60 and 70 years. The formation mechanism is triggered by a traumatic fall on the extended (Colles fracture), or, more rarely, on the bent wrist (Smith fracture). In children, the distal radius fracture occurs mainly during sports. In adults, however, it is mainly caused by tripping and falling the on uneven or slippery ground. The goal of every therapy, either conservative or operative, is the anatomical reconstruction of the joint surface. Unstable extraarticular and intraarticular fractures are mostly stabilized by plate osteosyntheses, which are mostly fixed to the flexor side, rarely also to the extensor side of the radius close to the wrist. The main advantage of plating is the immediate practice safety, so that the physiotherapy can be started shortly after the postoperative application of the cast,. An early mobilisation can prevent the negative consequences of joint immobilization, like e.g. muscular atrophies, joint immobilisation, tendon conglutination, and capsule contraction. Several studies have demonstrated that the immobilisation provokes a considerable degree of muscular atrophy, especially during the first two weeks. Moreover, it causes metabolic, neurological, and functional adaptations, so that the muscular strength may decrease by half after 4-6 weeks of immobilisation in the cast. A possible solution for this problem is the use of a dynamic vacuum orthesis: It provides the stability of a circular forearm splint, and which therefore allows for an early low-risk increase of load, as well as for a limited mobility in the wrist. Furthermore, the comfort of the patients is increased by the fact that an othesis is less heavy than a splint and can be easily applied by the patient himself after having received an instruction. This enables a regular body care. Besides, the limited mobility of the wrist allows for establishing a more physiological functional image. Moreover, a favourable progress of the postoperative phase could reduce the period of disability in the workforce.

# 3 Goals of the study

The primary endpoint of this study is patient satisfaction, which is measured by the SF-36 questionnaire, after AO2.3 A2, A3, B1, B2, B3, C1, C2 fractures requiring surgery, with a volar angular stable plate (2.4mm, 3.5mm, all types), which are treated postoperatively with a VacoHand^®^/ splint. Targeted surveys relating to pain and the quality of life are carried out. Based on these data, both postoperative treatment methods are compared to each other. Secondary endpoints are the determination of the DASH Score and of the Neutral-0 method for the hand, as well as the duration of the period of disability. In order to achieve these goals, follow-up examinations will be carried out 1, 2, and 6 weeks as well as 6 months after the postoperative treatment.

# Study design

## 4.1 Design

This study is a prospective clinical cross-over study

.

## 4.2 Duration of the study

The duration of the study, including data collection, should not exceed 18 months.

# Study population

## 5.1 Recruiting

The recruting of patients takes place at the Clinic for Trauma and Restorative Surgery, BG Klinik Tübingen. The patients will be infformed on the study, will receive the information leaflet and have a consultation, in which all unresolved issues will be clarified.

## 5.2 Inclusion criteria

- At least 18 years old
- AO2.3 A2, A3, B1, B2, B3, C1, C2 fractures requiring surgery, which have been treated with a volar angular stable plate (2.4mm, 3.5mm, alle types)
- Soft tissue G0, G1
- Patients have to be able to actively support the postoperative treatment.

**AO classification of the distal radius fracture:**


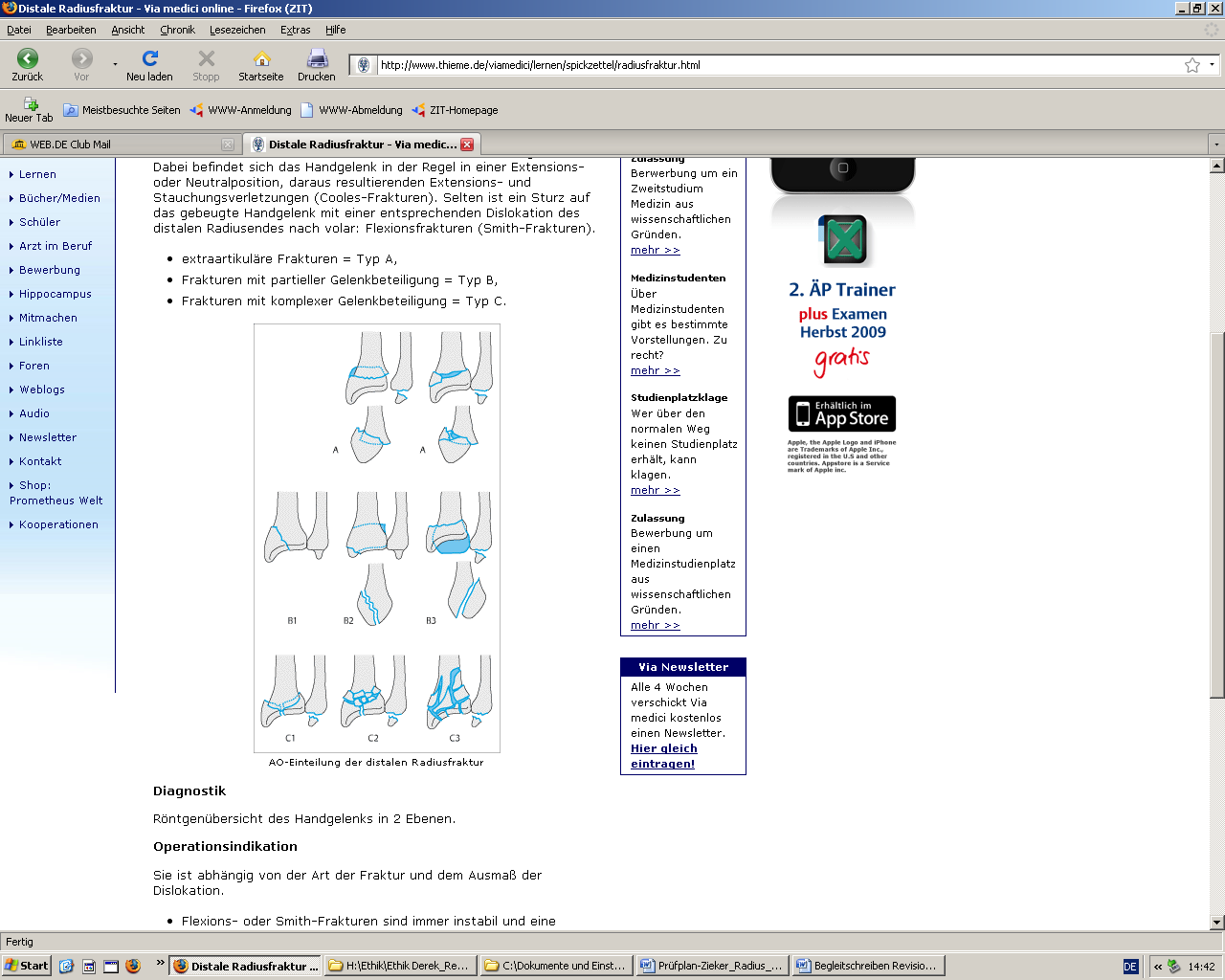


Copied from Thieme „via medici“

#### **Closed fractures (Soft tissue classification):**

- Grade 0: No or insignificant soft tissue injury, indirect trauma, simple fracture
- Grade I: Superficial abrasion or contusion by fragment pressure from the inside, simple to moderate fracture
- Grade II: Deep, dirty abrasion, contusion by direct trauma, imminent compartment syndrome, moderate to severe fracture
- Grade III: Extensive contusion or destruction of the musculature, subcutaneous avulsion of the skin, manifest compartment syndrome, injury of a main vessel

## 5.3 Exclusion criteria

- Less than 18 years old
- All other AO 2.3 Fractures
- Soft tissues G2, G3
- Open fractures and concomitant injuries
- Forearm fractures (except Proc. Styloideus ulnae)
- Pathological fractures
- Patients who are unable to support the postoperative treatment by themselves

## 5.4 Enrollment into the study

All patients who neet the inclusion criteria, and do not fulfill any exclusion criterion, are included ib the study (see recruiting, 5.1).

# Course of the study and methods of investigation

## 6.1 Clinical examinations

Clinical examinations will be carried out in order to confirm the diagnosis, as a control, and to evaluate the satisfaction of the patients as well as the evolution of pain.

## 6.2 Study-related

Not applicable.

## 6.3 Planned testing

Regular clinical follow-up examinations and controls as well as targeted surveys regarding pain and patient satisfaction are planned. Based on these data, both methods can be compared to each other.

## 6.4 Implementation and finalization

The participation at this study is entirely voluntary. A patient can revoke his consent to his participation in the study at any time without stating reasons. Preoperatively, appointments for reevaluation are fixed 1, 2, and 6 weeks postoperatively. Moreover, the patients should know that, after 1, 2, and 6 weeks, as well as after 6 months, he has to fill out a questionnaire and that a clinical control will take place. Only, if a patient agrees to these conditions, he can be included in the study. For those patients, which have agreed to participate in the study, closed envelopes are deposited in the operating theatre. Each envelope contains a piece of paper reading “VacoHand” or “splint”. Depending on the envelope, the patient has to wear initially the VacoHand® or the splint for 1 week in total (unless the operating surgeon indicates otherwise). Then, 1 week postoperative, the splinting is changed from the forearm splinting to the VacoHand® and vice versa.


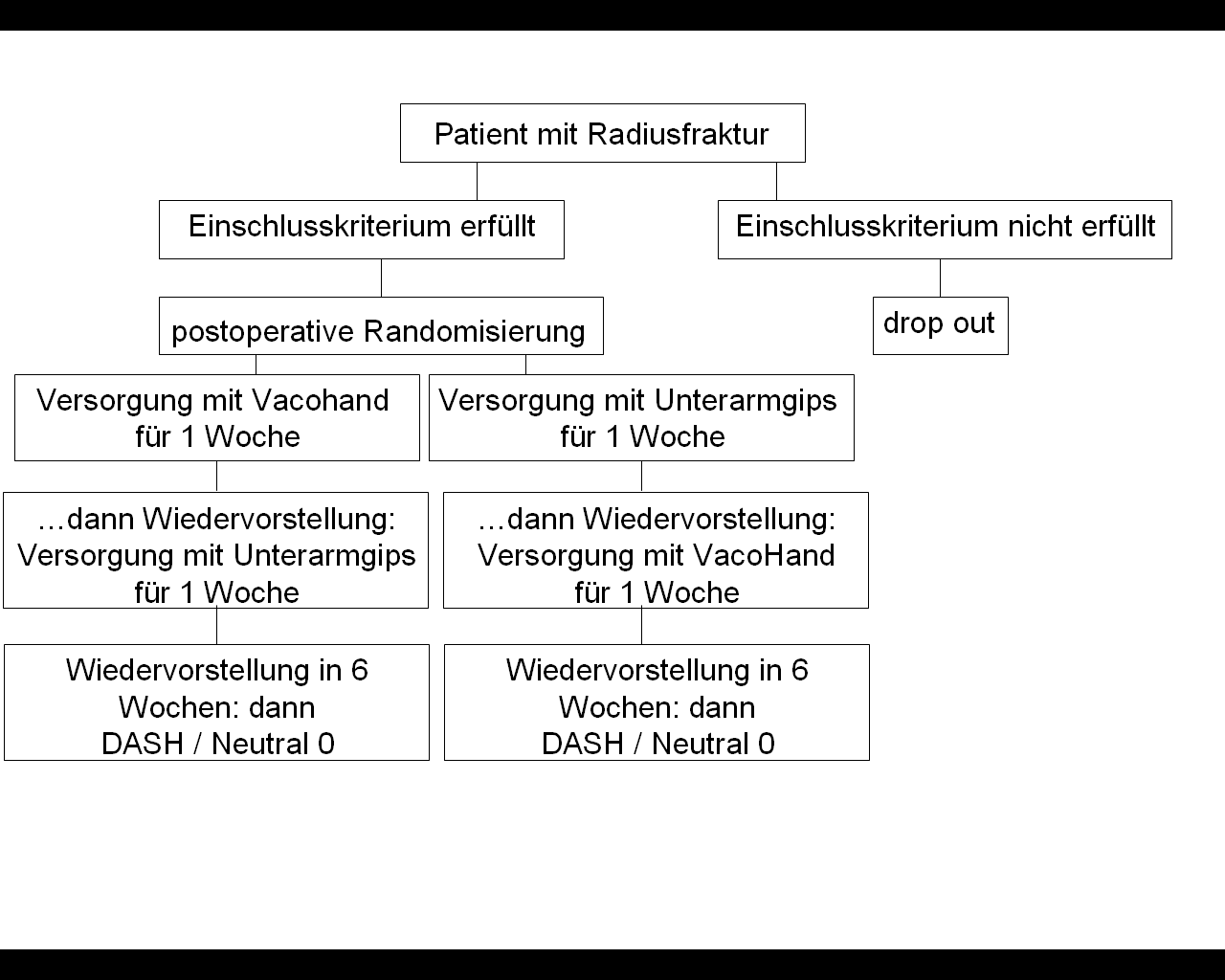


## 6.5 Data protection

The patient data are registered and stored in a database at the Clinic for Trauma and Restorative Surgery, BG Klinik Tübingen. The deletion of the data, which are related to the study, is scheduled to take place in 10 years. Only the principal investigator and the authors of this study as well as research associates which have been selected by the authors of the study are authorized to access these data. The data will only be shared only in a pseudonymized fashion, if necessary. In case of a drop out or of the revocation of the permission by the patient, the data will be completely deleted. For the publications resulting from this project, only pseudomynized data will be used.

## 6.6 Ethical considerations

No specific personal insurance nor travel accident insurance will be effected. The study protocol will be reviewed by the competent ethics committee.

## 6.7 Information of the subjects

The information leaflet for the subjects, the questionnaire, and the consent form are annexed.

# Methods for the assessment of effectivity and safety

## 7.1 Risks and side effects

The risks correspond to the common risks of a radius fracture operation.

# Information on statistics, data analysis

Data analysis will be carried out at the Institute for Clinical Epidemology and Applied Biometry of the University of Tübingen.

# Data management

The patient data are registered and stored in a database at the Clinic for Trauma and Restorative Surgery, BG Klinik Tübingen. The deletion of the data which are related to the study is scheduled to take place in 10 years. Only the principal investigator and the authors of this study as well as research associates which have been selected by the authors of the study are authorized to access these data. The data will only be shared only in a pseudonymized fashion, id necessary. In case of a drop out or of the revocation of the permission by the patient, the data will be completely deleted. For the publications resulting from this project, only pseudomynized data will be used.

# Ethical considerations, insurance

## 10.1 Declaration of Helsinki

The Declaration of Helsinki is applicable to this study.

## 10.2 Insurance

No specific personal insurance nor travel accident insurance will be effected. The study protocol will be reviewed by the competent ethics committee.

# General provisions, agreements, organisational procedures

## 11.1 Information on funding

The study is funded by the section´s own budget.

## 11.2 Publications

Publications are composed by the principal investigator of the study (author of the study).

## Annex:

## Appendix 1: Perimed patient information form

## Appendix 2: SF-36 questionnaire

## Appendix 3: Consent form
