# Supplementary material for: Early Functional Postoperative Therapy of Distal Radius Fracture with a Dynamic Orthosis: Results of a Prospective Randomized Cross-Over Comparative Study
Source: PLoS One. 2015 Mar 30;10(3):e0117720. doi: 10.1371/journal.pone.0117720 (PMC4378993; doi:10.1371/journal.pone.0117720)
Supplement: S1 Raw Data — (PDF) [file pone.0117720.s004.pdf]

Statistische Analyse der Zufriedenheit von VacoHand-Patienten

Dr. Simon Müller

Institut für klinische Pharmakologie, Universitätsklinik Tübingen

7. März 2014

Inhaltsverzeichnis

1 Verwendete Statistik-Software **2**

2 Beschreibung des Datensatzes **2**
2.1 Datenmanipulationen . . . 3
2.2 Beschreibung . . . 3
2.2.1 Geschlecht . . . 3
2.2.2 Altersverteilung . . . 4
2.2.3 AO-Klassifikation . . . 5
2.2.4 Links- bzw. Rechtshänder je Gruppe . . . 6
2.2.5 Traumata Seite und Dominante Hand . . . 6
2.2.6 Entscheidung: VacoHand oder Gipsschine . . . 7
2.2.7 Subgruppenanalyse: Entscheidung: VacoHand oder Gipsschine . . . 8
2.2.8 Subgruppenanalyse: Entscheidung versus Dominante-/Traumahand . . . 8
2.2.9 Subgruppenanalyse: Entscheidung versus Alter . . . 9
2.2.10 Subgruppenanalyse: Entscheidung versus Frakturklassifikation . . . 9

3 Analyse: 7. Tag pOP und 14. Tag pOP **9**
3.1 Fragebogen zur Zufriedenheit . . . 9
3.1.1 Deskriptive Analyse der Items . . . 9
3.1.2 Analyse der Items . . . 9
3.1.3 Frage 1: Schmerzen im Handgelenk . . . 10
3.1.4 Frage 2: Leistungseinschränkung . . . 12
3.1.5 Frage 3: Körperliche Belastbarkeit . . . 13
3.1.6 Frage 4: Die VacoHand / der Gips ist einfach zu handhaben . . . 13
3.1.7 Frage 5: Hygienegefühl in der VacoHand / dem Gips . . . 14
3.1.8 Frage 6: Passgenauigkeit der VacoHand / des Gips . . . 15
3.1.9 Frage 7: Ästhetik der VacoHand / des Gips . . . 16

4 Subgruppenanalyse: Fragebogen zur Zufriedenheit **17**
4.1 Subgruppenanalyse: dominante Hand verletzt bzw. dominante Hand gesund . . . 17
4.1.1 Subgruppenanalyse: dominante Hand verletzt bzw. gesund und Handbarkeit . . . 17
4.1.2 Subgruppenanalyse: dominante Hand verletzt bzw. gesund und Hygienegefühl . . . 20
4.2 Subgruppenanalyse: Alter . . . 22
4.2.1 Subgruppenanalyse: Alter und Handbarkeit . . . 22
4.2.2 Subgruppenanalyse: Alter und Hygienegefühl . . . 25
4.3 Subgruppenanalyse: Frakturklassifikation . . . 28
4.3.1 Subgruppenanalyse: Frakturklassifikation und Handbarkeit . . . 28
4.3.2 Subgruppenanalyse: Frakturklassifikation und Hygienegefühl . . . 32
4.4 SF-36 Fragebogen . . . 36
4.4.1 Analyse der 8 Dimensionen . . . 36

5 Analyse: 1 Monat pOP und 3 Monate pOP **46**
5.1 DASH-Fragebogen 46
5.2 Auswertung des DASH-Scores 46
5.3 Beweglichkeit / Bewegungsdefizite 47
5.4 Bewegungsdefizite: D.Ext 47
5.5 Bewegungsdefizite: P.Flex 49
5.6 Bewegungsdefizite: Sup 51
5.7 Bewegungsdefizite: Pron 53
5.8 Bewegungsdefizite: R.Dukt 55
5.9 Bewegungsdefizite: U.Dukt 57

1 Verwendete Statistik-Software

Für die Analyse wurde die Statistik-Software R (www.r-project.org) in Version 3.0 verwendet. Zusätzlich wurde das R-Paket *coin* in Version 1.0-21 benutzt.

2 Beschreibung des Datensatzes

Die folgenden Ausgaben beschreiben den Datensatz.

```
load("PatDat.RData")
library(Hmisc)
str(data)

'data.frame': 29 obs. of  13 variables:
 $ Studiennummer    : num  1 2 3 4 5 6 9 10 12 14 ...
 $ Gruppe           : chr  "A" "A" "B" "A" ...
 $ Entscheidung     : chr  "G" "G" "V" "V" ...
 $ Geschlecht       : chr  "W" "W" "W" "M" ...
 $ Versicherung     : chr  "BG" "Kasse" "K" "Selbstzahler" ...
 $ Alter.in.J.      : num  52 53 32 46 68 21 57 28 57 63 ...
 $ AO.Klassif.      : chr  "B" "A" "A" "C" ...
 $ Trauma.Seite     : chr  "R" "L" "L" "R" ...
 $ Dom.Hand         : chr  "R" "R" "R" "R" ...
 $ präop.Dauer.in.d : int  8 5 7 10 7 6 6 6 8 8 ...
 $ Postop.Dauer.in.d: int  4 4 3 2 3 3 4 3 4 5 ...
 $ Alter            : chr  ">= 50" ">= 50" "< 50" "< 50" ...
 $ DomTraum         : int  1 0 0 1 1 1 0 1 0 1 ...

describe(data[, c(2:4, 6:9)])

data[, c(2:4, 6:9)]

 7  Variables      29  Observations
--------------------------------------------------------------------
Gruppe
      n missing  unique
     29       0       2

A (15, 52%), B (14, 48%)
--------------------------------------------------------------------
Entscheidung
      n missing  unique
     29       0       2

G (6, 21%), V (23, 79%)
--------------------------------------------------------------------
Geschlecht
```

```
      n missing  unique
     29       0       2

M (8, 28%), W (21, 72%)
-------------------------------------------------------------------
Alter.in.J.
      n missing  unique    Mean     .05     .10     .25     .50
     29       0      24   48.28    21.8    26.2    35.0    53.0
    .75     .90     .95
   62.0    64.0    66.4

lowest : 20 21 23 27 28, highest: 62 63 64 68 75
-------------------------------------------------------------------
AO.Klassif.
      n missing  unique
     29       0       3

A (14, 48%), B (3, 10%), C (12, 41%)
-------------------------------------------------------------------
Trauma.Seite
      n missing  unique
     29       0       2

L (13, 45%), R (16, 55%)
-------------------------------------------------------------------
Dom.Hand
      n missing  unique
     28       1       2

L (5, 18%), R (23, 82%)
-------------------------------------------------------------------
```

2.1 Datenmanipulationen

Der Fall mit der Studiennummer 45 wurde aus der Analyse entfernt, da dieser in der 2. WV aus der Studie ausgeschieden ist. Die AO-Klassifikationen wurden auf die Klassen A bis C beschränkt. Unterklassen wurden nicht betrachtet. Die dominante Hand bei Studiennummer 42 ist unklar. Diese wurde auf NA (dt.: nicht gekennzeichnet) gesezt.

2.2 Beschreibung

2.2.1 Geschlecht

In der Gruppe B sind 14 Fälle und in Gruppe A 15 Fälle. Die Geschlechter verteilen sich wie folgt auf die Gruppen:

	Gruppe A	Gruppe B
M	5	3
W	10	11

Tabelle 1: Absoluter Anteil

	Gruppe A	Gruppe B
M	33%	21%
W	67%	79%

Tabelle 2: Relativer Anteil, gruppenweise

```
Fisher's Exact Test for Count Data

data:  tab
p-value = 0.6817
```

```
alternative hypothesis: true odds ratio is not equal to 1
95 percent confidence interval:
  0.2654 14.6716
sample estimates:
odds ratio
     1.795
```

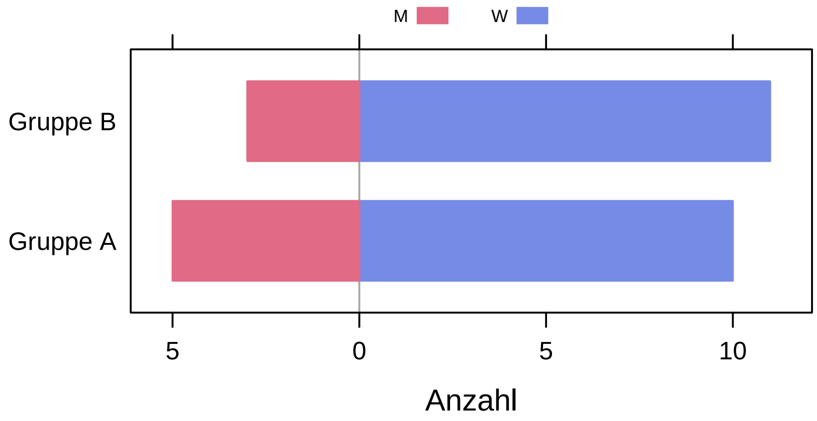

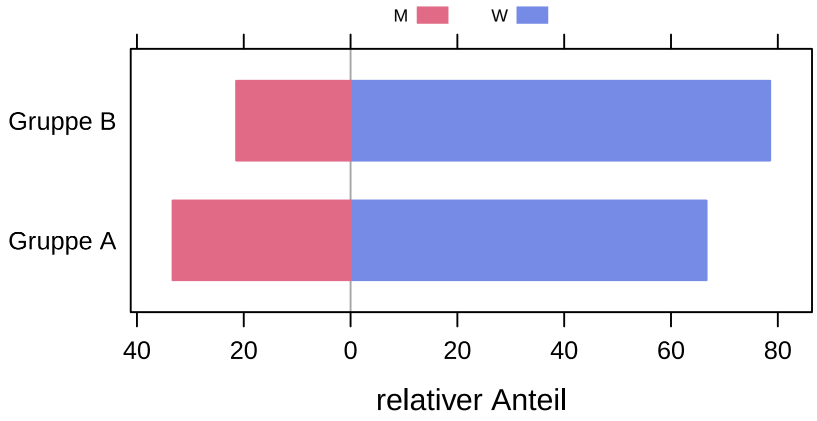

Bei der Verteilung des Geschlechts auf die Gruppen unterscheiden sie sich nicht signifikant (Fisher-Test).

2.2.2 Altersverteilung

Das mittlere Alter der Patienten beträgt $48{,}28$ Jahre. Der jüngste Fall ist 20, der Älteste 75.

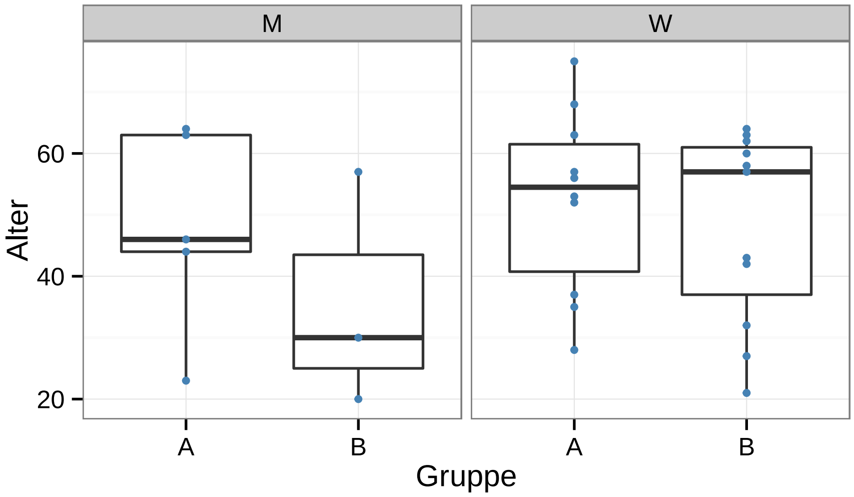

2.2.3 AO-Klassifikation

Für die AO-Klassifikation erhalten wir:

	Gruppe A	Gruppe B
Klasse A	7	7
Klasse B	2	1
Klasse C	6	6

Tabelle 3: Absoluter Anteil

	Gruppe A	Gruppe B
Klasse A	47%	50%
Klasse B	13%	7%
Klasse C	40%	43%

Tabelle 4: Relativer Anteil, gruppenweise

```
Fisher's Exact Test for Count Data

data:  tab
p-value = 1
alternative hypothesis: two.sided
```

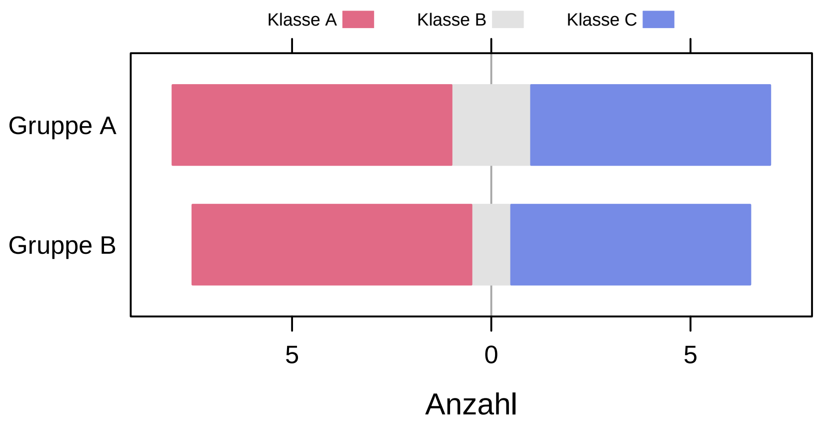

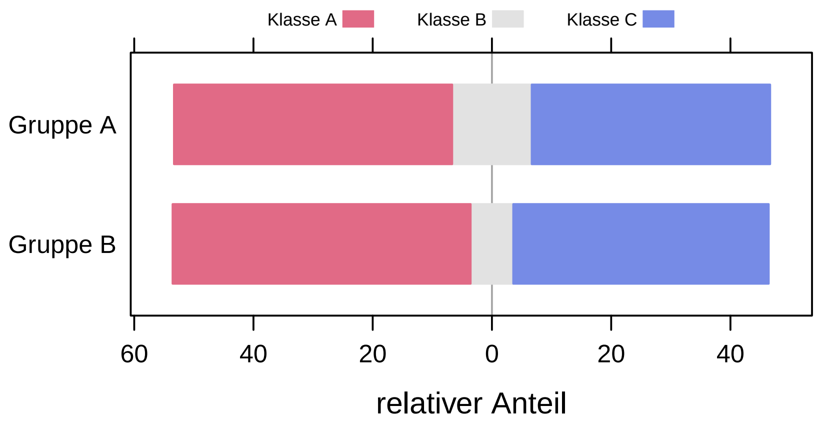

2.2.4 Links- bzw. Rechtshänder je Gruppe

Folgendermassen sind Links- und Rechtshänder auf die Gruppen verteilt:

	Gruppe A	Gruppe B
Linkshänder	4	1
Rechtshänder	10	13

Tabelle 5: Absoluter Anteil

	Gruppe A	Gruppe B
Linkshänder	29%	7%
Rechtshänder	71%	93%

Tabelle 6: Relativer Anteil, gruppenweise

2.2.5 Traumata Seite und Dominante Hand

Die nachfolgenden Tabellen und Grafiken zeigen, in wie weit die dominante Hand der Fälle von dem Trauma betroffen ist.

	Dominante Hand: Links	Dominante Hand: Rechts
Traumata Seite: Links	1	11
Traumata Seite: Rechts	4	12

Tabelle 7: Absoluter Anteil

	Dominante Hand: Links	Dominante Hand: Rechts
Traumata Seite: Links	20%	48%
Traumata Seite: Rechts	80%	52%

Tabelle 8: Relativer Anteil, spaltenweise

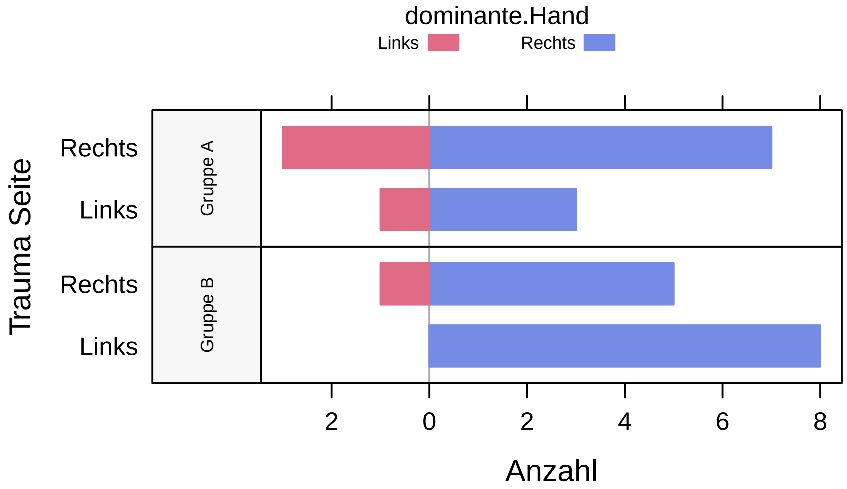

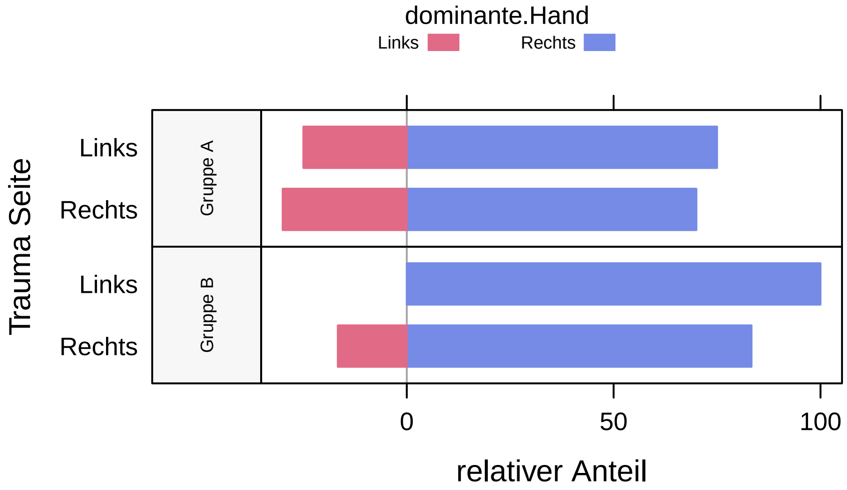

2.2.6 Entscheidung: VacoHand oder Gipsschine

Zum Abschluss der Studie wurden die Patienten gefragt, ob diese den Gips oder die VacoHand in Zukunft wählen würden. Der Fisher-Test zeigt eine signifikanten Unterschied zwischen den beiden Gruppen. Die 23 von 29 entschieden sich für die VacoHand. Die 6 Patienten, die sich für den Gips entschieden habe, waren alle in Gruppe A.

	Gruppe A	Gruppe B
Gipsschiene	6	0
VacoHand	9	14

Tabelle 9: Absoluter Anteil

	Gruppe A	Gruppe B
Gipsschiene	40%	0%
VacoHand	60%	100%

Tabelle 10: Relativer Anteil, gruppenweise

```
Fisher's Exact Test for Count Data
```

```
data:  tab
p-value = 0.01686
alternative hypothesis: true odds ratio is not equal to 1
95 percent confidence interval:
 1.377   Inf
sample estimates:
odds ratio
       Inf
```

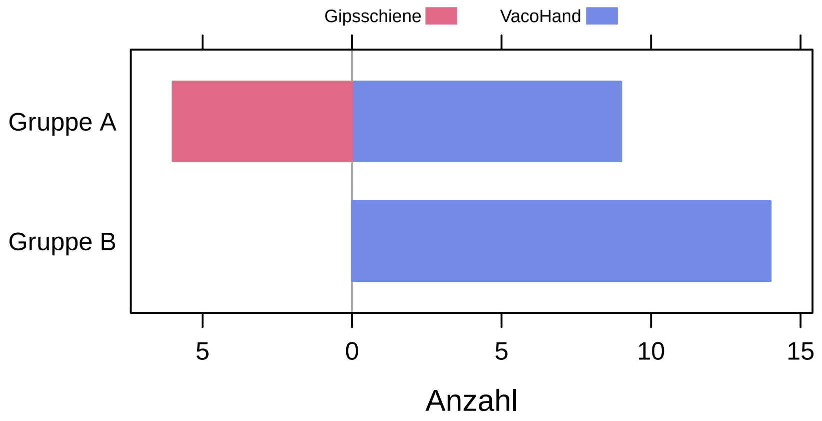

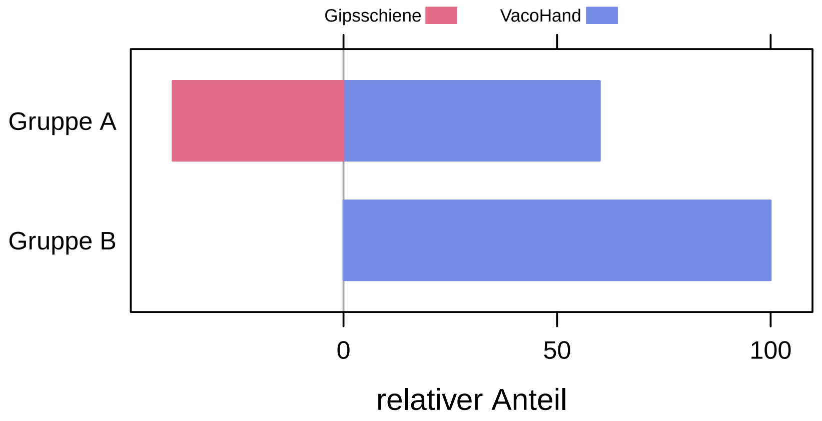

2.2.7 Subgruppenanalyse: Entscheidung: VacoHand oder Gipsschine

In den folgenden Abschnitten wird die Entscheidung zu den Subgruppen Alter (jünger als 50 und älter als 50), die Frakturklassifikation und bzgl. dominante Hand verletzt bzw. gesund analysiert. Für die statistische Analyse wurde der Fisher-Test verwendet. Die Resultate sind nicht aufgeführt. Sie sind alle nicht signifikant.

2.2.8 Subgruppenanalyse: Entscheidung versus Dominante-/Traumahand

	Trauma nicht an Dom. Hand	Trauma an Dom. Hand
Gipsschiene	3	3
VacoHand	3	5

	Trauma nicht an Dom. Hand	Trauma an Dom. Hand
Gipsschiene	0	0
VacoHand	9	5

2.2.9 Subgruppenanalyse: Entscheidung versus Alter

	< 50	>= 50
Gipsschiene	2	4
VacoHand	4	5

```
Error: konnte Funktion "Cairo_pdf"
nicht finden
```

null device 1

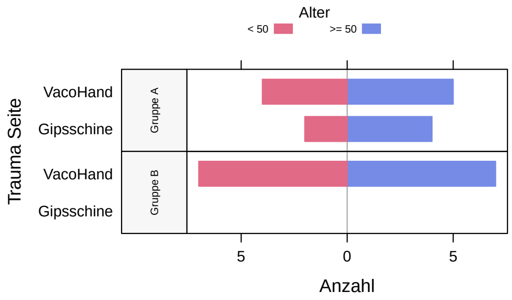

	< 50	>= 50
Gipsschiene	0	0
VacoHand	7	7

2.2.10 Subgruppenanalyse: Entscheidung versus Frakturklassifikation

	A	B	C
Gipsschiene	3	2	1
VacoHand	4	0	5

	A	B	C
Gipsschiene	0	0	0
VacoHand	7	1	6

3 Analyse: 7. Tag pOP und 14. Tag pOP

3.1 Fragebogen zur Zufriedenheit

Die Fragen $1-3$ des Fragebogens sind allgemeine Fragen zur körperlichen Verfassung (Schmerzen, Leistungseinschränkung und körperliche Belastbarkeit) des Patienten. Die Fragen $4-7$ beziehen sich auf die in der Vorwoche verwendete Methode für den Schutz der Hand.
Bei den Fragen 1 und 2 geht die Likert-Skala von 1 = negativ zu 5 = positiv. Bei den Fragen 3 bis 7 vice versa.

3.1.1 Deskriptive Analyse der Items

Im folgenden sind die Antworten tabellarisch aufgeteilt nach Zeitpunkt und Gruppe aufgeführt.

	Absolut richtig	Überw. richtig	Weiß nicht	Überw. falsch	Absolut falsch
Schmerzen im Handgelenk	2	4	0	4	5
Leistungseinschränkung	8	4	0	1	2
Körperliche Belastbarkeit	3	6	1	3	2
einfach zu handhaben	14	0	1	0	0
Hygienegefühl	11	4	0	0	0
Passgenauigkeit	6	8	1	0	0
Ästhetik	9	4	2	0	0

Tabelle 11: Gruppe A, 7. Tag pOP

Die divergierenden gestappelten Balkendiagramme liefern einen ersten Eindruck über die Struktur in den Daten. Es heben sich schon hier die zeitlichen sowie die Gruppenunterschiede heraus, insbesondere, wenn man Gruppe B zum Zeitpunkt 7.Tag pOP betrachtet.

3.1.2 Analyse der Items

Für die Analyse dieser Fragen wurde für den Gruppenvergleich der Cochran-Armitage-Trend-Test verwendet. Die Nullhypothese sagt aus, dass der Trend von in dem Kategorien des Items sich nicht zwischen den Grup-

	Absolut richtig	Überw. richtig	Weiß nicht	Überw. falsch	Absolut falsch
Schmerzen im Handgelenk	2	3	0	4	6
Leistungseinschränkung	7	4	1	0	3
Körperliche Belastbarkeit	5	3	1	3	3
einfach zu handhaben	5	6	0	3	1
Hygienegefühl	2	12	0	1	0
Passgenauigkeit	5	5	0	5	0
Ästhetik	6	6	2	1	0

Tabelle 12: Gruppe A, 14. Tag pOP

	Absolut richtig	Überw. richtig	Weiß nicht	Überw. falsch	Absolut falsch
Schmerzen im Handgelenk	1	10	0	2	2
Leistungseinschränkung	11	4	0	0	0
Körperliche Belastbarkeit	7	3	1	3	1
einfach zu handhaben	3	1	1	5	5
Hygienegefühl	2	0	0	9	4
Passgenauigkeit	7	5	2	1	0
Ästhetik	1	2	4	6	2

Tabelle 13: Gruppe B, 7. Tag pOP

	Absolut richtig	Überw. richtig	Weiß nicht	Überw. falsch	Absolut falsch
Schmerzen im Handgelenk	3	3	0	2	7
Leistungseinschränkung	3	6	0	2	4
Körperliche Belastbarkeit	9	2	1	1	2
einfach zu handhaben	15	0	0	0	0
Hygienegefühl	11	4	0	0	0
Passgenauigkeit	8	7	0	0	0
Ästhetik	7	3	3	2	0

Tabelle 14: Gruppe B, 14. Tag pOP

pen unterscheiden versus es gibt einen Trend zwischen den beiden Gruppen. Für die Innergruppen-Vergleich zwischen den Zeitpunkten wurde der Wilcoxon-Vorzeichen-Rang-Test eingesetzt. Bei sämtlichen Fragen wurde zunächst ein zweiseitiger Test angewendet. Bei signifikanten Resultaten wurde ein einseitiger Test angewendet, um zu bestimmen, welche der beiden Möglichkeiten von den Patienten als die bessere angesehen wird.
Aufgrund des Versuchaufbaus erhält man bei dem Innergruppen Zeitvergleich bei den Fragen $1-3$ eine Aussage über zeitlichen Verlauf der körperlichen Verfassung. Bei den Fragen $4-7$ hingegen einen Vergleich zwischen Gips und VacoHand innerhalb derselben Gruppe.
Die Ausgabe in den nachfolgenden Analysen ist wie folgt aufgebaut: zunächst wird der Gruppenvergleich jeweils für die beiden Zeitpunkte durchgeführt. Anschließend der Vergleich der Zeitpunkte innerhalb der Gruppe A gefolgt von Gruppe B. Je Frage werden also 4 Vergleiche durchgeführt.
Die Abbildung 1 zeigt an, an welchen Punkten man einen signifikanten Unterschied zwischen den Gruppen erwarten würde. Für die grafische Veranschaulichung wird auf diese (Abb. 1) verwiesen.

3.1.3 Frage 1: Schmerzen im Handgelenk

```
Gruppenvergleich 7. Tag pOP:

Asymptotic General Independence Test

data:  VV1 by Gruppe (A, B)
Z = 1.523, p-value = 0.1277
alternative hypothesis: two.sided



Gruppenvergleich 14. Tag pOP:
```

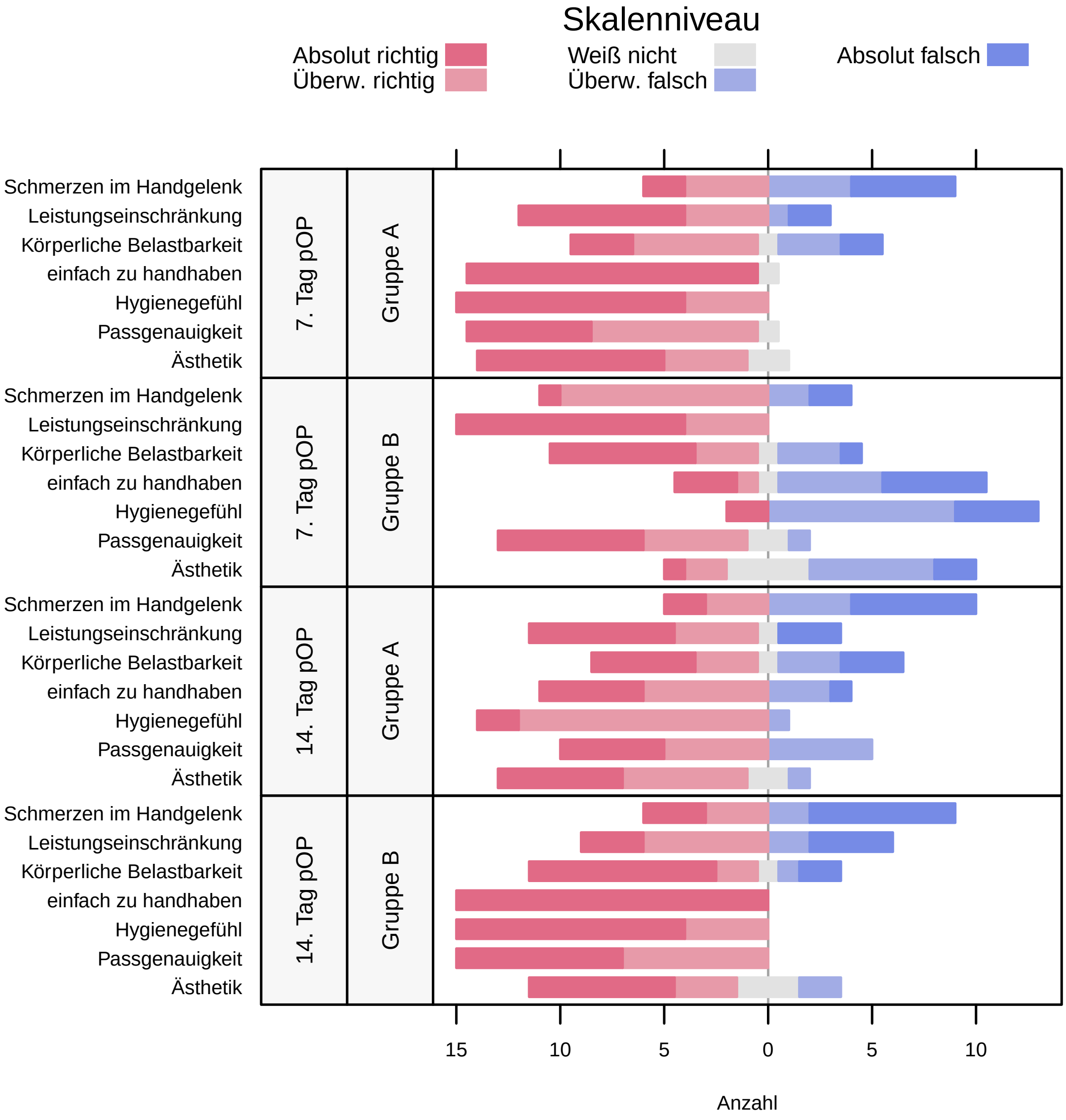

Abbildung 1: Divergierende Balkendiagramme: Fragebogen zur Zufriedenheit

```
Asymptotic General Independence Test

data:  VV1 by Gruppe (A, B)
Z = 0.2264, p-value = 0.8209
alternative hypothesis: two.sided


Zeitvergleich Gruppe A:

Asymptotic Wilcoxon-Signed-Rank Test (zeros handled a la
Wilcoxon)

data:  y by
 x (neg, pos)
 stratified by block
```

```
Z = 0.361, p-value = 0.7181
alternative hypothesis: true mu is not equal to 0


Zeitvergleich Gruppe B:

Asymptotic Wilcoxon-Signed-Rank Test (zeros handled a la
Wilcoxon)

data:  y by
 x (neg, pos)
 stratified by block
Z = 1.462, p-value = 0.1436
alternative hypothesis: true mu is not equal to 0
```

Bezüglich der Schmerzen im Handgelenk konnte weder zwischen den Gruppen noch den Zeitpunkten ein unterschied festegestellt werden.

3.1.4 Frage 2: Leistungseinschränkung

```
Gruppenvergleich 7. Tag pOP:

Asymptotic General Independence Test

data:  VV2 by Gruppe (A, B)
Z = 1.779, p-value = 0.07527
alternative hypothesis: two.sided


Gruppenvergleich 14. Tag pOP:

Asymptotic General Independence Test

data:  VV2 by Gruppe (A, B)
Z = -1.147, p-value = 0.2513
alternative hypothesis: two.sided


Zeitvergleich Gruppe A:

Asymptotic Wilcoxon-Signed-Rank Test (zeros handled a la
Wilcoxon)

data:  y by
 x (neg, pos)
 stratified by block
Z = 0.6378, p-value = 0.5236
alternative hypothesis: true mu is not equal to 0


Zeitvergleich Gruppe B:

Asymptotic Wilcoxon-Signed-Rank Test (zeros handled a la
Wilcoxon)

data:  y by
 x (neg, pos)
```

```
 stratified by block
Z = 2.971, p-value = 0.002968
alternative hypothesis: true mu is not equal to 0
```

Zum 7. Tag pOP empfinden mehr Patienten in Gruppe B, die Gruppe mit dem Gips, eine Einschränkung ihrer Leistung als in Gruppe A, die die VacoHand hatte. Zum ZP 2 kann kein statistischer Unterschied zwischen den Gruppen ermittelt werden. Beide Gruppen haben keine Leistungseinschränkungen. Beim Innergruppenvergleich ist die Änderung der Leistungseinschränkung signifikant und auch grafisch deutlich sichtbar.

3.1.5 Frage 3: Körperliche Belastbarkeit

```
Gruppenvergleich 7. Tag pOP:

Asymptotic General Independence Test

data:  VV3 by Gruppe (A, B)
Z = 0.9087, p-value = 0.3635
alternative hypothesis: two.sided


Gruppenvergleich 14. Tag pOP:

Asymptotic General Independence Test

data:  VV3 by Gruppe (A, B)
Z = 1.266, p-value = 0.2055
alternative hypothesis: two.sided


Zeitvergleich Gruppe A:

Asymptotic Wilcoxon-Signed-Rank Test (zeros handled a la
Wilcoxon)

data:  y by
 x (neg, pos)
 stratified by block
Z = 0.1197, p-value = 0.9047
alternative hypothesis: true mu is not equal to 0


Zeitvergleich Gruppe B:

Asymptotic Wilcoxon-Signed-Rank Test (zeros handled a la
Wilcoxon)

data:  y by
 x (neg, pos)
 stratified by block
Z = -0.3602, p-value = 0.7187
alternative hypothesis: true mu is not equal to 0
```

Die Art der Ruhigstellung der Hand hat keinen messbaren Einfluss auf die körperliche Belastbarkeit.

3.1.6 Frage 4: Die VacoHand / der Gips ist einfach zu handhaben

```
Gruppenvergleich 7. Tag pOP:

Asymptotic General Independence Test

data:  VV4 by Gruppe (A, B)
Z = -3.941, p-value = 4.059e-05
alternative hypothesis: less


Gruppenvergleich 14. Tag pOP:

Asymptotic General Independence Test

data:  VV4 by Gruppe (A, B)
Z = 3.072, p-value = 0.001061
alternative hypothesis: greater


Zeitvergleich Gruppe A:

Asymptotic Wilcoxon-Signed-Rank Test (zeros handled a la
Wilcoxon)

data:  y by
 x (neg, pos)
 stratified by block
Z = 2.358, p-value = 0.009198
alternative hypothesis: true mu is greater than 0


Zeitvergleich Gruppe B:

Asymptotic Wilcoxon-Signed-Rank Test (zeros handled a la
Wilcoxon)

data:  y by
 x (neg, pos)
 stratified by block
Z = -3.108, p-value = 0.0009431
alternative hypothesis: true mu is less than 0
```

Beim Gruppenvergleich kann man zu beiden Zeitpunken nachweisen, dass die Handhabbarkeit der VacoHand dem Gips überlegen ist. Beim Wechsel vom Gips zur VacoHand und umgekehrt gibt es eine deutlich messbare Verschiebung der Handhabbarkeit.

3.1.7 Frage 5: Hygienegefühl in der VacoHand / dem Gips

```
Gruppenvergleich 7. Tag pOP:

Asymptotic General Independence Test

data:  VV5 by Gruppe (A, B)
Z = -4.417, p-value = 5.007e-06
alternative hypothesis: less


Gruppenvergleich 14. Tag pOP:
```

```
Asymptotic General Independence Test

data:  VV5 by Gruppe (A, B)
Z = 3.003, p-value = 0.001335
alternative hypothesis: greater


Zeitvergleich Gruppe A:

Asymptotic Wilcoxon-Signed-Rank Test (zeros handled a la
Wilcoxon)

data:  y by
 x (neg, pos)
 stratified by block
Z = 2.673, p-value = 0.003763
alternative hypothesis: true mu is greater than 0


Zeitvergleich Gruppe B:

Asymptotic Wilcoxon-Signed-Rank Test (zeros handled a la
Wilcoxon)

data:  y by
 x (neg, pos)
 stratified by block
Z = -3.244, p-value = 0.0005897
alternative hypothesis: true mu is less than 0
```

Zu beiden Zeitpunkten zeigt sich, dass das Hygienegefühl in der VacoHand besser ist als im Gips. Im Zeitvergleich sieht man, dass die Gruppe B von einem sehr negativen Hygienegefühl zu einem sehr positivem wechselt. In Gruppe A hingegen fällt wechselt das Hygienegefühlt nicht so radikal wie in Gruppe B. Es wird auch noch im Gips als sehr hygienisch empfunden.

3.1.8 Frage 6: Passgenauigkeit der VacoHand / des Gips

```
Gruppenvergleich 7. Tag pOP:

Asymptotic General Independence Test

data:  VV6 by Gruppe (A, B)
Z = -0.4652, p-value = 0.6418
alternative hypothesis: two.sided


Gruppenvergleich 14. Tag pOP:

Asymptotic General Independence Test

data:  VV6 by Gruppe (A, B)
Z = 2.235, p-value = 0.02541
alternative hypothesis: two.sided


Zeitvergleich Gruppe A:
```

```
Asymptotic Wilcoxon-Signed-Rank Test (zeros handled a la
Wilcoxon)

data:  y by
 x (neg, pos)
 stratified by block
Z = 1.635, p-value = 0.1021
alternative hypothesis: true mu is not equal to 0


Zeitvergleich Gruppe B:

Asymptotic Wilcoxon-Signed-Rank Test (zeros handled a la
Wilcoxon)

data:  y by
 x (neg, pos)
 stratified by block
Z = -1.232, p-value = 0.218
alternative hypothesis: true mu is not equal to 0
```

Zum ZP 2 kann ein signifikanter Unterschied festgestellt werden. In der Grafik ist zu beobachten, dass die gefühlte Passgenauigkeit in Gruppe A abnimmt, in Gruppe B hingegen zunimmt. Statistisch lässt sich dieser zeitliche Effekt nicht beobachten.

3.1.9 Frage 7: Ästhetik der VacoHand / des Gips

```
Gruppenvergleich 7. Tag pOP:

Asymptotic General Independence Test

data:  VV7 by Gruppe (A, B)
Z = -3.837, p-value = 0.0001244
alternative hypothesis: two.sided


Gruppenvergleich 14. Tag pOP:

Asymptotic General Independence Test

data:  VV7 by Gruppe (A, B)
Z = -0.3598, p-value = 0.719
alternative hypothesis: two.sided


Zeitvergleich Gruppe A:

Asymptotic Wilcoxon-Signed-Rank Test (zeros handled a la
Wilcoxon)

data:  y by
 x (neg, pos)
 stratified by block
Z = 1.089, p-value = 0.2763
alternative hypothesis: true mu is not equal to 0
```

```
Zeitvergleich Gruppe B:

Asymptotic Wilcoxon-Signed-Rank Test

data:  y by
 x (neg, pos)
 stratified by block
Z = -2.423, p-value = 0.01538
alternative hypothesis: true mu is not equal to 0
```

Zum Zeitpunkt 1 gibt es einen deutlichen Unterschied beim Empfinden der Ästhetik der Gruppe A und B. In Gruppe B verbessert sich das Ästhetik Gefühl beim Wechsel vom Gips zur VacoHand. Dieser Effekt tritt bei Gruppe A nicht auf. Das Ästhetik- Empfinden verschlechtert sich nicht messbar.

4 Subgruppenanalyse: Fragebogen zur Zufriedenheit

Von besonderem Interesse ist die Handbarkeit und die Hygiene der Schienen (Item 4 des Fragebogens) bzgl. dominante Hand verletzt bzw. gesund, die Frakturklassifikation und das Alter.
Die ersten drei Fragen des Zufriedenheitsfragebogens betreffen zum einen nicht direkt die Schiene und zeigen in der Gesamtanalyse auch nichts auffälliges beim Vergleich zwischen den Schienen.
Die Passgenauigkeit und die Ästhetik (?) werden nicht weiter untersucht, da zum einen bei der Passgenauigkeit anhand der Ergebnisse und der Erwartung nichts weiter zu erwarten ist. Die Ästhetik ist was die Resultate betrifft ein Borderliner und wird zunächst nicht weiter untersucht, da diese Fragestellung nicht von besonderem Interesse ist.

4.1 Subgruppenanalyse: dominante Hand verletzt bzw. dominante Hand gesund

4.1.1 Subgruppenanalyse: dominante Hand verletzt bzw. gesund und Handbarkeit

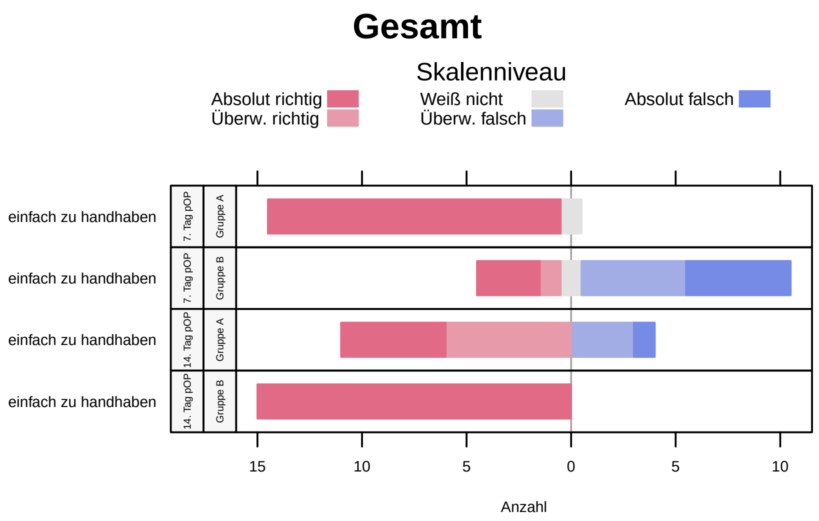

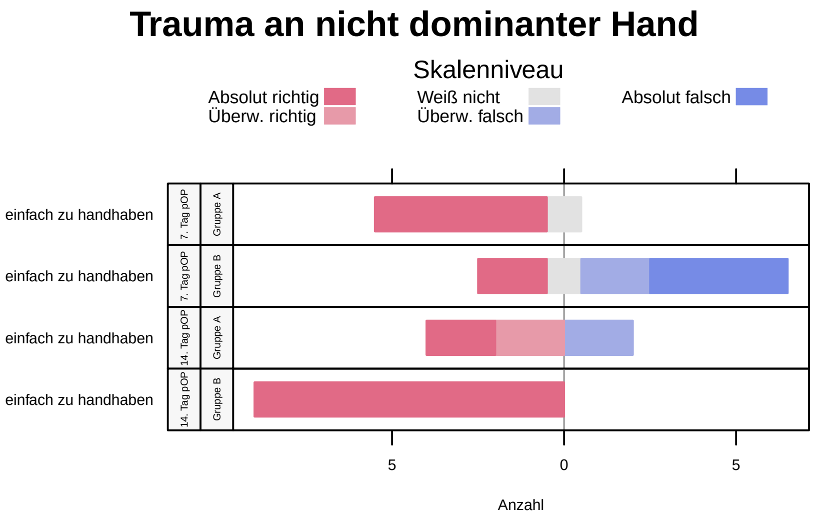

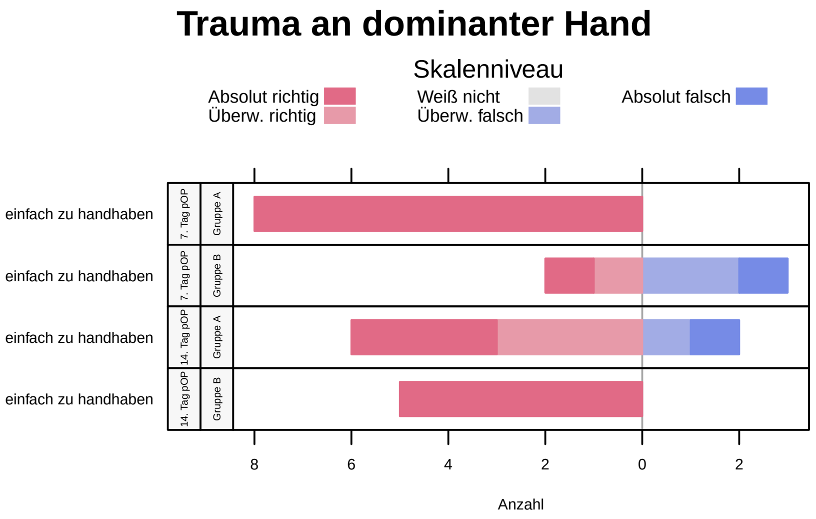


```
Trauma an nicht dominanter Hand
===============================
Gruppenvergleich 7. Tag pOP:

Asymptotic General Independence Test

data:  VV4 by Gruppe (A, B)
Z = -3.941, p-value = 4.059e-05
alternative hypothesis: less


Gruppenvergleich 14. Tag pOP:

Asymptotic General Independence Test

data:  VV4 by Gruppe (A, B)
Z = 3.072, p-value = 0.001061
alternative hypothesis: greater


Zeitvergleich Gruppe A:

Asymptotic Wilcoxon-Signed-Rank Test (zeros handled a la
```

```
Wilcoxon)

data:  y by
 x (neg, pos)
 stratified by block
Z = 2.358, p-value = 0.009198
alternative hypothesis: true mu is greater than 0


Zeitvergleich Gruppe B:

Asymptotic Wilcoxon-Signed-Rank Test (zeros handled a la
Wilcoxon)

data:  y by
 x (neg, pos)
 stratified by block
Z = -3.108, p-value = 0.0009431
alternative hypothesis: true mu is less than 0
```

```
Trauma an dominanter Hand
=========================
Gruppenvergleich 7. Tag pOP:

Asymptotic General Independence Test

data:  VV4 by Gruppe (A, B)
Z = -3.941, p-value = 4.059e-05
alternative hypothesis: less


Gruppenvergleich 14. Tag pOP:

Asymptotic General Independence Test

data:  VV4 by Gruppe (A, B)
Z = 3.072, p-value = 0.001061
alternative hypothesis: greater


Zeitvergleich Gruppe A:

Asymptotic Wilcoxon-Signed-Rank Test (zeros handled a la
Wilcoxon)

data:  y by
 x (neg, pos)
 stratified by block
Z = 2.358, p-value = 0.009198
alternative hypothesis: true mu is greater than 0


Zeitvergleich Gruppe B:

Asymptotic Wilcoxon-Signed-Rank Test (zeros handled a la
Wilcoxon)
```

```
data:  y by
 x (neg, pos)
 stratified by block
Z = -3.108, p-value = 0.0009431
alternative hypothesis: true mu is less than 0
```

In beiden Subgruppen wird das Ergebnis der Gesamtanalyse reproduziert. Ob das Trauma an der dominanten Hand oder an der nicht-dominanten Hand geschehen ist hat keinen Einfluss auf die Handbarkeit.

4.1.2 Subgruppenanalyse: dominante Hand verletzt bzw. gesund und Hygienegefühl

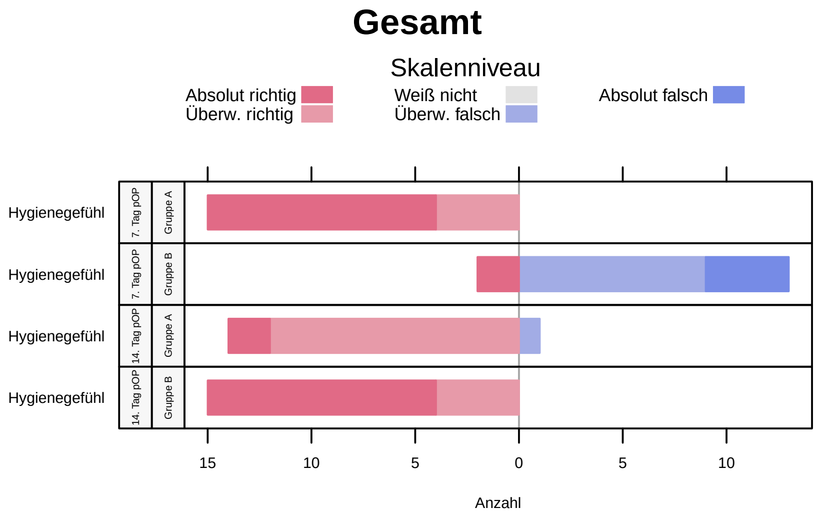

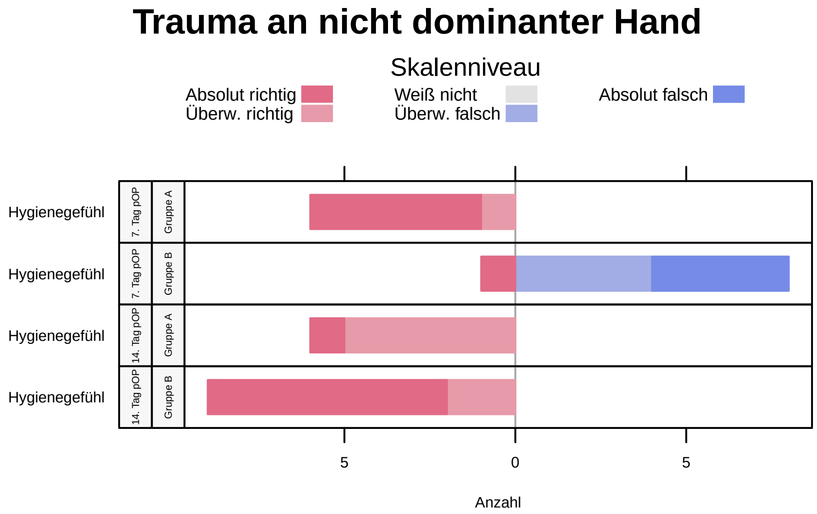

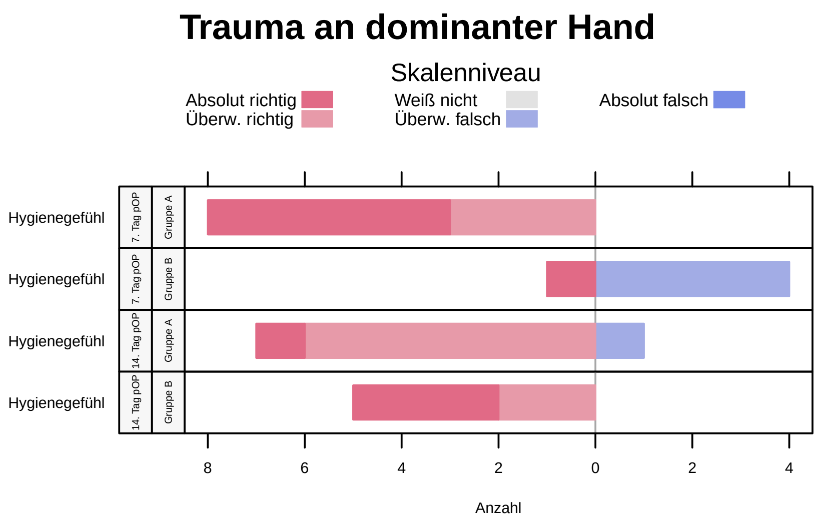


```
Trauma an nicht dominanter Hand
===============================
Gruppenvergleich 7. Tag pOP:

Asymptotic General Independence Test

data:  VV5 by Gruppe (A, B)
Z = -4.417, p-value = 5.007e-06
alternative hypothesis: less


Gruppenvergleich 14. Tag pOP:

Asymptotic General Independence Test

data:  VV5 by Gruppe (A, B)
Z = 3.003, p-value = 0.001335
alternative hypothesis: greater


Zeitvergleich Gruppe A:

Asymptotic Wilcoxon-Signed-Rank Test (zeros handled a la
Wilcoxon)

data:  y by
 x (neg, pos)
 stratified by block
Z = 2.673, p-value = 0.003763
alternative hypothesis: true mu is greater than 0


Zeitvergleich Gruppe B:

Asymptotic Wilcoxon-Signed-Rank Test (zeros handled a la
Wilcoxon)

data:  y by
 x (neg, pos)
 stratified by block
```

```
Z = -3.244, p-value = 0.0005897
alternative hypothesis: true mu is less than 0
```

```
Trauma an dominanter Hand
=========================
Gruppenvergleich 7. Tag pOP:

Asymptotic General Independence Test

data:  VV5 by Gruppe (A, B)
Z = -4.417, p-value = 5.007e-06
alternative hypothesis: less


Gruppenvergleich 14. Tag pOP:

Asymptotic General Independence Test

data:  VV5 by Gruppe (A, B)
Z = 3.003, p-value = 0.001335
alternative hypothesis: greater


Zeitvergleich Gruppe A:

Asymptotic Wilcoxon-Signed-Rank Test (zeros handled a la
Wilcoxon)

data:  y by
 x (neg, pos)
 stratified by block
Z = 2.673, p-value = 0.003763
alternative hypothesis: true mu is greater than 0


Zeitvergleich Gruppe B:

Asymptotic Wilcoxon-Signed-Rank Test (zeros handled a la
Wilcoxon)

data:  y by
 x (neg, pos)
 stratified by block
Z = -3.244, p-value = 0.0005897
alternative hypothesis: true mu is less than 0
```

In beiden Subgruppen wird das Ergebnis der Gesamtanalyse reproduziert. Ob das Trauma an der dominanten Hand oder an der nicht-dominanten Hand geschehen ist hat keinen Einfluss auf die Hygiene.

4.2 Subgruppenanalyse: Alter

4.2.1 Subgruppenanalyse: Alter und Handbarkeit

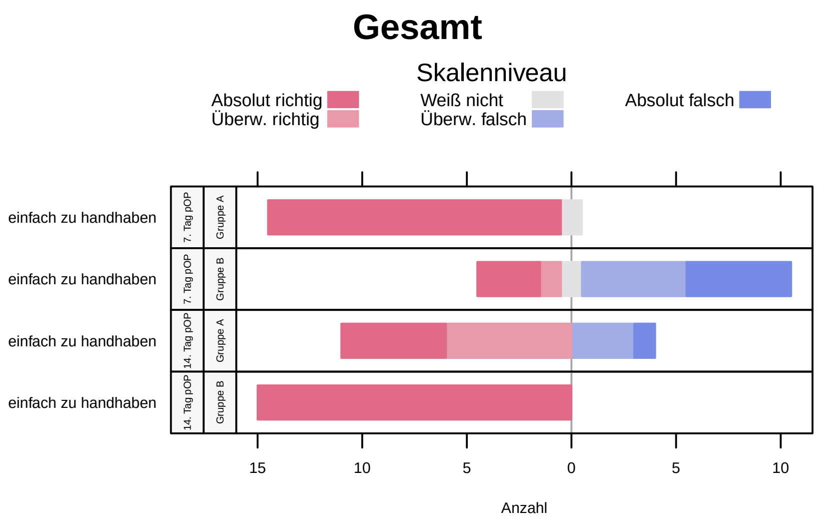

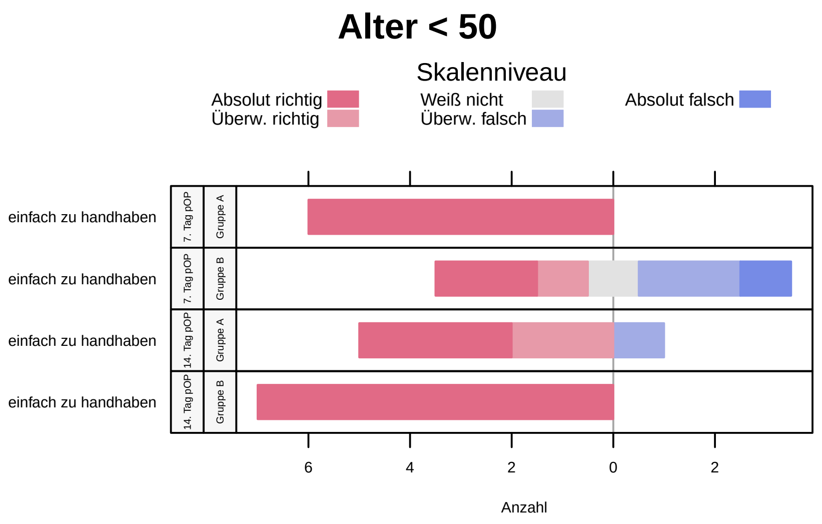

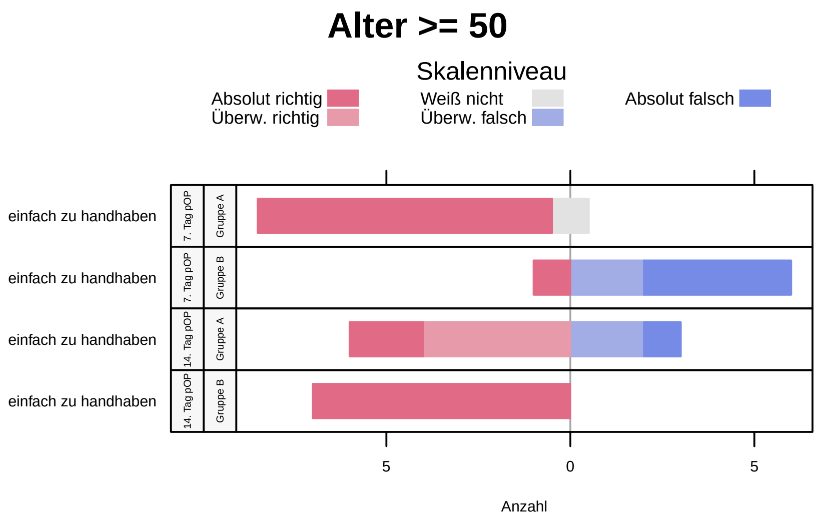


```
Alter < 50
===============================
Gruppenvergleich 7. Tag pOP:

Asymptotic General Independence Test
```

```
data:  VV4 by Gruppe (A, B)
Z = -2.268, p-value = 0.01167
alternative hypothesis: less


Gruppenvergleich 14. Tag pOP:

Asymptotic General Independence Test

data:  VV4 by Gruppe (A, B)
Z = 1.722, p-value = 0.04251
alternative hypothesis: greater


Zeitvergleich Gruppe A:

Asymptotic Wilcoxon-Signed-Rank Test (zeros handled a la
Wilcoxon)

data:  y by
 x (neg, pos)
 stratified by block
Z = 1.633, p-value = 0.05124
alternative hypothesis: true mu is greater than 0


Zeitvergleich Gruppe B:

Asymptotic Wilcoxon-Signed-Rank Test (zeros handled a la
Wilcoxon)

data:  y by
 x (neg, pos)
 stratified by block
Z = -2.032, p-value = 0.02108
alternative hypothesis: true mu is less than 0
```

```
Alter >= 50
=========================
Gruppenvergleich 7. Tag pOP:

Asymptotic General Independence Test

data:  VV4 by Gruppe (A, B)
Z = -3.174, p-value = 0.000751
alternative hypothesis: less


Gruppenvergleich 14. Tag pOP:

Asymptotic General Independence Test

data:  VV4 by Gruppe (A, B)
Z = 3.072, p-value = 0.001061
alternative hypothesis: greater
```

```
Zeitvergleich Gruppe A:

Asymptotic Wilcoxon-Signed-Rank Test (zeros handled a la
Wilcoxon)

data:  y by
 x (neg, pos)
 stratified by block
Z = 1.845, p-value = 0.03249
alternative hypothesis: true mu is greater than 0


Zeitvergleich Gruppe B:

Asymptotic Wilcoxon-Signed-Rank Test (zeros handled a la
Wilcoxon)

data:  y by
 x (neg, pos)
 stratified by block
Z = -2.271, p-value = 0.01157
alternative hypothesis: true mu is less than 0

Vergleich 7. Tag pOP und Gruppe B: Altersvergleich:

Wilcoxon rank sum test with continuity correction

data:  VV4 by AlterB
W = 14, p-value = 0.1036
alternative hypothesis: true location shift is not equal to 0

Vergleich 14. Tag pOP und Gruppe A: Altersvergleich:

Wilcoxon rank sum test with continuity correction

data:  VV4 by AlterB
W = 18, p-value = 0.2896
alternative hypothesis: true location shift is not equal to 0
```

In beiden Subgruppen wird das Ergebnis der Gesamtanalyse reproduziert.

4.2.2 Subgruppenanalyse: Alter und Hygienegefühl

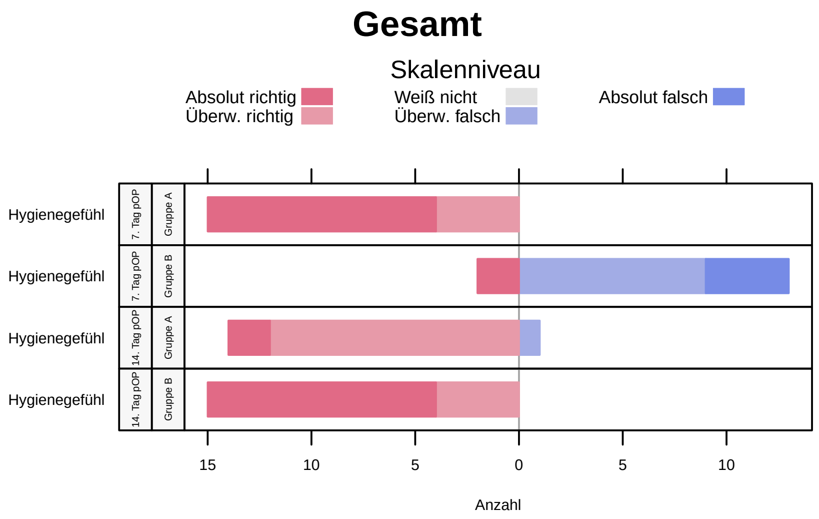

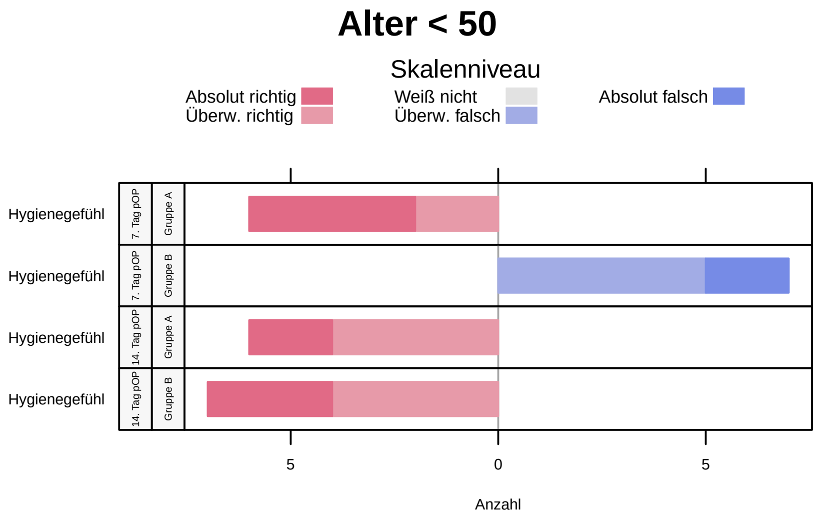

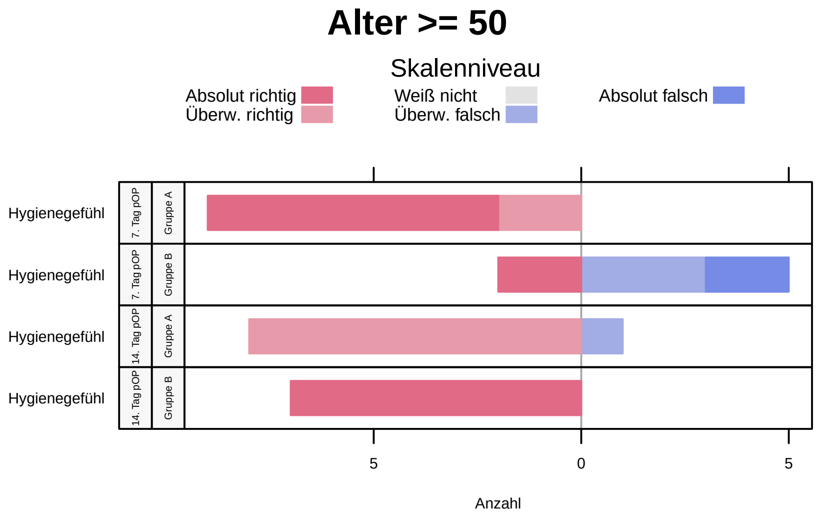


```
Alter < 50
===============================
Gruppenvergleich 7. Tag pOP:

Asymptotic General Independence Test
```

```
data:  VV5 by Gruppe (A, B)
Z = -3.306, p-value = 0.0004736
alternative hypothesis: less


Gruppenvergleich 14. Tag pOP:

Asymptotic General Independence Test

data:  VV5 by Gruppe (A, B)
Z = 0.3381, p-value = 0.3677
alternative hypothesis: greater


Zeitvergleich Gruppe A:

Asymptotic Wilcoxon-Signed-Rank Test (zeros handled a la
Wilcoxon)

data:  y by
 x (neg, pos)
 stratified by block
Z = 1, p-value = 0.1587
alternative hypothesis: true mu is greater than 0


Zeitvergleich Gruppe B:

Asymptotic Wilcoxon-Signed-Rank Test

data:  y by
 x (neg, pos)
 stratified by block
Z = -2.401, p-value = 0.008176
alternative hypothesis: true mu is less than 0

Vergleich 7. Tag pOP und Gruppe B: Altersvergleich

Wilcoxon rank sum test with continuity correction

data:  VV5 by AlterB
W = 22, p-value = 0.4671
alternative hypothesis: true location shift is not equal to 0

Vergleich 14. Tag pOP und Gruppe A: Altersvergleich

Wilcoxon rank sum test with continuity correction

data:  VV5 by AlterB
W = 38, p-value = 0.07635
alternative hypothesis: true location shift is not equal to 0
```

```
Alter >= 50
========================
Gruppenvergleich 7. Tag pOP:

Asymptotic General Independence Test
```

```
data:  VV5 by Gruppe (A, B)
Z = -2.735, p-value = 0.003119
alternative hypothesis: less


Gruppenvergleich 14. Tag pOP:

Asymptotic General Independence Test

data:  VV5 by Gruppe (A, B)
Z = 3.058, p-value = 0.001116
alternative hypothesis: greater


Zeitvergleich Gruppe A:

Asymptotic Wilcoxon-Signed-Rank Test (zeros handled a la
Wilcoxon)

data:  y by
 x (neg, pos)
 stratified by block
Z = 2.53, p-value = 0.005706
alternative hypothesis: true mu is greater than 0


Zeitvergleich Gruppe B:

Asymptotic Wilcoxon-Signed-Rank Test (zeros handled a la
Wilcoxon)

data:  y by
 x (neg, pos)
 stratified by block
Z = -2.07, p-value = 0.01922
alternative hypothesis: true mu is less than 0
```

4.3 Subgruppenanalyse: Frakturklassifikation

4.3.1 Subgruppenanalyse: Frakturklassifikation und Handbarkeit

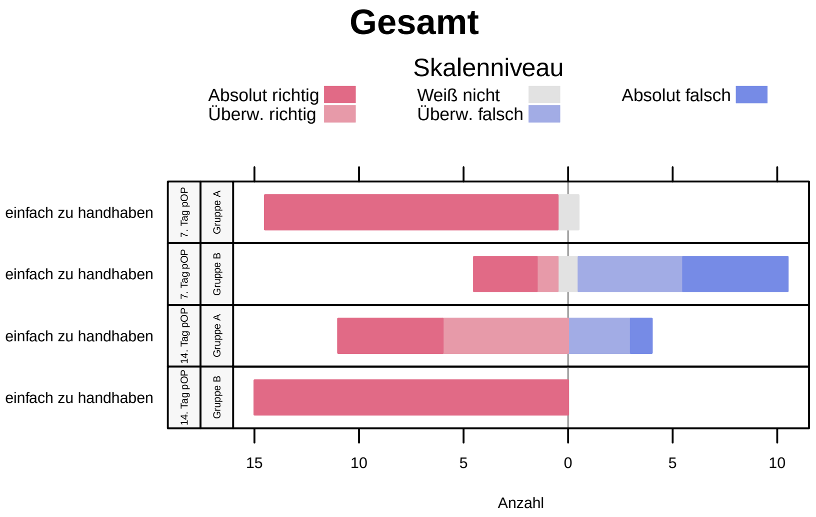
Gesamt
Skalenniveau
Absolut richtig
Überw. richtig
Weiß nicht
Überw. falsch
Absolut falsch
einfach zu handhaben
7. Tag pOP
14. Tag pOP
Gruppe A
Gruppe B
15
10
5
0
Anzahl

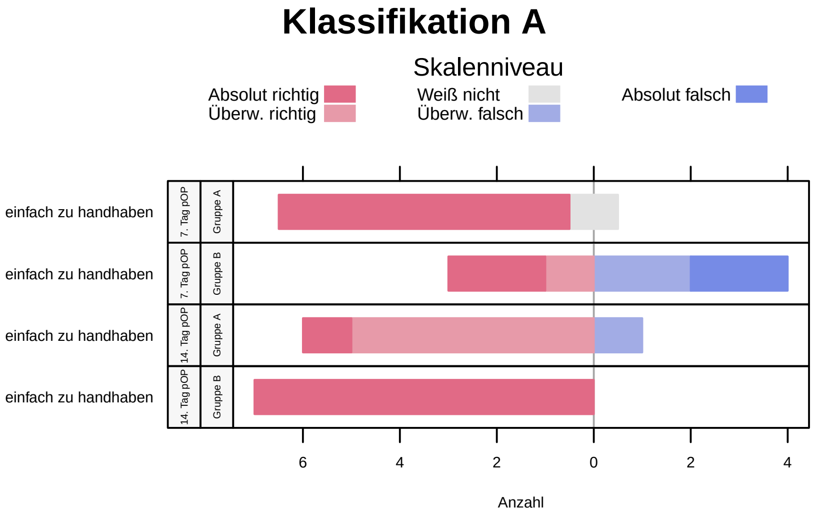
Klassifikation A
Skalenniveau
Absolut richtig
Überw. richtig
Weiß nicht
Überw. falsch
Absolut falsch
einfach zu handhaben
7. Tag pOP
14. Tag pOP
Gruppe A
Gruppe B
6
4
2
0
Anzahl

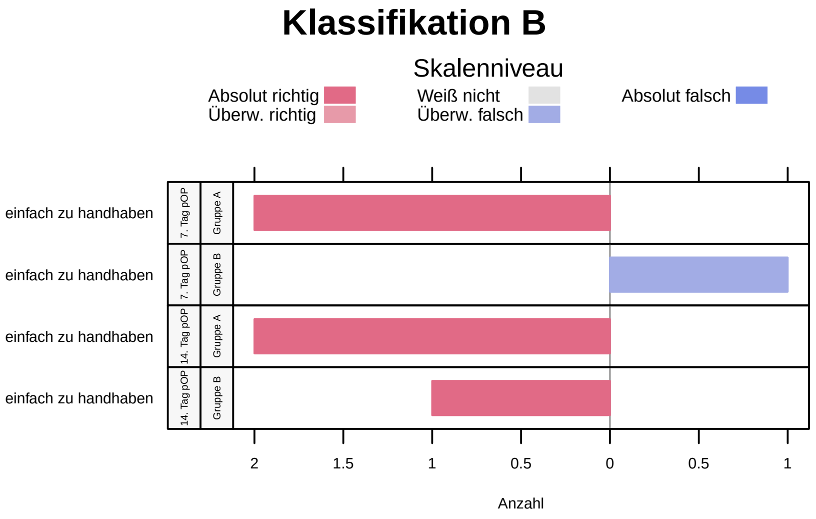
Klassifikation B
Skalenniveau
Absolut richtig
Überw. richtig
Weiß nicht
Überw. falsch
Absolut falsch
einfach zu handhaben
7. Tag pOP
14. Tag pOP
Gruppe A
Gruppe B
2
1.5
1
0.5
0
Anzahl

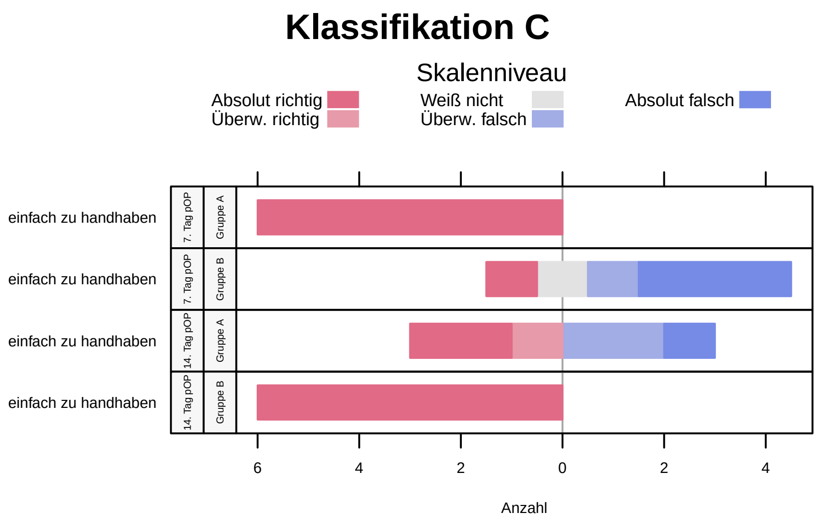


```
Klassifikation A
================================
Gruppenvergleich 7. Tag pOP:

Asymptotic General Independence Test

data:  VV4 by Gruppe (A, B)
Z = -3.941, p-value = 4.059e-05
alternative hypothesis: less


Gruppenvergleich 14. Tag pOP:

Asymptotic General Independence Test

data:  VV4 by Gruppe (A, B)
Z = 3.072, p-value = 0.001061
alternative hypothesis: greater


Zeitvergleich Gruppe A:

Asymptotic Wilcoxon-Signed-Rank Test (zeros handled a la
Wilcoxon)

data:  y by
 x (neg, pos)
 stratified by block
Z = 2.358, p-value = 0.009198
alternative hypothesis: true mu is greater than 0


Zeitvergleich Gruppe B:

Asymptotic Wilcoxon-Signed-Rank Test (zeros handled a la
Wilcoxon)

data:  y by
 x (neg, pos)
 stratified by block
```

```
Z = -3.108, p-value = 0.0009431
alternative hypothesis: true mu is less than 0
```

```
Klassifikation B
================================
Gruppenvergleich 7. Tag pOP:

Asymptotic General Independence Test

data:  VV4 by Gruppe (A, B)
Z = -3.941, p-value = 4.059e-05
alternative hypothesis: less


Gruppenvergleich 14. Tag pOP:

Asymptotic General Independence Test

data:  VV4 by Gruppe (A, B)
Z = 3.072, p-value = 0.001061
alternative hypothesis: greater


Zeitvergleich Gruppe A:

Asymptotic Wilcoxon-Signed-Rank Test (zeros handled a la
Wilcoxon)

data:  y by
 x (neg, pos)
 stratified by block
Z = 2.358, p-value = 0.009198
alternative hypothesis: true mu is greater than 0


Zeitvergleich Gruppe B:

Asymptotic Wilcoxon-Signed-Rank Test (zeros handled a la
Wilcoxon)

data:  y by
 x (neg, pos)
 stratified by block
Z = -3.108, p-value = 0.0009431
alternative hypothesis: true mu is less than 0
```

```
Klassifikation C
================================
Gruppenvergleich 7. Tag pOP:

Asymptotic General Independence Test

data:  VV4 by Gruppe (A, B)
Z = -3.941, p-value = 4.059e-05
alternative hypothesis: less
```

```
Gruppenvergleich 14. Tag pOP:

Asymptotic General Independence Test

data:  VV4 by Gruppe (A, B)
Z = 3.072, p-value = 0.001061
alternative hypothesis: greater


Zeitvergleich Gruppe A:

Asymptotic Wilcoxon-Signed-Rank Test (zeros handled a la
Wilcoxon)

data:  y by
 x (neg, pos)
 stratified by block
Z = 2.358, p-value = 0.009198
alternative hypothesis: true mu is greater than 0


Zeitvergleich Gruppe B:

Asymptotic Wilcoxon-Signed-Rank Test (zeros handled a la
Wilcoxon)

data:  y by
 x (neg, pos)
 stratified by block
Z = -3.108, p-value = 0.0009431
alternative hypothesis: true mu is less than 0
```

```
Vergleich 7. Tag pOP und Gruppe B: Frakturvergleich A vs C

Wilcoxon rank sum test with continuity correction

data:  VV5 by AO.Klassif.
W = 29, p-value = 0.5137
alternative hypothesis: true location shift is not equal to 0

Vergleich 14. Tag pOP und Gruppe A: Frakturvergleich A vs C

Wilcoxon rank sum test with continuity correction

data:  VV5 by AO.Klassif.
W = 24.5, p-value = 0.5612
alternative hypothesis: true location shift is not equal to 0
```

Der grafische Anschein eines Unterschieds bestätigt sich nicht.

4.3.2 Subgruppenanalyse: Frakturklassifikation und Hygienegefühl

Gesamt

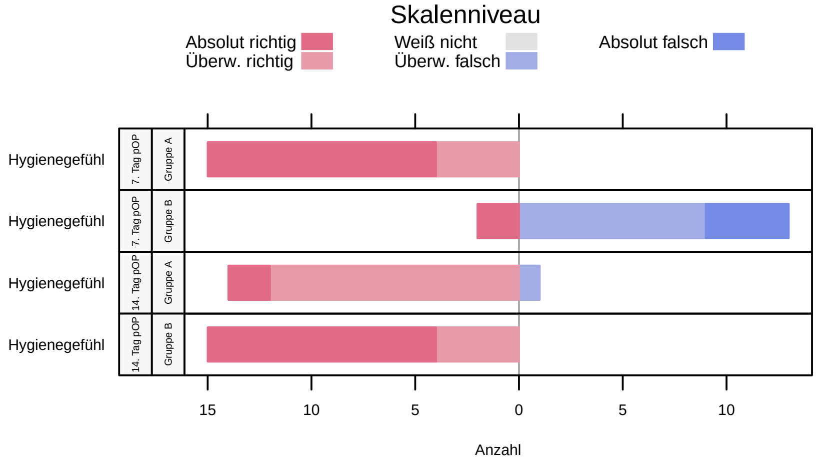

Frakturklassifikation A

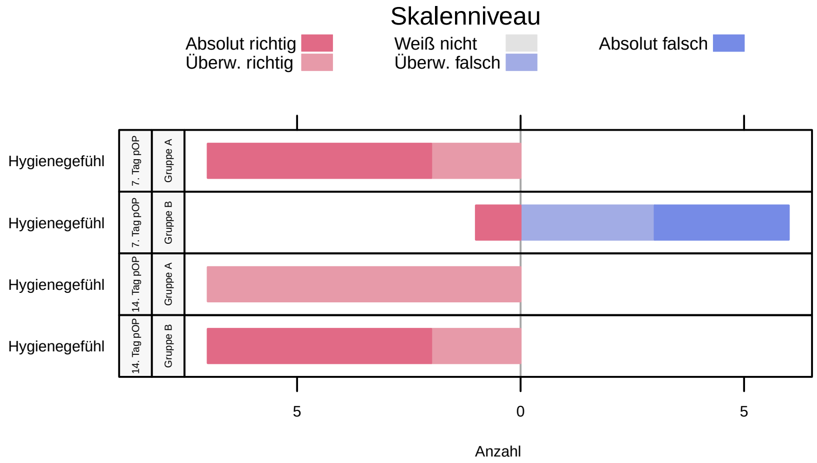

Frakturklassifikation B

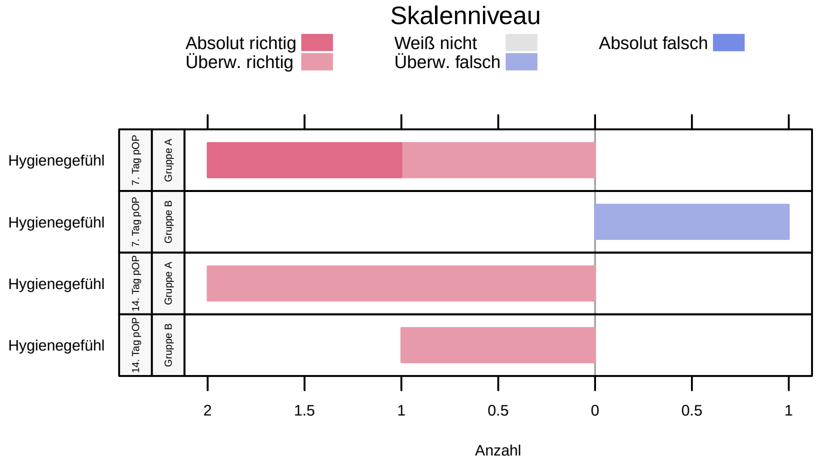

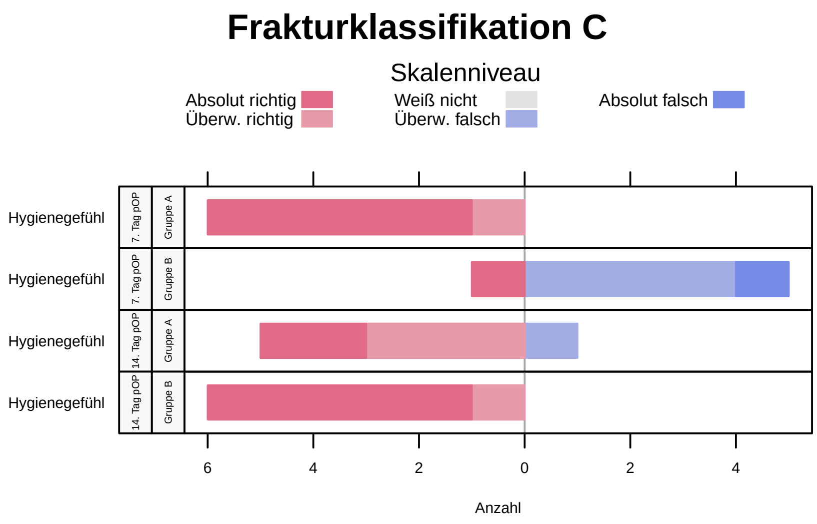


```
================================
Gruppenvergleich 7. Tag pOP:

Asymptotic General Independence Test

data:  VV5 by Gruppe (A, B)
Z = -4.417, p-value = 5.007e-06
alternative hypothesis: less


Gruppenvergleich 14. Tag pOP:

Asymptotic General Independence Test

data:  VV5 by Gruppe (A, B)
Z = 3.003, p-value = 0.001335
alternative hypothesis: greater


Zeitvergleich Gruppe A:

Asymptotic Wilcoxon-Signed-Rank Test (zeros handled a la
Wilcoxon)

data:  y by
 x (neg, pos)
 stratified by block
Z = 2.673, p-value = 0.003763
alternative hypothesis: true mu is greater than 0


Zeitvergleich Gruppe B:

Asymptotic Wilcoxon-Signed-Rank Test (zeros handled a la
Wilcoxon)

data:  y by
 x (neg, pos)
 stratified by block
Z = -3.244, p-value = 0.0005897
```

```
alternative hypothesis: true mu is less than 0
```

```
================================
Gruppenvergleich 7. Tag pOP:

Asymptotic General Independence Test

data:  VV5 by Gruppe (A, B)
Z = -4.417, p-value = 5.007e-06
alternative hypothesis: less


Gruppenvergleich 14. Tag pOP:

Asymptotic General Independence Test

data:  VV5 by Gruppe (A, B)
Z = 3.003, p-value = 0.001335
alternative hypothesis: greater


Zeitvergleich Gruppe A:

Asymptotic Wilcoxon-Signed-Rank Test (zeros handled a la
Wilcoxon)

data:  y by
 x (neg, pos)
 stratified by block
Z = 2.673, p-value = 0.003763
alternative hypothesis: true mu is greater than 0


Zeitvergleich Gruppe B:

Asymptotic Wilcoxon-Signed-Rank Test (zeros handled a la
Wilcoxon)

data:  y by
 x (neg, pos)
 stratified by block
Z = -3.244, p-value = 0.0005897
alternative hypothesis: true mu is less than 0
```

```
================================
Gruppenvergleich 7. Tag pOP:

Asymptotic General Independence Test

data:  VV5 by Gruppe (A, B)
Z = -4.417, p-value = 5.007e-06
alternative hypothesis: less


Gruppenvergleich 14. Tag pOP:

Asymptotic General Independence Test
```

```
data:  VV5 by Gruppe (A, B)
Z = 3.003, p-value = 0.001335
alternative hypothesis: greater


Zeitvergleich Gruppe A:

Asymptotic Wilcoxon-Signed-Rank Test (zeros handled a la
Wilcoxon)

data:  y by
 x (neg, pos)
 stratified by block
Z = 2.673, p-value = 0.003763
alternative hypothesis: true mu is greater than 0


Zeitvergleich Gruppe B:

Asymptotic Wilcoxon-Signed-Rank Test (zeros handled a la
Wilcoxon)

data:  y by
 x (neg, pos)
 stratified by block
Z = -3.244, p-value = 0.0005897
alternative hypothesis: true mu is less than 0
```

4.4 SF-36 Fragebogen

Die Items wurden entsprechend umkodiert, so dass niedrige Werte mit negativen Eigenschaften verbunden sind und hohe mit positiven. Die Items wurd zu den 8 Skalen zugeordnet. Für jede Skala wurde ein Skalenwert durch Addition der Items einer Skala berechnet in eine Skala von 0 bis 100 transformiert. Die Analyse wurde separat für die 8 Skalen durchgeführt. Die Frage 2 des SF-36 wurde nicht berücksichtigt. Es wurde keine Normierung bzgl. eine Normbevölkerung durchgeführt.

4.4.1 Analyse der 8 Dimensionen

Zunächst werden Boxplots erzeugt, in welchen die Gruppe je Woche verglichen werden können. Für die Gruppenvergleiche je Zeitpunkt wird der Mann-Whitney-Test eingesetzt.
Für die Innergruppen-Analyse für den Vergleich zwischen den Zeitpunkten wird zum einen der Wilcoxon-Vorzeichen-Rang-Test verwendet. Bei diesem Test wird die Differenz jedes Probanden über die Zeitpunkte berechnet und getestet, ob sich diese Differenzen je Gruppe von Null unterscheidet. Anders Formuliert: die Nullhypothese ist, dass die Probanden Ihre Einstellung in der jeweiligen Skala nicht ändern. diese Differenzen sind zudem in Boxplots geplottet. Interpretation: positive Werte bedeuten eine Verbesserung vom 7. Tag pOP zum 14. Tag pOP, negative Werte eine Verschlechterung.

Körperliche Funktionsfähigkeit

```


Mann-Whitney-Test für Woche 1 (Gruppenvergleich A zu B):
Wilcoxon rank sum test with continuity correction

data:  phys_func by Gruppe
W = 126.5, p-value = 0.3552
alternative hypothesis: true location shift is not equal to 0
```

```
Mann-Whitney-Test für Woche 2 (Gruppenvergleich A zu B):
Wilcoxon rank sum test with continuity correction

data:  phys_func by Gruppe
W = 102.5, p-value = 0.9298
alternative hypothesis: true location shift is not equal to 0



Wilcoxon-Vorzeichen-Rang-Test für Gruppe A:
Asymptotic Wilcoxon-Signed-Rank Test

data:  y by
 x (neg, pos)
 stratified by block
Z = -0.4862, p-value = 0.6268
alternative hypothesis: true mu is not equal to 0



Wilcoxon-Vorzeichen-Rang-Test für Gruppe B:
Asymptotic Wilcoxon-Signed-Rank Test

data:  y by
 x (neg, pos)
 stratified by block
Z = -1.605, p-value = 0.1085
alternative hypothesis: true mu is not equal to 0
```

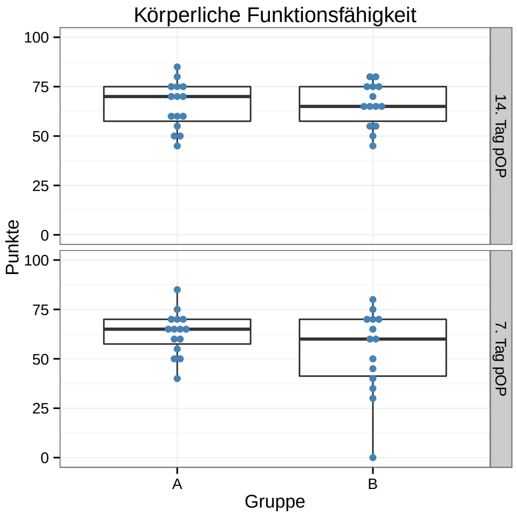

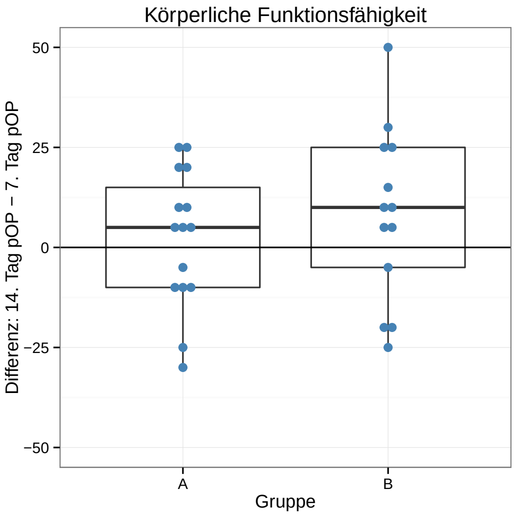

Die körperlichen Funktionsfähigkeit wird generell als hoch empfunden. Bei der körperlichen Funktionsfähigkeit unterscheiden sich die Gruppen zu keinem Zeitpunkt voneinander. Auch kann man keine zeitliche Änderung (also eine Änderung der körperlichen Funktionsfähigkeit beim Wechsel von Gips auf bwVacoHand, vice versa).

Körperliche Rollenfunktion

```
Mann-Whitney-Test für Woche 1 (Gruppenvergleich A zu B):
```

```
Wilcoxon rank sum test with continuity correction

data:  role_phys_he by Gruppe
W = 98, p-value = 0.5916
alternative hypothesis: true location shift is not equal to 0



Mann-Whitney-Test für Woche 2 (Gruppenvergleich A zu B):
Wilcoxon rank sum test with continuity correction

data:  role_phys_he by Gruppe
W = 98, p-value = 0.5916
alternative hypothesis: true location shift is not equal to 0



Wilcoxon-Vorzeichen-Rang-Test für Gruppe A:
Asymptotic Wilcoxon-Signed-Rank Test (zeros handled a la
Wilcoxon)

data:  y by
 x (neg, pos)
 stratified by block
Z = 0, p-value = 1
alternative hypothesis: true mu is not equal to 0



Wilcoxon-Vorzeichen-Rang-Test für Gruppe B:
Asymptotic Wilcoxon-Signed-Rank Test (zeros handled a la
Wilcoxon)

data:  y by
 x (neg, pos)
 stratified by block
Z = 0, p-value = 1
alternative hypothesis: true mu is not equal to 0
```

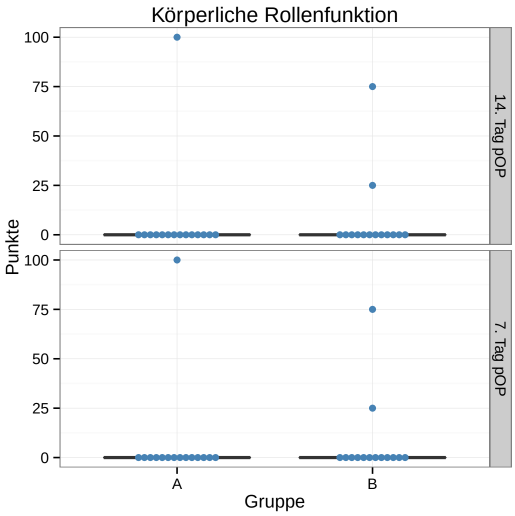

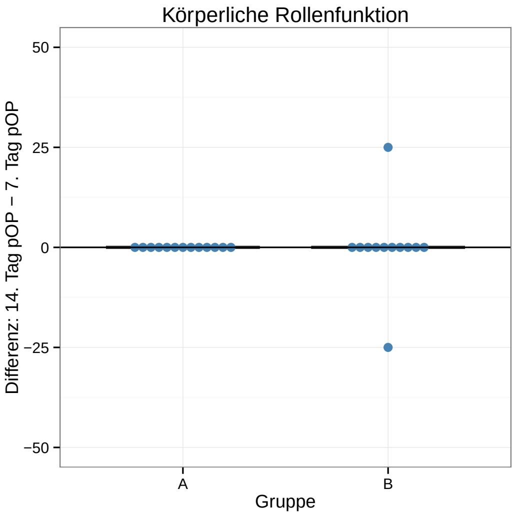

Die Körperliche Rollenfunktion der Probanden war in den vergangenen vier Wochen stark eingeschränkt. Es wurden bei keiner Analyse in dieser Skala Unterschiede gefunden.

Emotionale Rollenfunktion

```
Mann-Whitney-Test für Woche 1 (Gruppenvergleich A zu B):
Wilcoxon rank sum test with continuity correction

data:  role_emo_prob by Gruppe
W = 83.5, p-value = 0.2616
alternative hypothesis: true location shift is not equal to 0



Mann-Whitney-Test für Woche 2 (Gruppenvergleich A zu B):
Wilcoxon rank sum test with continuity correction

data:  role_emo_prob by Gruppe
W = 80.5, p-value = 0.2266
alternative hypothesis: true location shift is not equal to 0



Wilcoxon-Vorzeichen-Rang-Test für Gruppe A:
Asymptotic Wilcoxon-Signed-Rank Test (zeros handled a la
Wilcoxon)

data:  y by
 x (neg, pos)
 stratified by block
Z = 0.4243, p-value = 0.6714
alternative hypothesis: true mu is not equal to 0



Wilcoxon-Vorzeichen-Rang-Test für Gruppe B:
Asymptotic Wilcoxon-Signed-Rank Test (zeros handled a la
Wilcoxon)

data:  y by
 x (neg, pos)
 stratified by block
Z = 0.1054, p-value = 0.9161
alternative hypothesis: true mu is not equal to 0
```

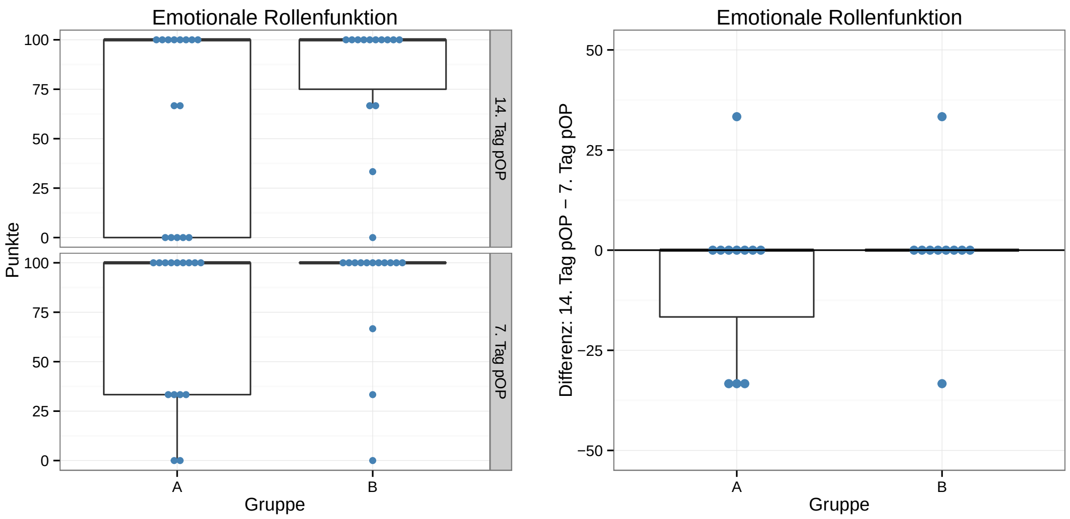

Die Emotionale Rollenfunktion war bei den Probanden überwiegend sehr positiv. Es wurden bei keiner Analyse in dieser Skala Unterschiede gefunden.

Vitalität

```
Mann-Whitney-Test für Woche 1 (Gruppenvergleich A zu B):
Wilcoxon rank sum test with continuity correction

data:  energ by Gruppe
W = 119, p-value = 0.5531
alternative hypothesis: true location shift is not equal to 0



Mann-Whitney-Test für Woche 2 (Gruppenvergleich A zu B):
Wilcoxon rank sum test with continuity correction

data:  energ by Gruppe
W = 101, p-value = 0.8777
alternative hypothesis: true location shift is not equal to 0



Wilcoxon-Vorzeichen-Rang-Test für Gruppe A:
Asymptotic Wilcoxon-Signed-Rank Test (zeros handled a la
Wilcoxon)

data:  y by
 x (neg, pos)
 stratified by block
Z = 0.5913, p-value = 0.5543
alternative hypothesis: true mu is not equal to 0



Wilcoxon-Vorzeichen-Rang-Test für Gruppe B:
Asymptotic Wilcoxon-Signed-Rank Test

data:  y by
```

```
 x (neg, pos)
 stratified by block
Z = -1.014, p-value = 0.3104
alternative hypothesis: true mu is not equal to 0
```

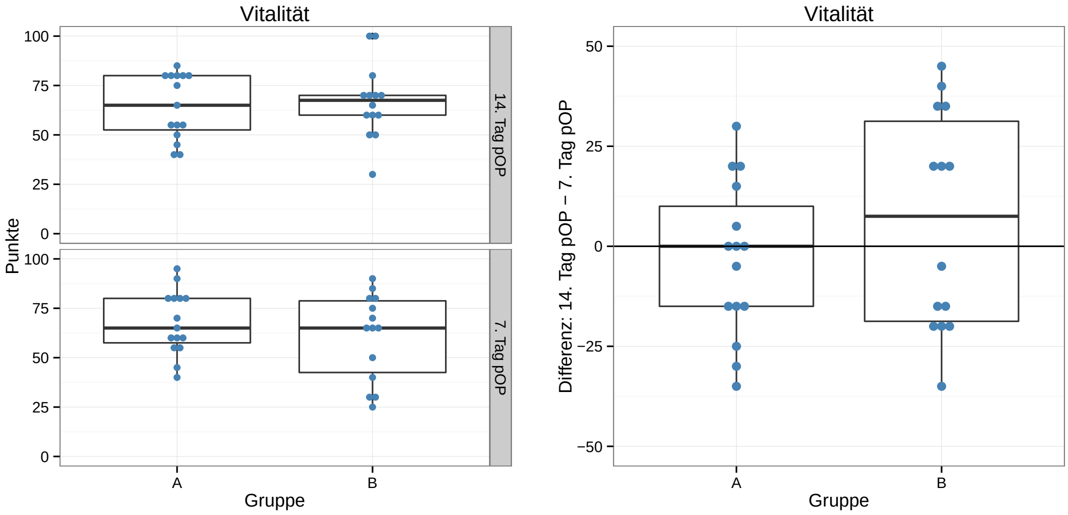

Die Vitalität wurde von den Probanden mehrheitlich als nicht eingeschränkt empfunden. In Gruppe B (Gips -> VacoHand) tritt ein Grenzeffekt auf. Einige Probanden empfinden bei der VacoHand eine bessere Vitalität als beim Gips (grafische Interpretation), statistische wird dies jedoch nicht bestätigt. Die bessere Vitalität bei diesen Patienten könnte auch von der zeitlichen Dauer herstammen. In Gruppe A (VacoHand -> Gips) ist weder grafisch noch statistische ein Effekt sichtbar.

Psychisches Wohlbefinden

```
Mann-Whitney-Test für Woche 1 (Gruppenvergleich A zu B):
Wilcoxon rank sum test with continuity correction

data:  emo_well_being by Gruppe
W = 104.5, p-value = 1
alternative hypothesis: true location shift is not equal to 0



Mann-Whitney-Test für Woche 2 (Gruppenvergleich A zu B):
Wilcoxon rank sum test with continuity correction

data:  emo_well_being by Gruppe
W = 108.5, p-value = 0.8949
alternative hypothesis: true location shift is not equal to 0



Wilcoxon-Vorzeichen-Rang-Test für Gruppe A:
Asymptotic Wilcoxon-Signed-Rank Test (zeros handled a la
Wilcoxon)

data:  y by
 x (neg, pos)
 stratified by block
Z = -1.227, p-value = 0.2199
```

```
alternative hypothesis: true mu is not equal to 0



Wilcoxon-Vorzeichen-Rang-Test für Gruppe B:
Asymptotic Wilcoxon-Signed-Rank Test (zeros handled a la
Wilcoxon)

data:  y by
 x (neg, pos)
 stratified by block
Z = -1.122, p-value = 0.2619
alternative hypothesis: true mu is not equal to 0
```

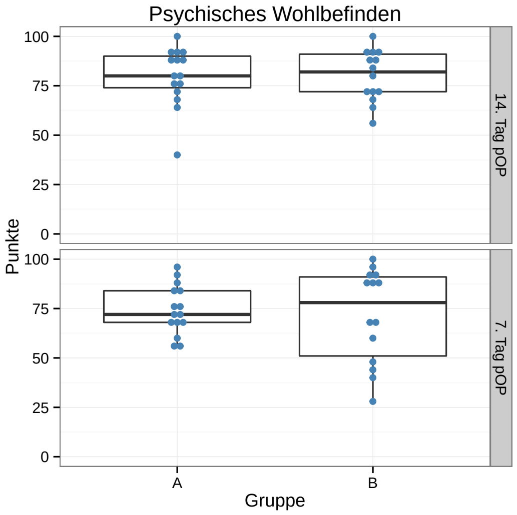

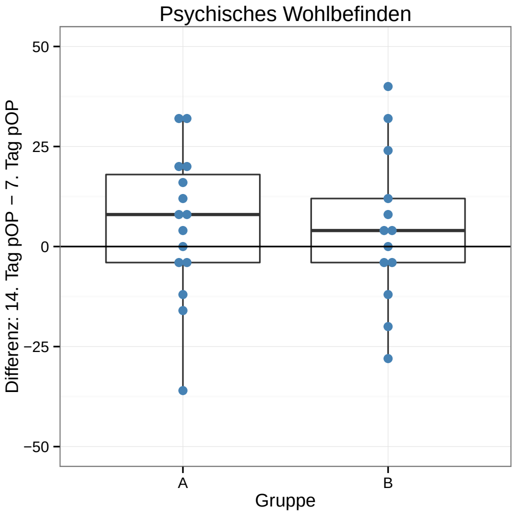

Das psychische Wohlbefinden wurde von den Probanden mehrheitlich als hoch empfunden. Es wurden bei keiner Analyse in dieser Skala Unterschiede gefunden.

Soziale Funktionsfähigkeit

```
Mann-Whitney-Test für Woche 1 (Gruppenvergleich A zu B):
Wilcoxon rank sum test with continuity correction

data:  soc_func by Gruppe
W = 97, p-value = 0.7403
alternative hypothesis: true location shift is not equal to 0



Mann-Whitney-Test für Woche 2 (Gruppenvergleich A zu B):
Wilcoxon rank sum test with continuity correction

data:  soc_func by Gruppe
W = 76.5, p-value = 0.218
alternative hypothesis: true location shift is not equal to 0



Wilcoxon-Vorzeichen-Rang-Test für Gruppe A:
Asymptotic Wilcoxon-Signed-Rank Test (zeros handled a la
```

```
Wilcoxon)

data:  y by
 x (neg, pos)
 stratified by block
Z = 0.733, p-value = 0.4635
alternative hypothesis: true mu is not equal to 0



Wilcoxon-Vorzeichen-Rang-Test für Gruppe B:
Asymptotic Wilcoxon-Signed-Rank Test (zeros handled a la
Wilcoxon)

data:  y by
 x (neg, pos)
 stratified by block
Z = -0.6695, p-value = 0.5032
alternative hypothesis: true mu is not equal to 0
```

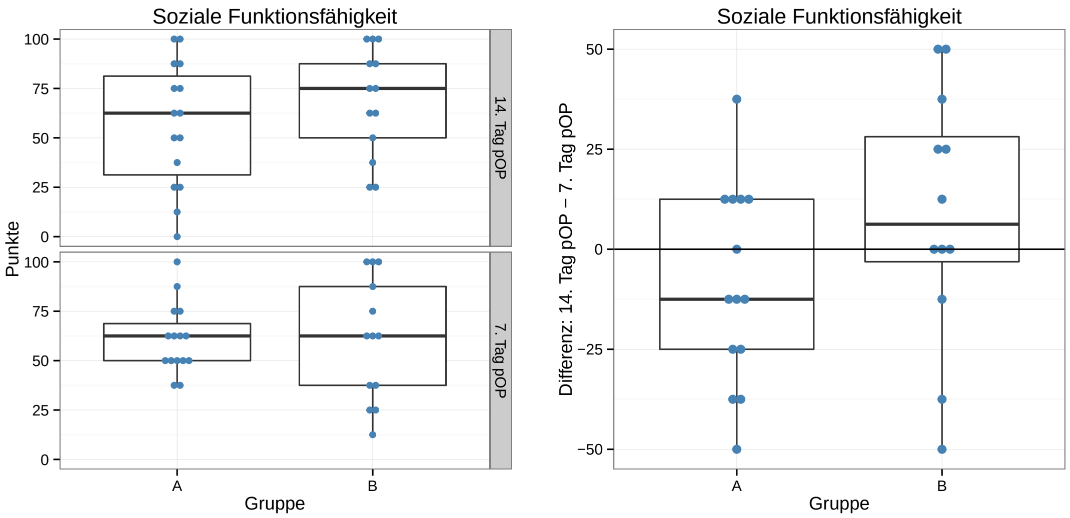

Grafische Interpretation: In Gruppe B empfinden einige Probanden durch den Wechsel von Gips auf die VacoHand eine Steigerung der sozialen Funktionsfähigkeit. Einige Probanden in Gruppe A hingegen, empfinden eine Verschlechterung der soziale Funktionsfähigkeit. Statistische kann dieser Effekt jedoch nicht nachgewiesen werden.

Körperliche Schmerzen

```
Mann-Whitney-Test für Woche 1 (Gruppenvergleich A zu B):
Wilcoxon rank sum test with continuity correction

data:  pain by Gruppe
W = 107.5, p-value = 0.9295
alternative hypothesis: true location shift is not equal to 0



Mann-Whitney-Test für Woche 2 (Gruppenvergleich A zu B):
Wilcoxon rank sum test with continuity correction
```

```
data:  pain by Gruppe
W = 89.5, p-value = 0.5059
alternative hypothesis: true location shift is not equal to 0



Wilcoxon-Vorzeichen-Rang-Test für Gruppe A:
Asymptotic Wilcoxon-Signed-Rank Test (zeros handled a la
Wilcoxon)

data:  y by
 x (neg, pos)
 stratified by block
Z = 1.743, p-value = 0.08139
alternative hypothesis: true mu is not equal to 0



Wilcoxon-Vorzeichen-Rang-Test für Gruppe B:
Asymptotic Wilcoxon-Signed-Rank Test (zeros handled a la
Wilcoxon)

data:  y by
 x (neg, pos)
 stratified by block
Z = 0.739, p-value = 0.4599
alternative hypothesis: true mu is not equal to 0
```

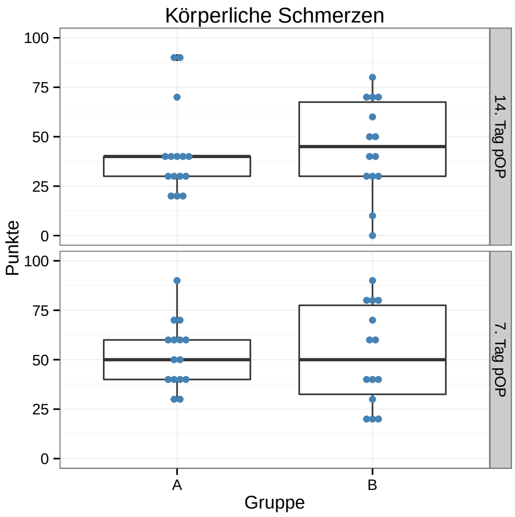

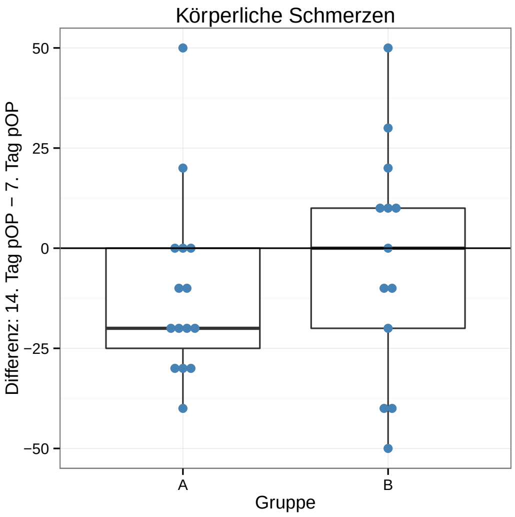

Der Median der körperliche Schmerzen liegt zum Zeitpunkt 1 bei beiden Gruppen bei etwa 40 Punkt und wächst zum Zeitpunkt 2 auf 50 Punkte an. Statistisch signifikant ist der Zuwachs, denn man grafisch erkennen kann, nicht.

Allgemeine Gesundheit

```
Mann-Whitney-Test für Woche 1 (Gruppenvergleich A zu B):
Wilcoxon rank sum test with continuity correction

data:  gen_health by Gruppe
W = 134, p-value = 0.2064
```

```
alternative hypothesis: true location shift is not equal to 0



Mann-Whitney-Test für Woche 2 (Gruppenvergleich A zu B):
Wilcoxon rank sum test with continuity correction

data:  gen_health by Gruppe
W = 72, p-value = 0.1534
alternative hypothesis: true location shift is not equal to 0



Wilcoxon-Vorzeichen-Rang-Test für Gruppe A:
Asymptotic Wilcoxon-Signed-Rank Test

data:  y by
 x (neg, pos)
 stratified by block
Z = 1.8, p-value = 0.07192
alternative hypothesis: true mu is not equal to 0



Wilcoxon-Vorzeichen-Rang-Test für Gruppe B:
Asymptotic Wilcoxon-Signed-Rank Test (zeros handled a la
Wilcoxon)

data:  y by
 x (neg, pos)
 stratified by block
Z = -0.8317, p-value = 0.4056
alternative hypothesis: true mu is not equal to 0
```

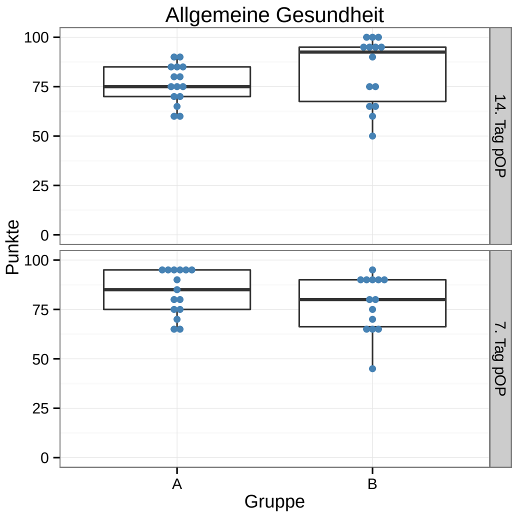

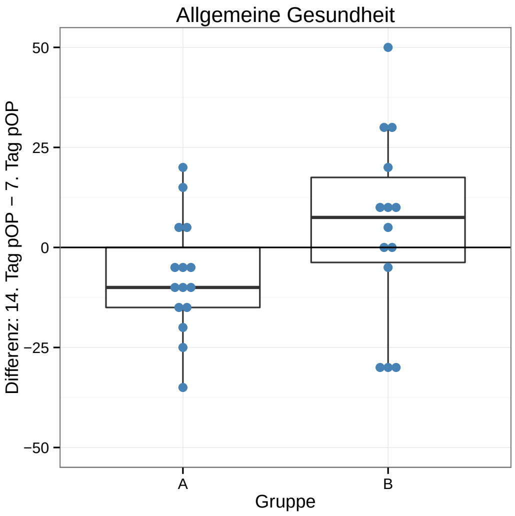

Die allgemeine Gesundheit wurde von den Probanden mehrheitlich als hoch empfunden. Es wurden bei keiner Analyse in dieser Skala Unterschiede gefunden.

5 Analyse: 1 Monat pOP und 3 Monate pOP

5.1 DASH-Fragebogen

Die Berechnung des Scores erolgt auf Analoge weise wie für den vollen Datensatz (Normierung auf eine Skala mit Werten in $0-100$). Die minimal erreichbare Punktezahl ist 30, die Maximale 150. Die daraus resultierende Bandbreite ist 120.
Ein kleiner Wert bedeuted keine Einschränkung, ein Hoher starke Einschränkungen.

5.2 Auswertung des DASH-Scores

```
d <- merge(dataDASH, data, by = c("Studienummer"))

Error: 'by' muss gültige Spalte(n) spezifizieren

ggplot(d) + geom_point(aes(x = Alter.in.J., y = score)) + facet_grid(Gruppe.x ~
    Woche)

Error: Objekt 'd' nicht gefunden
```

```
pdf
  2
Gruppenvergleich: Gruppe A zu Gruppe B
======================================
1 Monat pOP

Wilcoxon rank sum test with continuity correction

data:  score by Gruppe
W = 106, p-value = 0.9826
alternative hypothesis: true location shift is not equal to 0


3 Monate pOP

Wilcoxon rank sum test with continuity correction

data:  score by Gruppe
W = 90.5, p-value = 0.5403
alternative hypothesis: true location shift is not equal to 0
DASH-Score: 1 Monat pOP versus 3 Monate pOP, Gruppe A

Wilcoxon signed rank test with continuity correction

data:  dataDASH[dataDASH$Gruppe == "Gruppe A" & dataDASH$Woche == "1 Monat pOP",     ]$score - dataD
V = 119, p-value = 0.0008898
alternative hypothesis: true location is not equal to 0
DASH-Score: 1 Monat pOP versus 3 Monate pOP, Gruppe B

Wilcoxon signed rank test with continuity correction

data:  dataDASH[dataDASH$Gruppe == "Gruppe B" & dataDASH$Woche == "1 Monat pOP",     ]$score - dataD
V = 103, p-value = 0.001692
alternative hypothesis: true location is not equal to 0
```

Zwischen den Gruppen gibt es zu beiden Zeitpunkten keinen Unterschied. Es kann allerdings eine Verbesserung des Scores über die Zeit festgestellt werden (grafisch und per Test).

5.3 Beweglichkeit / Bewegungsdefizite

Vor der Analyse wurde die Differenz zwischen der Trauma Hand und der gesunden Hand berechnet. Dabei sagen negative Werte aus, dass an der Trauma Hand eine geringere Beweglichkeit vorliegt als an der gesunden Hand. Der Zweistichproben Welch t-Test wurde für den Mittelwert-Vergleich zwischen Gruppe A und B für beide Zeitpunkte durchgeführt. Der Einstichproben t-Test wurde jeweils für Gruppe A und B sowie jeweils zu den beiden Zeitpunkten angewendet, um zu testen, ob sich der Mittelwert der Bewegungsdifferenz von 0 unterscheidet (Nullhypothese: keine Beweglichkeitsunterschied zwischen gesunder und traumatiseriter Hand). Um die zeitliche Änderung innerhalb der Gruppen A und B zu testen wurde der gepaarte t-Test eingesetzt.

5.4 Bewegungsdefizite: D.Ext

```
Gruppenvergleich: Gruppe A zu Gruppe B
======================================

1 Monat pOP

Welch Two Sample t-test

data:  D.Ext by Gruppe
t = 0.6959, df = 22.24, p-value = 0.4937
alternative hypothesis: true difference in means is not equal to 0
95 percent confidence interval:
 -8.055 16.198
sample estimates:
mean in group Gruppe A mean in group Gruppe B
                 33.00                  28.93



3 Monate pOP

Welch Two Sample t-test

data:  D.Ext by Gruppe
t = 0.8379, df = 17.78, p-value = 0.4132
alternative hypothesis: true difference in means is not equal to 0
95 percent confidence interval:
 -5.248 12.200
sample estimates:
mean in group Gruppe A mean in group Gruppe B
                 6.333                  2.857



Handvergleich kranke Hand zur Gesunden
======================================
Gruppe A
Beweglichkeit 1 Monat pOP
-----------------------------------------------------------

Beweglichkeit 1 Monat pOP versus 3 Monate pOP
Paired t-test

data:  D.Ext by Woche
t = 5.647, df = 14, p-value = 6.026e-05
alternative hypothesis: true difference in means is not equal to 0
95 percent confidence interval:
```

```
 16.54 36.80
sample estimates:
mean of the differences
                  26.67

Beweglichkeit 1 Monate pOP: Unterschied zur 0

One Sample t-test

data:  eval(parse(text = paste("subset(dataBEW, Woche == \"1 Monat pOP\" & Gruppe == \"Gruppe A\")$"
t = 6.514, df = 14, p-value = 1.37e-05
alternative hypothesis: true mean is not equal to 0
95 percent confidence interval:
 22.13 43.87
sample estimates:
mean of x
       33

Beweglichkeit 3 Monate pOP: Unterschied zur 0

One Sample t-test

data:  eval(parse(text = paste("subset(dataBEW, Woche == \"3 Monate pOP\" & Gruppe == \"Gruppe A\")$
t = 3.833, df = 14, p-value = 0.001828
alternative hypothesis: true mean is not equal to 0
95 percent confidence interval:
 2.789 9.877
sample estimates:
mean of x
    6.333


Gruppe B
Beweglichkeit 1 Monat pOP
-----------------------------------------------------------

Beweglichkeit 1 Monat pOP versus 3 Monate pOP
Paired t-test

data:  D.Ext by Woche
t = 6.886, df = 13, p-value = 1.108e-05
alternative hypothesis: true difference in means is not equal to 0
95 percent confidence interval:
 17.89 34.25
sample estimates:
mean of the differences
                  26.07

Beweglichkeit 1 Monat pOP: Unterschied zur 0

One Sample t-test

data:  eval(parse(text = paste("subset(dataBEW, Woche == \"1 Monat pOP\" & Gruppe == \"Gruppe B\")$"
t = 9.884, df = 13, p-value = 2.059e-07
alternative hypothesis: true mean is not equal to 0
95 percent confidence interval:
 22.61 35.25
sample estimates:
```

```
mean of x
    28.93

Beweglichkeit 3 Monate pOP: Unterschied zur 0

One Sample t-test

data:  eval(parse(text = paste("subset(dataBEW, Woche == \"3 Monate pOP\" & Gruppe == \"Gruppe B\")$
t = 0.7508, df = 13, p-value = 0.4662
alternative hypothesis: true mean is not equal to 0
95 percent confidence interval:
 -5.364 11.078
sample estimates:
mean of x
    2.857
```

5.5 Bewegungsdefizite: P.Flex

```
Gruppenvergleich: Gruppe A zu Gruppe B
======================================

1 Monat pOP

Welch Two Sample t-test

data:  P.Flex by Gruppe
t = 2.122, df = 25.92, p-value = 0.04359
alternative hypothesis: true difference in means is not equal to 0
95 percent confidence interval:
  0.3476 22.0810
sample estimates:
mean in group Gruppe A mean in group Gruppe B
                 38.00                  26.79



3 Monate pOP

Welch Two Sample t-test

data:  P.Flex by Gruppe
t = 1.366, df = 25.42, p-value = 0.1838
alternative hypothesis: true difference in means is not equal to 0
95 percent confidence interval:
 -2.783 13.783
sample estimates:
mean in group Gruppe A mean in group Gruppe B
                  13.0                    7.5



Handvergleich kranke Hand zur Gesunden
======================================
Gruppe A
Beweglichkeit 1 Monat pOP
-----------------------------------------------------------
```

```
Beweglichkeit 1 Monat pOP versus 3 Monate pOP
Paired t-test

data:  P.Flex by Woche
t = 6.168, df = 14, p-value = 2.443e-05
alternative hypothesis: true difference in means is not equal to 0
95 percent confidence interval:
 16.31 33.69
sample estimates:
mean of the differences
                     25

Beweglichkeit 1 Monate pOP: Unterschied zur 0

One Sample t-test

data:  eval(parse(text = paste("subset(dataBEW, Woche == \"1 Monat pOP\" & Gruppe == \"Gruppe A\")$"
t = 9.127, df = 14, p-value = 2.859e-07
alternative hypothesis: true mean is not equal to 0
95 percent confidence interval:
 29.07 46.93
sample estimates:
mean of x
       38

Beweglichkeit 3 Monate pOP: Unterschied zur 0

One Sample t-test

data:  eval(parse(text = paste("subset(dataBEW, Woche == \"3 Monate pOP\" & Gruppe == \"Gruppe A\")$
t = 5.146, df = 14, p-value = 0.0001485
alternative hypothesis: true mean is not equal to 0
95 percent confidence interval:
  7.582 18.418
sample estimates:
mean of x
       13


Gruppe B
Beweglichkeit 1 Monat pOP
-----------------------------------------------------------

Beweglichkeit 1 Monat pOP versus 3 Monate pOP
Paired t-test

data:  P.Flex by Woche
t = 4.5, df = 13, p-value = 0.0005973
alternative hypothesis: true difference in means is not equal to 0
95 percent confidence interval:
 10.03 28.54
sample estimates:
mean of the differences
                  19.29

Beweglichkeit 1 Monat pOP: Unterschied zur 0
```

```
One Sample t-test

data:  eval(parse(text = paste("subset(dataBEW, Woche == \"1 Monat pOP\" & Gruppe == \"Gruppe B\")$"
t = 8.225, df = 13, p-value = 1.649e-06
alternative hypothesis: true mean is not equal to 0
95 percent confidence interval:
 19.75 33.82
sample estimates:
mean of x
    26.79

Beweglichkeit 3 Monate pOP: Unterschied zur 0

One Sample t-test

data:  eval(parse(text = paste("subset(dataBEW, Woche == \"3 Monate pOP\" & Gruppe == \"Gruppe B\")$
t = 2.393, df = 13, p-value = 0.0325
alternative hypothesis: true mean is not equal to 0
95 percent confidence interval:
  0.7296 14.2704
sample estimates:
mean of x
      7.5
```

5.6 Bewegungsdefizite: Sup

```
Gruppenvergleich: Gruppe A zu Gruppe B
======================================

1 Monat pOP

Welch Two Sample t-test

data:  Sup by Gruppe
t = -0.2859, df = 26.95, p-value = 0.7771
alternative hypothesis: true difference in means is not equal to 0
95 percent confidence interval:
 -25.12  18.97
sample estimates:
mean in group Gruppe A mean in group Gruppe B
                 33.00                  36.07



3 Monate pOP

Welch Two Sample t-test

data:  Sup by Gruppe
t = 0, df = 25.4, p-value = 1
alternative hypothesis: true difference in means is not equal to 0
95 percent confidence interval:
 -13.5  13.5
sample estimates:
mean in group Gruppe A mean in group Gruppe B
                     5                      5
```

```
Handvergleich kranke Hand zur Gesunden
======================================
Gruppe A
Beweglichkeit 1 Monat pOP
-----------------------------------------------------------

Beweglichkeit 1 Monat pOP versus 3 Monate pOP
Paired t-test

data:  Sup by Woche
t = 4.597, df = 14, p-value = 0.0004146
alternative hypothesis: true difference in means is not equal to 0
95 percent confidence interval:
 14.94 41.06
sample estimates:
mean of the differences
                     28

Beweglichkeit 1 Monate pOP: Unterschied zur 0

One Sample t-test

data:  eval(parse(text = paste("subset(dataBEW, Woche == \"1 Monat pOP\" & Gruppe == \"Gruppe A\")$"
t = 4.18, df = 14, p-value = 0.0009263
alternative hypothesis: true mean is not equal to 0
95 percent confidence interval:
 16.07 49.93
sample estimates:
mean of x
       33

Beweglichkeit 3 Monate pOP: Unterschied zur 0

One Sample t-test

data:  eval(parse(text = paste("subset(dataBEW, Woche == \"3 Monate pOP\" & Gruppe == \"Gruppe A\")$
t = 1.216, df = 14, p-value = 0.2441
alternative hypothesis: true mean is not equal to 0
95 percent confidence interval:
 -3.818 13.818
sample estimates:
mean of x
        5


Gruppe B
Beweglichkeit 1 Monat pOP
-----------------------------------------------------------

Beweglichkeit 1 Monat pOP versus 3 Monate pOP
Paired t-test

data:  Sup by Woche
t = 4.492, df = 13, p-value = 0.0006064
alternative hypothesis: true difference in means is not equal to 0
```

```
95 percent confidence interval:
 16.13 46.02
sample estimates:
mean of the differences
                  31.07

Beweglichkeit 1 Monat pOP: Unterschied zur 0

One Sample t-test

data:  eval(parse(text = paste("subset(dataBEW, Woche == \"1 Monat pOP\" & Gruppe == \"Gruppe B\")$"
t = 4.952, df = 13, p-value = 0.0002647
alternative hypothesis: true mean is not equal to 0
95 percent confidence interval:
 20.33 51.81
sample estimates:
mean of x
    36.07

Beweglichkeit 3 Monate pOP: Unterschied zur 0

One Sample t-test

data:  eval(parse(text = paste("subset(dataBEW, Woche == \"3 Monate pOP\" & Gruppe == \"Gruppe B\")$
t = 0.9787, df = 13, p-value = 0.3456
alternative hypothesis: true mean is not equal to 0
95 percent confidence interval:
 -6.037 16.037
sample estimates:
mean of x
        5
```

5.7 Bewegungsdefizite: Pron

```
Gruppenvergleich: Gruppe A zu Gruppe B
======================================

1 Monat pOP

Welch Two Sample t-test

data:  Pron by Gruppe
t = -0.1787, df = 26.5, p-value = 0.8595
alternative hypothesis: true difference in means is not equal to 0
95 percent confidence interval:
 -18.44  15.49
sample estimates:
mean in group Gruppe A mean in group Gruppe B
                 15.67                  17.14




3 Monate pOP

Welch Two Sample t-test
```

```
data:  Pron by Gruppe
t = 1.792, df = 24.32, p-value = 0.08557
alternative hypothesis: true difference in means is not equal to 0
95 percent confidence interval:
 -0.3053  4.3530
sample estimates:
mean in group Gruppe A mean in group Gruppe B
                1.6667                -0.3571



Handvergleich kranke Hand zur Gesunden
======================================
Gruppe A
Beweglichkeit 1 Monat pOP
-----------------------------------------------------------

Beweglichkeit 1 Monat pOP versus 3 Monate pOP
Paired t-test

data:  Pron by Woche
t = 2.673, df = 14, p-value = 0.01819
alternative hypothesis: true difference in means is not equal to 0
95 percent confidence interval:
  2.767 25.233
sample estimates:
mean of the differences
                     14

Beweglichkeit 1 Monate pOP: Unterschied zur 0

One Sample t-test

data:  eval(parse(text = paste("subset(dataBEW, Woche == \"1 Monat pOP\" & Gruppe == \"Gruppe A\")$"
t = 2.828, df = 14, p-value = 0.01341
alternative hypothesis: true mean is not equal to 0
95 percent confidence interval:
  3.786 27.547
sample estimates:
mean of x
    15.67

Beweglichkeit 3 Monate pOP: Unterschied zur 0

One Sample t-test

data:  eval(parse(text = paste("subset(dataBEW, Woche == \"3 Monate pOP\" & Gruppe == \"Gruppe A\")$
t = 1.784, df = 14, p-value = 0.09614
alternative hypothesis: true mean is not equal to 0
95 percent confidence interval:
 -0.3373  3.6707
sample estimates:
mean of x
    1.667


Gruppe B
Beweglichkeit 1 Monat pOP
```

```
-----------------------------------------------------------

Beweglichkeit 1 Monat pOP versus 3 Monate pOP
Paired t-test

data:  Pron by Woche
t = 3.014, df = 13, p-value = 0.009976
alternative hypothesis: true difference in means is not equal to 0
95 percent confidence interval:
  4.955 30.045
sample estimates:
mean of the differences
                   17.5

Beweglichkeit 1 Monat pOP: Unterschied zur 0

One Sample t-test

data:  eval(parse(text = paste("subset(dataBEW, Woche == \"1 Monat pOP\" & Gruppe == \"Gruppe B\")$"
t = 2.797, df = 13, p-value = 0.01511
alternative hypothesis: true mean is not equal to 0
95 percent confidence interval:
  3.903 30.383
sample estimates:
mean of x
    17.14

Beweglichkeit 3 Monate pOP: Unterschied zur 0

One Sample t-test

data:  eval(parse(text = paste("subset(dataBEW, Woche == \"3 Monate pOP\" & Gruppe == \"Gruppe B\")$
t = -0.5631, df = 13, p-value = 0.583
alternative hypothesis: true mean is not equal to 0
95 percent confidence interval:
 -1.727  1.013
sample estimates:
mean of x
  -0.3571
```

5.8 Bewegungsdefizite: R.Dukt

```
Gruppenvergleich: Gruppe A zu Gruppe B
======================================

1 Monat pOP

Welch Two Sample t-test

data:  R.Dukt by Gruppe
t = 1.791, df = 20.03, p-value = 0.08844
alternative hypothesis: true difference in means is not equal to 0
95 percent confidence interval:
 -0.9842 12.9365
sample estimates:
mean in group Gruppe A mean in group Gruppe B
                11.333                  5.357
```

```
3 Monate pOP

Welch Two Sample t-test

data:  R.Dukt by Gruppe
t = 0.3659, df = 26, p-value = 0.7174
alternative hypothesis: true difference in means is not equal to 0
95 percent confidence interval:
 -3.079  4.412
sample estimates:
mean in group Gruppe A mean in group Gruppe B
                0.6667                 0.0000



Handvergleich kranke Hand zur Gesunden
======================================
Gruppe A
Beweglichkeit 1 Monat pOP
-----------------------------------------------------------

Beweglichkeit 1 Monat pOP versus 3 Monate pOP
Paired t-test

data:  R.Dukt by Woche
t = 6.631, df = 14, p-value = 1.13e-05
alternative hypothesis: true difference in means is not equal to 0
95 percent confidence interval:
  7.217 14.117
sample estimates:
mean of the differences
                  10.67

Beweglichkeit 1 Monate pOP: Unterschied zur 0

One Sample t-test

data:  eval(parse(text = paste("subset(dataBEW, Woche == \"1 Monat pOP\" & Gruppe == \"Gruppe A\")$"
t = 7.179, df = 14, p-value = 4.715e-06
alternative hypothesis: true mean is not equal to 0
95 percent confidence interval:
  7.948 14.719
sample estimates:
mean of x
    11.33

Beweglichkeit 3 Monate pOP: Unterschied zur 0

One Sample t-test

data:  eval(parse(text = paste("subset(dataBEW, Woche == \"3 Monate pOP\" & Gruppe == \"Gruppe A\")$
t = 0.5641, df = 14, p-value = 0.5816
alternative hypothesis: true mean is not equal to 0
95 percent confidence interval:
 -1.868  3.202
```

```
sample estimates:
mean of x
   0.6667


Gruppe B
Beweglichkeit 1 Monat pOP
-----------------------------------------------------------

Beweglichkeit 1 Monat pOP versus 3 Monate pOP
Paired t-test

data:  R.Dukt by Woche
t = 1.627, df = 13, p-value = 0.1277
alternative hypothesis: true difference in means is not equal to 0
95 percent confidence interval:
 -1.756 12.471
sample estimates:
mean of the differences
                  5.357

Beweglichkeit 1 Monat pOP: Unterschied zur 0

One Sample t-test

data:  eval(parse(text = paste("subset(dataBEW, Woche == \"1 Monat pOP\" & Gruppe == \"Gruppe B\")$"
t = 1.822, df = 13, p-value = 0.09152
alternative hypothesis: true mean is not equal to 0
95 percent confidence interval:
 -0.9945 11.7088
sample estimates:
mean of x
    5.357

Beweglichkeit 3 Monate pOP: Unterschied zur 0

One Sample t-test

data:  eval(parse(text = paste("subset(dataBEW, Woche == \"3 Monate pOP\" & Gruppe == \"Gruppe B\")$
t = 0, df = 13, p-value = 1
alternative hypothesis: true mean is not equal to 0
95 percent confidence interval:
 -2.996  2.996
sample estimates:
mean of x
        0
```

5.9 Bewegungsdefizite: U.Dukt

```
Gruppenvergleich: Gruppe A zu Gruppe B
======================================

1 Monat pOP

Welch Two Sample t-test
```

```
data:  U.Dukt by Gruppe
t = -1.178, df = 24.06, p-value = 0.2502
alternative hypothesis: true difference in means is not equal to 0
95 percent confidence interval:
 -13.756   3.756
sample estimates:
mean in group Gruppe A mean in group Gruppe B
                    10                     15



3 Monate pOP

Welch Two Sample t-test

data:  U.Dukt by Gruppe
t = 0.5474, df = 26.99, p-value = 0.5886
alternative hypothesis: true difference in means is not equal to 0
95 percent confidence interval:
 -3.534  6.105
sample estimates:
mean in group Gruppe A mean in group Gruppe B
                2.0000                 0.7143



Handvergleich kranke Hand zur Gesunden
======================================
Gruppe A
Beweglichkeit 1 Monat pOP
-----------------------------------------------------------

Beweglichkeit 1 Monat pOP versus 3 Monate pOP
Paired t-test

data:  U.Dukt by Woche
t = 3.434, df = 14, p-value = 0.004034
alternative hypothesis: true difference in means is not equal to 0
95 percent confidence interval:
  3.003 12.997
sample estimates:
mean of the differences
                      8

Beweglichkeit 1 Monate pOP: Unterschied zur 0

One Sample t-test

data:  eval(parse(text = paste("subset(dataBEW, Woche == \"1 Monat pOP\" & Gruppe == \"Gruppe A\")$"
t = 4.019, df = 14, p-value = 0.001267
alternative hypothesis: true mean is not equal to 0
95 percent confidence interval:
  4.664 15.336
sample estimates:
mean of x
       10

Beweglichkeit 3 Monate pOP: Unterschied zur 0
```

```
One Sample t-test

data:  eval(parse(text = paste("subset(dataBEW, Woche == \"3 Monate pOP\" & Gruppe == \"Gruppe A\")$
t = 1.193, df = 14, p-value = 0.2526
alternative hypothesis: true mean is not equal to 0
95 percent confidence interval:
 -1.595  5.595
sample estimates:
mean of x
        2


Gruppe B
Beweglichkeit 1 Monat pOP
-----------------------------------------------------------

Beweglichkeit 1 Monat pOP versus 3 Monate pOP
Paired t-test

data:  U.Dukt by Woche
t = 3.647, df = 13, p-value = 0.002955
alternative hypothesis: true difference in means is not equal to 0
95 percent confidence interval:
  5.823 22.749
sample estimates:
mean of the differences
                  14.29

Beweglichkeit 1 Monat pOP: Unterschied zur 0

One Sample t-test

data:  eval(parse(text = paste("subset(dataBEW, Woche == \"1 Monat pOP\" & Gruppe == \"Gruppe B\")$"
t = 4.364, df = 13, p-value = 0.0007666
alternative hypothesis: true mean is not equal to 0
95 percent confidence interval:
  7.575 22.425
sample estimates:
mean of x
       15

Beweglichkeit 3 Monate pOP: Unterschied zur 0

One Sample t-test

data:  eval(parse(text = paste("subset(dataBEW, Woche == \"3 Monate pOP\" & Gruppe == \"Gruppe B\")$
t = 0.4341, df = 13, p-value = 0.6714
alternative hypothesis: true mean is not equal to 0
95 percent confidence interval:
 -2.841  4.269
sample estimates:
mean of x
   0.7143
```
